# Systems Bioinformatics Reveals Possible Relationship between COVID-19 and the Development of Neurological Diseases and Neuropsychiatric Disorders

**DOI:** 10.3390/v14102270

**Published:** 2022-10-16

**Authors:** Anna Onisiforou, George M. Spyrou

**Affiliations:** Bioinformatics Department, The Cyprus Institute of Neurology & Genetics, Nicosia 2370, Cyprus

**Keywords:** COVID-19, SARS-CoV-2, neurological diseases, neuropsychiatric disorders, neurodegenerative diseases, molecular mimicry, autoimmunity, epitopes, systems bioinformatics, network biology

## Abstract

Coronavirus Disease 2019 (COVID-19) is associated with increased incidence of neurological diseases and neuropsychiatric disorders after infection, but how it contributes to their development remains under investigation. Here, we investigate the possible relationship between COVID-19 and the development of ten neurological disorders and three neuropsychiatric disorders by exploring two pathological mechanisms: (i) dysregulation of host biological processes via virus–host protein–protein interactions (PPIs), and (ii) autoreactivity of severe acute respiratory syndrome coronavirus 2 (SARS-CoV-2) epitopes with host “self” proteins via molecular mimicry. We also identify potential genetic risk factors which in combination with SARS-CoV-2 infection might lead to disease development. Our analysis indicated that neurodegenerative diseases (NDs) have a higher number of disease-associated biological processes that can be modulated by SARS-CoV-2 via virus–host PPIs than neuropsychiatric disorders. The sequence similarity analysis indicated the presence of several matching 5-mer and/or 6-mer linear motifs between SARS-CoV-2 epitopes with autoreactive epitopes found in Alzheimer’s Disease (AD), Parkinson’s Disease (PD), Myasthenia Gravis (MG) and Multiple Sclerosis (MS). The results include autoreactive epitopes that recognize amyloid-beta precursor protein (APP), microtubule-associated protein tau (MAPT), acetylcholine receptors, glial fibrillary acidic protein (GFAP), neurofilament light polypeptide (NfL) and major myelin proteins. Altogether, our results suggest that there might be an increased risk for the development of NDs after COVID-19 both via autoreactivity and virus–host PPIs.

## 1. Introduction

Viruses, since long before the COVID-19 pandemic, have been known to cause diseases. Several viruses are associated as risk factors in the development of various NDs, autoimmune diseases and neuropsychiatric disorders [1,2,3,4]. Neurotropic viruses, particularly members of the Herpesviridae family, have a strong association with the development of NDs [5]. SARS-CoV-2 is also a neurotropic virus that can invade the central nervous system (CNS) and can elicit a strong immune response [6,7,8]. Therefore, similarly to other viral infections, SARS-CoV-2 might also lead to the development of viral-associated comorbidities.

Evidence indicates that patients diagnosed with COVID-19 have increased incidence of diagnosis of neurological diseases and neuropsychiatric disorders 6 months after infection, with patients admitted to the intensive therapy unit having even greater incidence risk [9]. The development of neurological symptoms during the acute phase is observed in approximately one third of the infected cases [10]. However, the exact pathological mechanisms by which COVID-19 leads to the development of these symptoms is still under investigation.

SARS-CoV-2 infection can also trigger a cytokine storm in some patients with severe infection, which is associated with excessive circulating levels of pro-inflammatory cytokines and increased mortality rates [7]. Neuroinflammation is a central feature of neurological diseases and neuropsychiatric disorders, therefore the neuroinvasive ability of SARS-CoV-2 in combination with its ability to trigger inflammation can possibly lead to development or progression of these diseases/disorders [11,12,13]. Evidence suggests that SARS-CoV-2 infection can lead to the rapid progression of certain NDs, such as ALS, resulting in worsening of symptoms [14]. Therefore, we can ask whether COVID-19 can lead to the development of neurological disease and neuropsychiatric disorders and by which mechanisms?

Viruses can lead to the development of diseases through both direct and indirect effects with the two main pathological mechanisms being through virus–host PPIs and autoreactivity. Viruses lack their own replication machinery, hence by interacting with the human host, primarily via virus–host PPIs, exploit host components for their own advantage [15,16]. These physical interactions can lead to dysregulation of biological processes resulting in the emergence of various complex diseases. Analysis of virus–host interactions using network-based approaches has been extensively utilized to provide insight into viral pathogenicity [17,18,19,20,21,22]. In our previous works, we have used virus–host PPIs to identify viral-mediated pathogenic mechanisms by which viruses associated with the development of NDs might lead to their development [23,24].

A prevailing hypothesis is that infectious agents can trigger autoreactivity via molecular mimicry, where immunological memory cells (T and B cells) that recognize viral epitopes mistakenly target self-proteins [25,26,27]. Molecular mimicry occurs when similarity exists between pathogen and host molecules, that can be either present in the linear amino acid (AA) sequence of the two molecules or in their conformational structure [28]. For cross-reactivity to occur, the AA sequence homology does not need to be identical, but rather close enough, containing important conserved motifs with immunogenic host determinants [29].

However, viruses are very common in the general population, hence infection with a virus is not a sufficient factor by itself for disease development [30]. The “multiple hit” hypothesis suggests that the development of complex diseases is of multifactorial origin and requires the combinatorial action of multiple environmental and/or genetic risk factors [31]. Genetic susceptibility is an important risk factor that determines how an individual’s immune system responds to viral threats [30,32]. Therefore, the presence of a disease susceptibility gene in combination with an infectious disease associated with its emergence can be a lethal interplay for disease development. It is thus important to identify genetic factors which in combination with SARS-CoV-2 infection can increase disease susceptibility risk.

**Figure 1:**
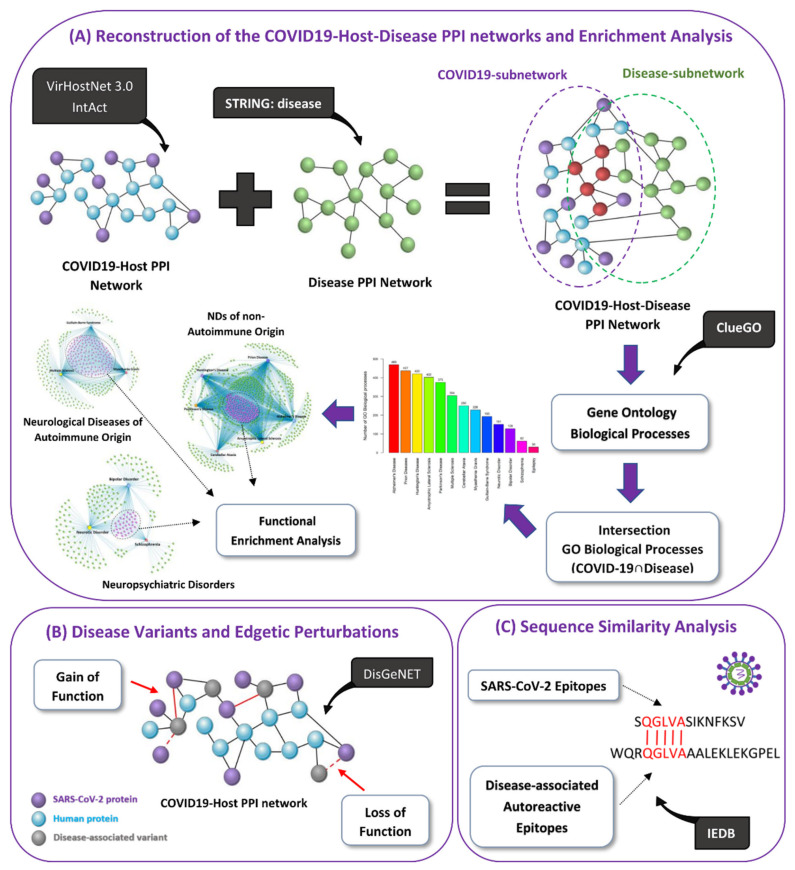
**Schematic Representation of the Methodology used in this Study.** The aim was to identify potential genetic risk factors and possible pathological mechanisms by which COVID-19 might lead to the development of neurological diseases and neuropsychiatric disorders. (**A**) We represent the various data sources used to reconstruct the COVID-19-Host-Disease PPI networks and the methodology used to identify overlapping Gene Ontology (GO) biological processes (COVID-19 ∩ Disease) between COVID-19 and each of the thirteen included diseases/disorders, by using enrichment analysis. We also identify common COVID-19-mediated pathological mechanisms between COVID-19 and groups of diseases/disorders. (**B**) We also identify possible disease-associated genetic variants that could lead to the emergence of edgetic perturbations in COVID-19-host PPIs. Purple nodes represent SARS-CoV-2 viral proteins, blue nodes human proteins and gray nodes represent human proteins that have genetic variants associated with disease development and are also targeted by SARS-CoV-2 viral proteins. (**C**) Finally, we perform pairwise sequence similarity analysis between SARS-CoV-2 epitopes and autoreactive epitopes found in neurological diseases to identify the presence of 5-mer and above matching linear motifs in their consensus sequence, with the aim to determine the potential risk of autoreactivity of SARS-CoV-2 epitopes with human proteins.

Here, we first use network-based approaches, as illustrated in Figure 1, to identify biological processes by which SARS-CoV-2 via virus–host PPIs might lead to the development or worsening of symptoms in existing patients of ten neurological diseases (Amyotrophic Lateral Sclerosis (ALS), Prion Disease, Huntington’s Disease (HD), AD, PD, MG, MS, Cerebellar Ataxia (CA), Epilepsy, Guillain-Barré syndrome (GBS)) and three neuropsychiatric disorders (Neurotic Disorder, Bipolar Disorder (BD), Schizophrenia). Our hypothesis is that neurological diseases and neuropsychiatric disorders whose development and/or progression has been previously associated with viral infections will have a higher probability to be triggered by a new emerging virus (SARS-CoV-2), than other non-viral associated diseases/disorders. Thus, these specific diseases/disorders were selected because an infectious disease has been associated as a risk factor for their development/progression and/or cases studies indicated that COVID-19 can possibly trigger their development/progression. We also identify common biological processes that might be modulated by SARS-CoV-2 in the included diseases/disorders by grouping them based on their main pathological characteristics: (i) neurological diseases of autoimmune origin, (ii) NDs of non-autoimmune origin and (iii) neuropsychiatric disorders. We also identify disease-associated susceptibility genes whose presence in certain individuals in combination with SARS-CoV-2 infection could lead to the emergence of edgetic perturbations, i.e., changes in edge interactions between nodes, resulting in altered virus–host PPIs. Thus, predisposing an individual to the emergence of these disease phenotypes. Moreover, we determined the autoreactivity potential of SARS-CoV-2 epitopes, via molecular mimicry, by performing pairwise sequence similarity with autoreactive epitopes found in neurological diseases.

## 2. Methods

Our pipeline, which is illustrated in Figure 1, consists of the following approaches: (A) Reconstruction of the COVID-19-Host-Disease PPI networks and Enrichment Analysis, (B) Disease Variants and Edgetic Perturbations and (C) Sequence Similarity Analysis.

### 2.1. Network Reconstruction

#### 2.1.1. Reconstruction of the COVID-19-Host PPI Network

Experimentally validated virus–host PPI data between SARS-CoV-2 and the human host were obtained from VirHostNet 3.0 [33] and IntAct [34] databases (date 16 September 2021), where only physical interactions with available UniProt ID were retained. After removing duplicated entries, 1014 COVID-19-host PPIs were collected between 15 SARS-CoV-2 proteins and 861 human proteins, that were used to reconstruct the COVID-19-host PPI network. Based on its genome, SARS-CoV-2 is estimated to encode for at least 26 viral proteins [35], however virus–host PPIs were not available for all of its proteins.

#### 2.1.2. Reconstruction of the Integrated COVID-19-Host-Disease PPI Networks

To identify disease-associated biological processes that might be dysregulated by SARS-CoV-2 infection in existing patients with neurological diseases/neuropsychiatric disorders or possibly lead to their development, we generated thirteen integrated COVID-19-host-disease PPI networks. Using the same approach as in our previous work [23,24], we used the *String: disease app* in Cytoscape to obtain the top 200 disease-associated proteins with the highest disease score, for each of the following neuropsychiatric disorders and neurological diseases: ALS (ID: 332), Prion Disease (ID:649), HD (ID:12858), PD (ID:14330), MS (ID: 2377), CA (ID: 0050753), MG (ID: 437), GBS (ID:12842), AD (ID:10652), Neurotic Disorder (ID: 4964), BD (ID: 3312), Schizophrenia (ID: 5419) and Epilepsy (ID: 1826). The confidence cut-off score of the interactions between the human proteins was set at 0.8. To generate each of the integrated networks we merged the COVID-19-host PPI network with each of the disease-related PPI networks, that contained the 200 disease-associated proteins for each disease/disorder.

### 2.2. Enrichment Analysis

#### 2.2.1. SARS-CoV-2 Human Protein Targets

To identify pathways that can be modulated by SARS-CoV-2 viral proteins we performed enrichment analysis on their 861 human protein targets by using the ClueGO app [36] in Cytoscape. To perform the enrichment analysis, we used the KEGG database, keeping only significant enriched terms with adjusted *p*-value ≤ 0.05 (corrected with Benjamini–Hochberg).

#### 2.2.2. Overlapping GO Biological Processes (COVID-19 ∩ Disease)

To identify viral-mediated pathogenic mechanisms by which SARS-CoV-2 can possibly lead to the development of the 13 diseases/disorders we first isolated from each of the 13 integrated COVID-19-disease PPIs networks two subnetworks that would allow to identify overlapping biological processes (COVID-19 ∩ Disease). For each integrated networks we extracted the (i) COVID-19 subnetwork, which includes the 861 human protein targets of SARS-CoV-2 viral proteins and their first neighbors and (ii) the disease-related subnetworks, which includes the 200 disease-associated proteins and their first neighbors. Then, we performed enrichment analysis on each of the subnetworks by using the GO-biological processes database, retaining only significant processes that had an adjusted *p*-value ≤ 0.01 (corrected with Bonferroni step-down). For the enrichment analysis of the extracted subnetworks, we used Bonferroni step-down because it provides a stricter result when the sample size of proteins used for analysis is large, whereas in Section 2.2.1 we used Benjamini–Hochberg because the number of proteins included for analysis was significantly smaller. In addition, in this part of the analysis we selected a stricter *p*-value, than in Section 2.2.1, because the GO Biological Processes database contains a significant larger number of terms, having 17,400 terms, whereas KEGG databases only contains 335 terms/pathways.

After obtaining the significant GO biological processes for each subnetwork, we removed any false positives enriched GO biological processes results by eliminating enriched terms that only contained term-associated genes that derived from only COVID-19 or only the disease-associated genes. Therefore, each of the GO biological processes terms that were retained for each subnetwork had to contain at least one gene-associated with both COVID-19 and the disease/disorder.

Then, by using Venn diagrams, we identified overlapping GO biological processes between the COVID-19 subnetwork and each of the disease-related subnetworks enriched results. Finally, by using the preselected function in *ClueGO* app, we performed functional enrichment analysis in the isolated GO biological processes (COVID-19 ∩ Disease) to identify the functional groups that these biological processes belong to.

### 2.3. Identification of Common GO Biological Processes Affected by COVID-19 in a Group of Diseases/Disorders

To identify common GO biological processes that might be affected by COVID-19 in the thirteen diseases/disorders we classified them into three groups based on their main pathological characteristics: (i) neurological diseases of autoimmune origin (ii) NDs of non-autoimmune origin and (iii) neuropsychiatric disorders. The neurological diseases of autoimmune origin group includes MS, MG and GBS, which are diseases in which autoreactivity represents a main pathological characteristic [37,38,39], whereas the NDs of non-autoimmune origin group includes ALS, HD, PD, AD, Prion Disease and CA, in which, although autoreactivity can be found in some cases, it does not represent a main pathological characteristic. In addition, the neuropsychiatric disorders group includes BD, Neurotic Disorder and Schizophrenia. Epilepsy does not fall within any of these groups, therefore, no common GO biological processes affected by SARS-CoV-2 with other diseases are identified; hence, it has been excluded from this part of the analysis.

In order to identify common GO biological processes that might be affected by SARS-CoV-2 in the three groups, we first created for each group a GO biological processes- Diseases network. To construct each of the three networks we included the isolated GO biological processes (COVID-19 ∩ Disease) which were identified to be also modulated by SARS-CoV-2 in each disease/disorder. Then, by using these networks we identified the common GO biological processes that might be affected by SARS-CoV-2 in the three groups. Finally, by using the same approach as in Section 2.2.2, we performed functional enrichment analysis on the common GO biological processes identified in each group to determine the functional groups that these GO biological processes belong to.

### 2.4. Identification of Disease-Associated Variants Targeted by SARS-CoV-2

Genetic susceptibility is an important risk factor that not only predisposes an individual towards the development of complex diseases, such as NDs, but also determines infectious disease outcome. Host genetics that are involved in viral defenses or the life cycle of the virus can influence viral exposure and disease outcome. Understanding the interaction between host genetics and infectious diseases can aid to elucidate the role of genetic variability in viral pathogenicity and infectious disease susceptibility [40]. Genetic susceptibility is an important factor that determines the sensitivity of an individual towards different pathogens as it alters virus–host symbiosis and hence virus–host interactions [23]. This results in altered host responses, neurovirulence and even shift in viral tropism which can pre-dispose an individual towards viral-associated diseases [41,42,43]. In addition, genetic variation of the human host is an important environmental factor that acts as a selective pressure affecting virus evolution, which in turns affects viral-host dynamics [44]. Therefore, genetic diversity of either viral or host proteins or both can result in changes in virus–host symbiosis, leading in the emergence of edgetic perturbations between virus–host PPIs. Edgetic perturbations, can result mainly in four edgetic scenarios: (a) loss of all interactions, (b) partial loss of interactions, (c) gain of one or more interactions, or (d) no loss of interactions [45]. Altered interactions due to either host or viral genetic diversity can thus possibly partially account for the observed high variability of outcomes of a viral infection, such as SARS-CoV-2, and the emergence of different disease phenotypes by the same virus in different individuals.

Neurological diseases and neuropsychiatric disorders are complex conditions whose emergence is hypothesized to require the combinatorial action between genetic and environmental risk factors. According to the local impact hypothesis, disease susceptibility genes are located in proximity of viral host targets of viral-associated diseases within a network [46]. Therefore, if the combinatorial effect between genetic and environmental risk factors contributes to the emergence of viral-associated diseases, we would expect that the interaction of SARS-CoV-2 viral proteins with genetic disease-associated susceptibility genes would alter virus–host PPIs. This would lead to different edgotype scenarios and thus the emergence of different disease phenotypes. As such, we hypothesize that the presence of known diseases -associated variants in certain individuals could result in the emergence of these diseases/disorders, when they get infected with SARS-CoV-2, as it would possibly lead to the emergence of edgetic perturbations between virus–host PPIs, resulting in altered disease pathogenicity.

Therefore, to identify if genetic susceptibility genes of the thirteen diseases/disorders under investigation interact with SARS-CoV-2 viral proteins, we first collected from *DisGeNET* database [47,48] their disease-associated variants, retaining only those with available gene name and removing duplicated gene names. The number of disease-associated variant genes collected for each of the thirteen diseases/disorders is summarized in Table 1. Then, we mapped the collected disease-associated variant genes on the COVID-19-host PPIs network, in order to identify variant genes/proteins for each disease/disorder that are present on the network. HD is an inherited genetic disease involving mutation to the huntingtin (HTT) gene, therefore genetic susceptibility genes where not collected from *DisGeNET*, but the HTT was mapped on the COVID-19-host PPIs network to investigate if SARS-CoV-2 viral proteins interact with the HTT gene.

### 2.5. Cross-Reactivity Potential via Molecular Mimicry of SARS-CoV-2 Epitopes

To investigate the potential of cross-reactivity of SARS-CoV-2 epitopes with human proteins associated with the development of neurological diseases we performed pairwise sequence similarity analysis. Immunogenic epitopes against SARS-CoV-2 found during COVID-19 disease and autoreactive epitopes against human proteins found in the 6 neurological diseases (ALS, PD, AD, MS, MG and GBS) were obtained from IEDB [49]. No autoreactive epitopes were found in IEDB for the rest of the neurological diseases investigated in the paper, hence they have been excluded from this part of the analysis. From the obtained data, we only retained linear epitopes with available Uniprot ID, thus excluding discontinued epitopes from the analysis. Table 2 indicates the twelve immunogenic proteins of SARS-CoV-2 and their respective number of linear epitopes, whereas the number of autoreactive linear epitopes included in the analysis for the 6 neurological diseases, as well as the number of immunogenic proteins that these epitopes recognize, are summarized in Table 3. To prepare the data for pairwise alignment, we had to assign unique identifiers next to the code of each Uniprot ID that the epitope recognizes due to the presence of multiple epitopes from the same protein. For example, there are 3 linear epitopes included in the data that can recognize the ORF7b protein, with Uniprot ID P0DTD8; therefore, to assign unique identifiers, a dash and a unique number were added, i.e., P0DTD8-1, P0DTD8-2 and P0DTD8-3.

Then, we performed pairwise sequence similarity between the SARS-CoV-2 epitopes and the autoreactive epitopes found in each of the 6 neurological diseases, by using the “seqinr” and “Biostrings” packages in R [50,51]. As in this study we were trying to isolate consensus sequences with consecutive AA motifs that might exist between epitopes that recognize SARS-CoV-2 and autoreactive epitopes, we used high gap opening and gap extension penalties, with both parameters set at −10. In addition, because the length between the epitopes to be compared is dissimilar we choose to use the local alignment method which allows to identify the most similar regions between sequences of dissimilar length. To measure their similarity, we used the BLOSUM62 amino acid substitution matrix. Finally, from the pairwise alignment results we isolated only the pairs which in their consensus sequences contained consecutive (linear) motifs of five AAs or more and disregarded discontinuous matching motifs.

## 3. Results

### 3.1. Enrichment Analysis Results of SARS-CoV-2 Human Protein Targets

**Figure 2:**
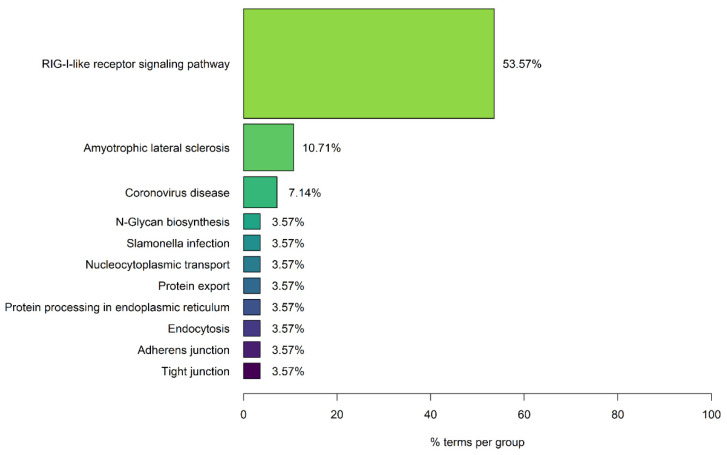
**Enrichment Analysis Results of the 861 Human Protein Targets of SARS-CoV-2 viral proteins.** The enriched KEGG pathways are classified into 11 functional groups, with the percentage indicating the number of terms in each group.

The enrichment analysis results of the 861 human protein targets of the 15 SARS-CoV-2 viral proteins led to the identification of 28 significantly enriched KEGG pathways. These pathways can be grouped into 11 functional groups, as indicated in Figure 2, with 53.57% of the enriched terms belonging to the functional group of RIG-I-like receptor signaling pathway. The retinoic acid-inducible gene I (RIG-I) receptors are key pattern-recognition receptors for the detection of viral infections, by recognizing pathogen associated molecular patterns, which leads to the activation of antiviral immune responses which are critical for the control of viral replication [52]. Viruses, through the evolutionary process, have acquired various evasion mechanisms that allow them to evade host antiviral immune responses, including antagonisms of the RIG-I signaling [52]. Therefore, the interaction of SARS-CoV-2 with the RIG-I-like receptor signaling pathway via virus–host PPIs indicates that it can modulate antiviral host immune responses, which might contribute to the increased pathogenicity observed during COVID-19 disease.

Interestingly, the enrichment results also indicated that 10.71% of the enriched pathways belong to the functional group of Amyotrophic Lateral Sclerosis. The group contains the enriched KEGG pathways ALS, Prion Disease and HD. The enrichment results suggest that SARS-CoV-2 interacts with human proteins associated with neurodegeneration and thus can possibly lead to the development of NDs or affect the severity of symptoms in existing patients.

### 3.2. Impact of SARS-CoV-2 Infection in Neurological Diseases and Neuropsychiatric Disorders

**Figure 3:**
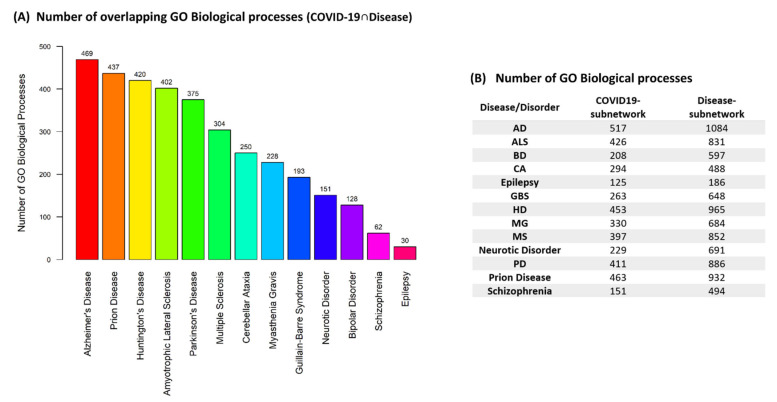
**Effects of SARS-CoV-2, via virus–host PPIs, in Neurological Diseases and Neuropsychiatric Disorders**. (**A**) Number of overlapping GO biological processes (COVID-19 ∩ Disease) between COVID-19 and each disease/disorder. (**B**) Number of GO biological processes found to be associated with the extracted subnetworks from each of the thirteen integrated COVID-19-host-disease PPI networks.

Our methodology allowed to extract the most relevant GO biological processes by which SARS-CoV-2 infection might affect existing neurological and neuropsychiatric patients or lead to their development through dysregulation of biological processes via virus–host PPIs. Figure 3A, indicates the number of overlapping GO biological processes (COVID-19 ∩ Disease) found between the COVID-19 subnetwork and each of the disease-related subnetworks enriched results. The number of GO biological processes found to be associated with the extracted subnetworks for each of the diseases/disorders is indicated in Figure 3B. The functional enrichment results of the overlapping GO biological processes (COVID-19 ∩ Disease) that indicate the functional groups that these biological processes belong to for each disease/disorder can be found in Supplementary Materials.

The results, Figure 3A, show that AD has the highest number of GO biological processes that can be modulated by SARS-CoV-2 through virus–host PPIs, whereas Epilepsy has the lowest number of overlapping GO biological processes. Therefore, the risk for the development of AD due to dysregulation of biological processes via virus–host PPIs, might be higher than other diseases/disorders investigated through our analysis. In addition, the results in Figure 3A indicate that NDs (ALS, Prion Disease, HD, AD, PD, MG, MS, CA) have higher number of GO biological processes that can be modulated by SARS-CoV-2 via virus–host PPIs than neuropsychiatric disorders (Neurotic Disorder, BD, Schizophrenia).

### 3.3. Common Mechanisms of Pathogenesis of SARS-CoV-2 Infection in Neurological Diseases and Neuropsychiatric Disorders

**Figure 4:**
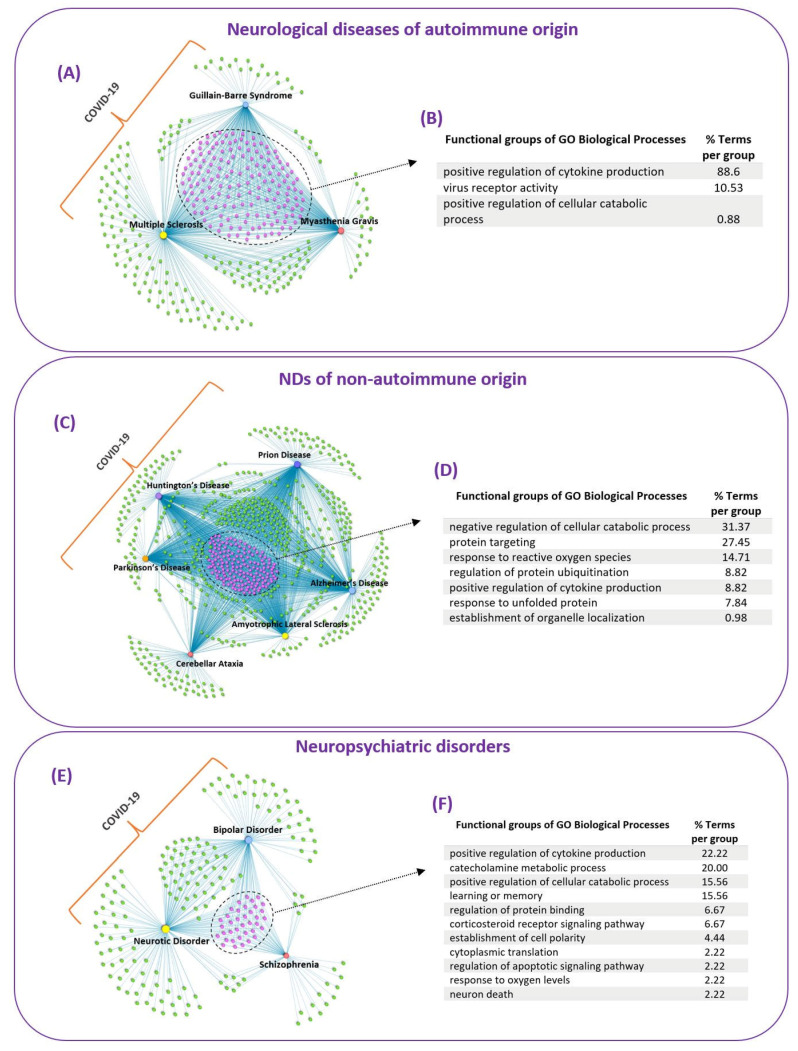
**Integrated Networks of Common GO Biological Processes Affected by COVID-19 in Groups of Diseases/Disorders**. The common GO biological processes are indicated in purple color in the three integrated networks of groups of diseases/disorders, which are classified based on their main pathological characteristics: (i) neurological diseases of autoimmune origin, (ii) NDs of non-autoimmune origin and (iii) neuropsychiatric disorders (**A**,**C**,**E**). Functional groups that the common GO biological processes of each group/network belong to (**B**,**D**,**F**).

To identify common GO biological processes that might be affected by SARS-CoV-2 infection in the thirteen diseases/disorders, we classified them into three groups based on their main pathological characteristics: (i) neurological diseases of autoimmune origin (ii) NDs of non-autoimmune origin and (iii) neuropsychiatric disorders. Epilepsy did not fall within any of these groups; therefore, it was excluded from this part of the analysis. To identify common GO biological processes affected by COVID-19 in each group we created three integrated GO biological processes-Diseases networks, shown in Figure 4A,C,E, that contained the overlapping GO biological processes (COVID-19 ∩ Disease), found for each disease/disorder with COVID-19 (Figure 3A). For the identified common GO biological processes in each group, we also performed functional enrichment analysis to identify the functional groups that these common processes belong to (Figure 4B,D,F).

#### 3.3.1. Neurological Diseases of Autoimmune Origin

The integrated GO biological processes-Diseases network of the three neurological diseases of autoimmune origin (MS, MG and GBS) revealed 157 common GO biological processes affected by COVID-19, Figure 4A. These common biological processes belong in three functional groups, Figure 4B. The functional enrichment results indicated that 88.6% of the terms belong to the group of positive regulation of cytokine production. The pathogenesis of autoimmune diseases, including MS and MG, is associated with abnormal immune responses involving increased production of pro-inflammatory cytokines that contribute to disease development and propagation [53,54]. Thus, the ability of SARS-CoV-2 to modulate the positive regulation of cytokine production, via virus–host PPIs, might represent a pathological mechanism that explains why COVID-19 can lead to neuroinflammation and possibly to the development of neurological diseases of autoimmune origin.

The functional enrichment analysis also indicated that 10.53% of the terms belong to the functional group of virus receptor activity, that involves exogenous protein binding between a virus component and host receptor that mediates the entry of a virus into the cell. It also indicated that 0.88% of the common processes belong to the functional group of positive regulation of catabolic process.

#### 3.3.2. NDs of Non-Autoimmune Origin

The integrated network containing the six NDs of non-autoimmune origin (ALS, AD, PD, HD, Prion Disease and CA) revealed 156 common GO biological processes between this group of diseases and COVID-19 (Figure 4C). These common biological processes belong into seven functional groups, Figure 4D. Almost one third of the terms, 31.37%, belong to the functional group of negative regulation of cellular catabolic process. This involves any process that results in the downregulation of the breakdown of substances, such as cellular, lipid, protein and glycolytic catabolic processes. In addition, 27.45% of the terms belong to the functional group of protein targeting, that involves processes of protein transport and targeting into different cell regions, such as to the peroxisome and to the mitochondrion. Our results are consistent with experimental evidence that indicates that SARS-CoV-2 interferes with protein trafficking in order to suppress host immune defenses [55].

In addition, 14.71% of the terms belong to the functional group of response to reactive oxygen species (ROS), which suggests that COVID-19 can modulate ROS processes. Our finding is consistent with another study by [56] that employed bioinformatics methods to show that SARS-CoV-2 E-protein can capture and neutralize ROS, by converting them into oxygen and water, which allows it to “escape” the immune system [56]. SARS-CoV-2 has also the ability to synthesize ROS, such as superoxide anion and hydrogen peroxide, allowing it to damage multiple organs, including immune organs, thus provoking a strong cytokine storm and organ failure [56]. Overproduction of ROS is cytotoxic resulting in oxidative damage and has been implicated as a pathological mechanism in the development of various diseases, including NDs, as it causes neuronal cell death [57].

Moreover, 8.82% of the common processes belong to the functional group of positive regulation of cytokine production, that involves any process that leads to increased production of cytokines. Neuroinflammation is a common feature in NDs involving increased production of pro-inflammatory cytokines [13,58]. A recent study has shown that SARS-CoV-2 mediated neuroinflammation contributes to the pathophysiology of long COVID-19 symptoms, including neurological symptoms [59]. SARS-CoV-2 is a neurotropic virus that can infect the brain, which is supported by evidence from COVID-19 patients that indicates the presence of SARS-CoV-2 viral particles in both the CNS and brain [6]. Therefore, this suggests that SARS-CoV-2 can trigger neuroinflammation via the positive regulation of cytokine production and its ability to infect the brain, which might lead to the development of NDs.

Furthermore, our results indicated that 8.82% of the common ND terms belong to the functional group of regulation of protein ubiquitination and 7.84% of the terms belong to the group of response to unfolded protein. Protein misfolding and dysregulation of the ubiquitin proteasome system (UPS) is a common pathological mechanism of several NDs [60,61,62]. Hijacking and exploitation of the UPS is a common strategy employed by both DNA and RNA viruses which allows them to replicate and also evade immune responses [15,63,64]. Similarly to other viruses, SARS-CoV-2, can also manipulate the UPS during various stages of its life cycle, i.e through ubiquitin modifications of its viral proteins, which allows it to replicate and inhibit host immune responses [65]. As SARS-CoV-2 can modulate the cellular protein control systems it can possibly lead to their dysregulation, resulting in the build-up of misfolded proteins, causing neuroinflammation and neurotoxicity, that can possibly lead to the development of NDs.

#### 3.3.3. Neuropsychiatric Disorders

The integrated network of three neuropsychiatric disorders (Schizophrenia, Neurotic Disorder and BD) revealed 37 common biological processes (Figure 4E), that belong in 11 functional groups (Figure 4F). The results showed that 22.22% of the terms belong to the functional group of positive regulation of cytokine production. In addition, 6.67% of the common terms belong to the functional group of corticosteroid receptor signaling pathway. SARS-CoV-2 is characterized by aberrant inflammatory processes involving increased expression of pro-inflammatory cytokines that lead to neuroinflammation [12,66]. Pro-inflammatory cytokines and neuroinflammation are not only involved in the pathogenesis of neurological diseases, but also neuropsychiatric disorders and it has been suggested that modulating neuroinflammation might be a potential strategy for their treatment [67,68]. In addition, stress which is associated as a potential trigger in the development of neuropsychiatric disorders involves increased levels of glucocorticoids which result in a transient neuroinflammatory response [69]. Moreover, acute or chronic stress can lead to stress-induced neuroinflammatory priming affecting future neuroinflammatory and microglia responses to a subsequent immune challenge even after the stress exposure [69]. Therefore, as COVID-19 can modulate the corticosteroid receptor signaling pathway it might result in neuroinflammatory priming making the immune system hypersensitive to life stressor resulting in potentiated neuroinflammation, which might explain the long-term neuropsychiatric symptoms that persist after COVID-19 exposure.

In addition, 20.0% of the common GO biological processes belong to the functional group of catecholamine metabolic processes, suggesting that SARS-CoV-2 can modulate the production of dopamine, noradrenaline and adrenaline. Catecholamines are hormones that also act as neurotransmitters that are involved in brain functioning, including processes, such as learning, memory and mood [70]. The results also indicated that 15.56% of the terms belong to the functional group of learning and memory. This is consistent with current evidence that indicates that COVID-19 can cause in some individuals memory problems or brain fog, such as impairment in learning new information (memory encoding) that lasts months after infection [71,72]. Thus, impairments in learning and memory observed post-COVID-19 might result from altered production of catecholamines.

Catecholamines are also important for the regulation of emotions, with dopamine being considered as the “feel-good” hormone. Decreased levels of dopamine are associated with depression (neurotic disorder) [73], whereas increased levels of dopamine are associated with Schizophrenia and the manic phase of BD, causing the emergence of psychotic symptoms [74]. Therefore, the ability of SARS-CoV-2 to modulate the production of dopamine, either decrease or increase, can possibly lead to the emergence of neuropsychiatric effects, depending on its effect on dopamine levels. Dopamine is also the precursor for the production of adrenaline and noradrenaline; therefore, dysregulation of dopamine levels would also affect the production of other catecholamines.

Various viruses are known to exploit dopaminergic receptors for viral entry into cells [75,76]. For example, Japanese encephalitis virus (JEV), which is a neurotropic virus that causes acute encephalitis and chronic neuropsychiatric symptoms, exploits dopaminergic receptors to infect dopaminergic neurons and also modulates dopamine levels at the early stages of its infection [75]. In addition, JEV infection can cause parkinsonism in certain individuals and as indicated in a JEV-induced PD rat model its pathological effects could be significantly reversed using the antiparkinsonian drug L-DOPA, a dopamine precursor [77]. In another PD rat model induced by JEV, the infected rats had significant lower striatal dopamine levels and noradrenaline levels in the medulla oblongata and hypothalamus [78]. Patients with JEV-induced movement disorders also had lower cerebrospinal fluid (CSF) concentrations of catecholamines, dopamine and noradrenaline [79]. In addition, West Neil virus (WNV), which like JEV is also a member of the *Flaviviridae* family and a neurotropic virus, can also cause parkinsonism and in rare occasions neuropsychiatric sequels [80,81]. Similarly, to JEV, WNV also induces dopaminergic neuronal loss and exploits the dopaminergic system for its infectivity [82,83]. In an in vitro experimental model of WNV infection, it was shown that the antiparkinsonian drug Adamantine significantly inhibited WNV infection in cell lines [84]. Therefore, viruses exploit catecholamines to promote infectivity, which in turn can lead to neural dysfunction and the emergences of the associated diseases.

Catecholamines are also responsible for the body’s “fight or flight” stress response. Therefore, as SARS-CoV-2 can affect the production of catecholamines it suggests that it might be also able to alter the body’s “fight or flight” stress response system. Overactive or underactive “fight or flight” stress response has been found in young healthy individuals that where infected with SARS-CoV-2, which seems to contribute to “long-COVID-19” symptoms [85]. Chronic stress which is associated with overactive “fight or flight” stress response, is a significant factor in the emergence of neuropsychiatric disorders, such as depression and BD [86]. Therefore, this abnormal activity in the “fight or flight” stress response that may possibly be caused by SARS-CoV-2 might also exacerbate the symptoms of patients with existing neuropsychiatric disorders or promote their development when someone gets infected.

Moreover, catecholamines are also involved in the regulation of immune responses, as they can have immune enhancing or suppressive effects depending on their duration and levels [87]. For example, noradrenaline and adrenaline can modulate cytokine production, as they can inhibit the production of Th1 pro-inflammatory cytokines, thus favoring the shift to Th2 humoral immunity [88]. These stress-associated hormones can also regulate leukocyte trafficking [89], and leukocytes, like T cells, which in turn can synthesize and release catecholamines [90]. Therefore, a bidirectional crosstalk exists between the brain and the peripheral immune system as leukocyte can also synthesize and release catecholamines and at the same time produce cytokines that can target the brain and affect neurotransmitter production, thus participating in the neuro-immunomodulatory circuitry [91,92]. During infection, the release of immune mediators triggers the CNS to amplify immune responses mediated via hormonal and neural routes, and after infection, the CNS suppresses the immune system and restores homeostasis [93]. Therefore, the ability of SARS-CoV-2 to modulate the positive production of cytokines and affect the corticosteroid receptor signaling pathway, as well as the catecholamine metabolic processes, suggests that SARS-CoV-2 can affect the neuro-immunomodulatory circuitry. This can lead to apparent immune responses and brain functioning that can contribute to the emergence of neuropsychiatric disorders or “long- COVID” neuropsychiatric symptoms, as well as neurological diseases.

### 3.4. Disease-Associated Variant Genes/Proteins Interacting with SARS-CoV-2 Proteins

To identify genetic factors that might alter virus–host PPIs and thus influence virus–host dynamics leading to the emergence of edgetic perturbations during COVID-19 infection, we mapped on the COVID-19-host PPIs network the disease-associated genetic variants for the thirteen diseases/disorders, that were collected from *DisGeNET* database (Table 1). Our hypothesis is that the presence of these genetic variants in certain individuals, might influence the interaction between SARS-CoV-2 viral proteins and their human protein targets, leading to the emergence of edgetic perturbations in virus–host PPIs. Therefore, the presence of these disease-associated genetic variants in certain individuals might shift virus–host dynamics in disequilibrium disease states, thus predisposing an individual in the emergence of these associated diseases/disorders.

**Figure 5:**
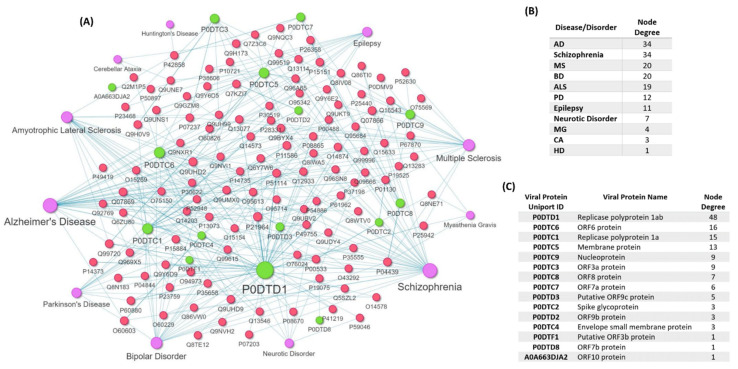
**Disease-Associated Variant Genes/Proteins Interacting with SARS-CoV-2 Proteins**. (**A**) Heterogenous subnetwork of the COVID-19-host PPI network indicating the human proteins that also have disease-associated variant genes/proteins for the eleven diseases/disorders (MS, AD, PD, ALS, HD, CA, MG, Schizophrenia, Neurotic Disorder, BD, Epilepsy) and are also targeted by the 15 viral proteins of SARS-CoV-2. The red nodes represent human disease-associated variant genes/proteins that are also targets of SARS-CoV-2 viral proteins, the purple nodes are diseases/disorders, and the green nodes are SARS-CoV-2 viral proteins. Node size is proportional to its degree, which indicates the number of connected edges to each node. (**B**) Node degree of each of the eleven diseases/diseases, indicating the number of human proteins with disease-associated variant genes that are targeted by SARS-CoV-2 proteins. (**C**) Node degree of each SARS-CoV-2 viral proteins, indicating the number of disease-associated variant genes that each viral protein targets.

The mapping revealed human proteins that are targeted by 15 SARS-CoV-2 viral proteins and also have genetic variants that are associated with the development of eleven diseases/disorders: MS, AD, PD, ALS, HD, CA, MG, Schizophrenia, Neurotic Disorder, BD, Epilepsy (Figure 5A). The number of disease-associated variant genes found on the COVID-19-host *PPIs* network for each of the eleven diseases/disorders is shown in Figure 5B. No variant genes associated with Prion Disease and GBS were found to be targeted by SARS-CoV-2 proteins. The analysis showed that AD and Schizophrenia have the highest number of 34 human proteins that are targeted by SARS-CoV-2 viral proteins that also have genetic variants associated with their development (Figure 5B).

In addition, the analysis of the subnetwork, indicates that Replicase polyprotein 1ab (P0DTD1) has the highest degree of interaction with variant genes as it targets 48 human proteins that have genetic variants associated with the development of these diseases/disorders (Figure 4C), whereas putative ORF3b protein (P0DTF1), ORF7b protein (P0DTD8), ORF10 protein (A0A663DJA2) viral proteins only target one human protein whose variant has been associated with disease development.

### 3.5. Cross-Reactivity Risk Potential of SARS-CoV-2 Epitopes Based on Similarity with Autoreactive Epitopes Found in Neurological Diseases

To investigate the potential of cross-reactivity, via molecular mimicry, of antigenic determinants of SARS-CoV-2 viral proteins that are recognized by the immune system with host “self” antigens, we performed pairwise sequence similarity with autoreactive epitopes found in MG, ALS, MS, AD, PD and GBS. Our hypothesis is that if SARS-CoV-2 epitopes have “sufficient” sequence similarity with known disease autoreactive epitopes then they can potentially cross-react with those proteins, thus leading to the emergence of the associated diseases. For this purpose, we isolated SARS-CoV-2 epitopes that contain matching linear motifs of five AAs or above with autoreactive epitopes found in neurological diseases. Our analysis led to the identification of 5-mer linear matching motifs between SARS-CoV-2 viral epitopes and autoreactive epitopes found in MG, AD, PD and MS. In addition, we identified matching 6-mer linear motifs with autoreactive epitopes found in MS. However, no linear motifs of six AAs or more were found between SARS-CoV-2 epitopes and autoreactive epitopes found in AD, PD, MG. Additionally, no linear motifs of seven AAs or more were found between SARS-CoV-2 epitopes and MS autoreactive epitopes. In addition, no linear motifs of five AAs or above were found in the consensus sequences between SARS-CoV-2 epitopes with GBS and ALS autoreactive epitopes.

#### 3.5.1. Matching 5-Mer Linear Motifs

The identified SARS-CoV-2 epitopes and autoreactive epitopes found in AD, MG and AD which contain matching 5-mer linear motifs in their consensus sequences are indicated in Table 4. More specifically, Replicase polyprotein 1ab epitopes contain the FFAQD, QGLVA and SSAKS 5-mer linear motifs that are also found in autoreactive epitopes against APP, acetylcholine receptor subunit gamma and MAPT, respectively. The autoreactive epitopes were derived from AD, MG and PD data, respectively. Thus, the identified Replicase polyprotein 1ab epitopes can possibly cross-react with these human proteins leading to autoreactivity and the development of the associated NDs. In addition, Replicase polyprotein 1a epitopes contain the SVLLS and the SSAKS 5-mer linear motifs that are also present in autoreactive epitopes against acetylcholine receptor subunit alpha and MAPT, respectively, that are found in MG and PD autoreactive epitopes, respectively.

The SARS-CoV-2 epitopes that contain matching 5-mer motifs with autoreactive epitopes found in AD, PD and MG are from Replicase polyprotein 1a and 1ab viral proteins, which are encoded by ORF1a and ORF1ab, respectively. They are non-structural proteins that are only expressed in infected cells and unlike SARS-CoV-2 structural proteins they do not form part of the virion particle [94]. These polyproteins are proteolytically cleaved to produce non-structural proteins (Nsp1-16) which includes various transcriptional factors and enzymes which are important for viral replication [94]. The Nsp proteins are also involved in immune evasion by suppressing antiviral and innate immunity host responses [95,96,97].

In addition, our analysis revealed 1085 matching 5-mer linear motifs in the consensus sequences of SARS-CoV-2 epitopes with autoreactive epitopes found in MS, which are indicated in Supplementary Materials. Due to the large number of matching pairs, in Table 5, we summarize the 121 human proteins that are targeted by the MS-associated autoreactive epitopes and the matching eight viral proteins whose epitopes contain the identified matching 5-mer linear motifs. As indicated in Table 5, Replicase polyprotein 1a of SARS-CoV-2 has the higher number of matching 5-mer motifs with human proteins associated with MS autoreactive epitopes, thus potentially having the highest diversity of autoreactivity with human proteins. These includes autoreactive epitopes against myelin components, myelin basic protein (MBP) and myelin proteolipid protein (PLP). In addition, it includes autoreactive epitopes against immune components, such as Interleukin-12 receptor subunit beta-1 and HLA class I histocompatibility antigens, which play an important role in MS pathogenesis. Moreover, the results include autoreactive epitopes against GFAP and NfL polypeptide which are associated with MS disease severity [98,99]. A recent study has shown the presence of elevated levels of serum neurodegenerative biomarkers in hospitalized COVID-19 patients with levels similar to those observed in AD dementia [100]. In addition, the serum levels of total tau, phosphorylated tau-181, GFAP and NfL chain were significantly elevated in individuals that died in the hospital due to COVID-19 [100]. Thus, the presence of these proteins in the blood due to COVID-19 and the potential of cross-reactivity of the identified SARS-CoV-2 epitopes increases the risk for neurodegeneration.

Our analysis results also indicated matching 5-mer linear motifs of SARS-CoV-2 epitopes with MS autoreactive epitopes that target the Olfactory receptors 10A4 and 4N2. Loss of smell is a common symptom found during COVID-19 and hyposmia or anosmia can also appear during or after COVID-19 [101,102]. Moreover, our results also include matching 5-mer motifs with autoreactive epitopes against Melatonin receptor type 1A involved in sleep regulation, Glutamate decarboxylase 2 an enzyme involved in the catalysis of glutamate to Gamma-aminobutyric acid receptor subunit alpha-3, and against Sperm-egg fusion protein LLCFC1.

#### 3.5.2. Matching 6-Mer Linear Motifs

Our analysis also indicated the presence of matching 6-mer linear motifs between SARS-CoV-2 epitopes and autoreactive epitopes found in MS. Table 6 summarizes the 13 human proteins that are targeted by the MS-associated autoreactive epitopes and the seven SARS-CoV-2 viral proteins that contain the matching 6-mer linear motifs. The complete list of the matching 6-mer linear motifs between SARS-CoV-2 epitopes and the MS-associated autoreactive epitopes can be found in Supplementary Materials. As indicated in Table 5 and Table 6, both 5-mer and 6-mer matching linear motifs were found between SARS-CoV-2 epitopes and MS-associated autoreactive epitopes that target the 60 kDa heat shock protein, which is a mitochondrial chaperone involved in mitochondrial protein import. Dysfunction of protein import into mitochondria is associated with various diseases including NDs, such as AD and PD [103]. Viruses are known to affect mitochondria functions in order to promote viral replication and evade host antiviral defenses, as mitochondria play a central role in host antiviral mechanisms, such as through the production of ROS or mitochondria-mediated apoptosis of viral infected cells [104,105,106]. Similarly, to other viruses, evidence indicates that SARS-CoV-2 can also hijack mitochondria functions in infected peripheral blood mononuclear cells leading to mitochondria dysfunction and metabolic alternations [107]. Both 5-mer and 6-mer matching linear motifs were also found between SARS-CoV-2 epitopes and autoreactive epitopes against immune component C-X-C motif chemokine 2 and the Actin, aortic smooth muscle protein which is found in smooth muscle cells (Table 5 and Table 6).

## 4. Discussion

Pathogenic organisms, including viruses, have been associated as risk factors for the development of several diseases, particularly autoimmune-mediated diseases. The introduction of this new virus, SARS-CoV-2, into the repertoire of pathogens that can interact with the human host has introduced the need to investigate and identify potential diseases that could emerge due to this interaction. Understanding these virus–host interactions could also possibly provide greater insight into the pathogenic mechanisms that lead to COVID-19 disease severity and the emergence of post-COVID-19 symptoms. Most importantly, being able to predict diseases that can emerge due SARS-CoV-2 infection can possibly enable to identify preventive pharmacotherapies that could slow down or even prevent the development of these diseases.

In this study, we have developed a bioinformatics-based methodology with the aim to computationally identify pathological mechanisms by which SARS-CoV-2 infection can lead to the development of neurological diseases (ALS, Prion Disease, HD, PD, MS, CA, MG, GBS, AD, Epilepsy) and neuropsychiatric disorders (ND, BD, Schizophrenia). Viruses interact with the human host via virus–host PPIs which allows them to manipulate host processes for their own advantage. These viral mediated effects can result into the dysregulation of host biological processes leading to the development of various diseases. In the first part of our investigation, we explored whether SARS-CoV-2, via virus–host PPIs, can lead to the dysregulation of biological processes associated with the pathogenesis of the thirteen diseases/disorders. In addition, we investigated whether SARS-CoV-2 epitopes can possibly have cross-reactivity with host self-antigens, via molecular mimicry, by comparing them with autoreactive epitopes found in neurological diseases. Moreover, we identified disease-associated susceptibility genes that might alter virus–host PPIs during COVID-19 infection and lead to the emergence of edgetic perturbations, thus resulting in alter virus–host disequilibrium disease states.

Our methodology first involved the reconstruction of thirteen integrated COVID-19-host-disease PPI networks. This allowed to identify overlapping GO biological processes (COVID-19 ∩ Disease) and functional groups of biological processes associated with these diseases/disorders that can be possibly modulated by COVID-19 (see Figure 3 and Supplementary Materials). Thus, the identified processes might represent possible pathological mechanisms by which COVID-19 might result in their development/progression.

Our results also suggest that there might be a higher risk for the development of NDs after SARS-CoV-2 infection via the mechanism of virus–host PPIs than the development of neuropsychiatric disorders. It is important to note that HD is a hereditary disease and although SARS-CoV-2 cannot lead to its development, it might cause early age of symptom onset. Early age of symptom onset in HD, due to viral infection has been observed in HD-mutant carriers of at least 36 CAG repeats that were infected with human immunodeficiency virus [108].

In addition, our results indicated that AD has the highest number of overlapping GO biological processes (COVID-19 ∩ Disease) that can be modulated by SARS-CoV-2 through virus–host PPIs, whereas Epilepsy has the lowest number of overlapping processes, Figure 3A. Cognitive symptoms, such as memory impairment and brain fog, are commonly reported after COVID-19 and memory problems are also a major symptom of AD [71,72,109]. A recent case study has shown that a patient that had cognitive symptoms after COVID-19 had increased CSF biomarkers associated with AD [110]. In addition, elevated levels of serum neurodegenerative biomarkers similarly to those observed in AD have been found in hospitalized COVID-19 patients [100]. In combination with our results, this suggests that SARS-CoV-2 through virus–host PPIs can possibly lead to the dysregulation of biological processes associated with AD, resulting in post-COVID-19 cognitive symptoms that arise from AD-like pathology. Viral-mediated diseases can be acute or develop into chronic diseases in certain cases, thus further investigation is warranted to determine whether this AD-like pathologies post-COVID-19 represent acute effects or an early stage of AD.

Moreover, our results suggest that SARS-CoV-2 can possibly lead to the development/progression of NDs (ALS, AD, PD, HD, Prion Disease and CA) through several of the known common pathological mechanisms found in NDs (Figure 4D). This includes protein misfolding and aggregation [111], oxidative stress due to excessive production of ROS [112] and neuroinflammation associated with increased immune system activation and cytokine production within the CNS [113].

Neuropsychiatric symptoms are common in COVID-19 patients and might persist long after infection, although their severity decreases after COVID-19 recovery [114]. Our analysis revealed several common pathological mechanisms by which SARS-CoV-2 can possibly lead to the development of neuropsychiatric disorders (Schizophrenia, Neurotic Disorder and BD) including modulation of immune system processes, catecholamine production and the stress response system. This suggests that SARS-CoV-2 can possibly affect the neuro-immunomodulatory circuitry. Thus, through this mechanism, it may contribute to the exacerbation of symptoms in existing neuropsychiatric patients or promote their development.

Our analysis also indicated that a common pathological mechanism by which SARS-CoV-2 can possibly lead to the development/progression of the three neurological diseases of autoimmune origin (MS, MG and GBS) is through the positive modulation of cytokine production (Figure 4A). The pathogenesis of autoimmune diseases, is associated with increased production of pro-inflammatory cytokines [53,54] and cytokine storms can also be found in some autoimmune diseases, such MS [115]. SARS-CoV-2 can also infect macrophages allowing it to produce high levels of cytokines that are thought to promote tissue inflammation and cytokine storm syndrome [116,117]. Uncontrolled activation of infected SARS-CoV-2 macrophages can lead to macrophage activation syndrome (MAS) associated with acute respiratory distress and death [118,119,120]. MAS in rare cases can also occur in some autoimmune diseases [121,122]. Therefore, SARS-CoV-2 via virus–host PPIs might modulate the positive regulation of cytokine production in infected immune cells promoting inflammation and cytokine storm syndrome, which might lead to the development of autoimmune-mediated diseases in certain individuals.

However, although COVID-19 might possibly confer increased risk for the development of neurological diseases and neuropsychiatric disorders through dysregulation of biological processes via virus–host PPI, other risk factors (genetic or environmental) could also contribute to determining the outcome and extent of this dysregulation. The combinatorial actions between SARS-CoV-2 infection and these additional risk factors would also possibly determine which disease phenotype might emerge. Therefore, the presence or absence of these additional risk factors during SARS-CoV-2 infection could possibly determine the potential risk level that someone has in developing these diseases/disorders. For example, sleep deprivation causes increased production of beta-amyloid protein which is associated with the development of AD, thus lack of sleep may increase dementia risk [123]. Sleep deprivation also weakens the immune system leading to increased susceptibility to viral infections [124]. Therefore, the combinatorial effects between lack of sleep and SARS-CoV-2 infection may confer higher risk in developing NDs rather than either factor alone.

The next part of our investigation involved the identification of potential genetic factors which in combination with SARS-CoV-2 infection could possibly affect virus–host PPIs and lead to edgetic perturbations that would alter COVID-19 pathogenicity and disease outcome, predisposing an individual in the emergence of the thirteen diseases/disorders. Our results showed that AD and Schizophrenia have the highest number of human proteins associated genetic variants involved in their development that are also targeted by SARS-CoV-2 viral proteins (Figure 5B). Thus, the presence of these genetic variants in certain individuals might result in edgetic perturbations of virus–host PPIs shifting virus–host dynamic in disease disequilibrium states.

Viruses can also lead the emergence of diseases, indirectly through autoreactivity. To investigate whether SARS-CoV-2 epitopes that are recognized by immunological memory cells can cross-react with host “self” antigens, we performed sequence similarity between linear SARS-CoV-2 epitopes and linear autoreactive epitopes found in neurological diseases: MG, ALS, MS, AD, PD and GBS. Other papers have already investigated the molecular similarity between SARS-CoV-2 epitopes and human proteins in various tissues [125]. For example, [125] examined the autoreactivity potential of SARS-CoV-2 immunogenic epitopes by identifying parts that are homologous with sequences of human proteins. However, proteins contain immunogenic regions, which are more easily recognized by the immune system. Thus, matching a SARS-CoV-2 epitope with parts of a human protein does not necessarily mean that the matching region is immunogenic as it might be located in an inaccessible surface area of the protein. To overcome this possible limitation, in our study we choose to determine the potential risk of cross-reactivity of SARS-CoV-2 epitopes by comparing them with known autoreactive epitopes, in order to account for the immunogenicity potential of human proteins and their immunogenic regions. Thus, the identified matching motifs are located in AA regions which are known to be recognized by immunological memory cells, and thus can trigger autoreactivity. To identify SARS-CoV-2 epitopes that can possibly cross-react with human proteins, we isolated SARS-CoV-2 epitopes that contain linear motifs of five AAs or above that also present in autoreactive epitopes associated with neurological diseases. Therefore, our analysis was only designed to capture matching linear motifs and not discontinued or conformational motifs.

Our methodology led to the identification of SARS-CoV-2 epitopes that have 5-mer and/or 6-mer linear motifs within their sequences that are also found in autoreactive epitopes associated with PD, AD, MG and MS (see Table 4, Table 5 and Table 6 and Supplementary Materials). These matching autoreactive epitopes target various host components such as neuronal and immune components, including proteins involved in neurotransmission, neuroaxonal integrity and mitochondria function. Thus, our results suggest that certain individuals that will form immunological memory cells that recognize SARS-CoV-2 epitopes that contain these specific 5-mer and/or 6-mer linear motifs, will have increased potential risk of cross-reactivity with human proteins associated with neurodegeneration. However, some of these proteins are also associated with other diseases, including neuropsychiatric disorders, autoimmune and cerebrovascular diseases, thus the potential risk of SARS-CoV-2 autoreactivity can possibly lead to a range of pathologies. Further studies are required to examine this potential risk with the aim to identify possible preventive measures.

However, our study is not without limitations. One important limitation of our study, is that non-statistically significant GO biological processes could also be important, as SARS-CoV-2 might exert disease effects through them [23]. In addition, more studied genes might be associated with more GO biological processes than less studied genes. For example, AD is a more studied disease than Epilepsy, hence AD-associated genes will be more studied than Epilepsy-related genes, thus genes that relate to AD might produce more associated GO biological processes than Epilepsy-related genes. This limitation of enrichment analysis might have impacted our results, despite our effort to try to minimize the impact of differences in data availability between diseases (more studied diseases vs. less studied diseases) by using the same number of diseases-associated genes for each disease/disorder in our analysis.

Regarding the exploration of the cross-reactivity risk potential of SARS-CoV-2 epitopes, our methodology was designed to highlight matching linear motifs between SARS-CoV-2 epitopes and autoreactive epitopes found in neurological diseases, and exclude discontinued motifs in this study. However, cross-reactivity can also occur between matching discontinued or conformational motifs, therefore additional SARS-CoV-2 epitopes could also possibly cross-react with human proteins. Although our methodology did capture and led to the identification of matching discontinued motifs of more than 5AA between SARS-CoV-2 epitopes and autoreactive epitopes found in NDs (unpublished data), these results can be presented in future work. It is also important to experimentally validate the cross-reactivity potential of the identified SARS-CoV-2 epitopes with their matching human proteins, by using immunoassay methods.

Moreover, in this study we choose to focus on a specific list of 10 neurological diseases and 3 neuropsychiatric disorders. The diseases/disorders examined in this study were selected based on the hypothesis that diseases/disorders whose development and/or progression has been associated with viral infections have a higher probability to be triggered by a new emerging virus, in this case SARS-CoV-2, than other diseases, as viruses share several common pathogenic mechanisms. However, our selection list is not exhaustive as other neurological diseases or even other types of diseases, particularly autoimmune diseases, which have a strong association with viral infections can also potentially be triggered by COVID-19. Nonetheless, these diseases are beyond the scope of this paper and may be addressed in future works using the presented computational methodology.

## 5. Conclusions

In this study, we used an integrative bioinformatics exploratory approach to investigate how SARS-CoV-2 infection can affect existing patients with neurological diseases or neuropsychiatric disorders or whether it can lead to their development. We examined two main pathological mechanisms by which viruses are known to cause disease: (i) virus–host PPIs and (ii) autoreactivity.

Our analysis results indicated that NDs (ALS, Prion Disease, HD, AD, PD, MG, MS, CA) have a higher number of GO biological processes that can be modulated by SARS-CoV-2, via virus–host PPIs, than neuropsychiatric disorders (Neurotic Disorder, Bipolar Disorder, Schizophrenia). In addition, our results highlighted the positive regulation of cytokine production as a common pathological mechanism by COVID-19 that can possibly lead in the development of both neurological diseases and neuropsychiatric disorders.

Moreover, our analysis indicated that modulation of neuro-immune crosstalk by SARS-CoV-2 might facilitate the development of neuropsychiatric disorders or exacerbate symptoms in existing patients. Based on our results, we suggest that therapeutic strategies that aim to treat COVID-19 mediated neuropsychiatric symptoms should account for neuro-immune crosstalk, and hence a combination of immunomodulatory and neuromodulatory therapies might be more appropriate rather than only the use of neuropsychiatric drugs.

Furthermore, the results of our analysis indicated the presence of several matching 5-mer and/or 6-mer linear motifs between SARS-CoV-2 epitopes and autoreactive epitopes found in AD, PD, MG and MS. The results include autoreactive epitopes that recognize APP, MAPT, acetylcholine receptors, GFAP, NfL polypeptide and major myelin proteins.

Further studies are required to examine this potential risk with the aim to identify possible preventive measures. In addition, the impact of other indirect pathogenic mechanisms mediated by COVID-19 that might increase the risk for the development of these diseases/disorders should also be investigated. COVID-19 infection was shown to cause the reactivation of latent viruses, including Epstein–Barr virus and herpes simplex virus 1, as well as gut dysbiosis, which are all risk factors for the development of both NDs and neuropsychiatric disorders [126,127,128,129,130,131,132,133,134,135].

## Figures and Tables

**Table 1:** Disease-associated variants collected for the included neurological diseases and neuropsychiatric disorders.

Disease/Disorder	Number of Disease-Associated Variants from *DisGeNET* Database with Available Gene Name
**ALS**	299
**CA**	87
**PD**	460
**AD**	737
**MG**	73
**HD**	NA
**MS**	536
**Prion Disease**	26
**GBS**	11
**BD**	402
**Epilepsy**	208
**Neurotic Disorder**	191
**Schizophrenia**	1179

**Table 2:** Number of linear epitopes collected that recognize SARS-CoV-2 immunogenic viral proteins.

Viral Proteins Name	Viral Proteins Uniprot ID	Number of Linear Epitopes
**ORF7b protein**	P0DTD8	3
**ORF10 protein**	A0A663DJA2	19
**ORF6 protein**	P0DTC6	22
**Envelope small membrane protein**	P0DTC4	24
**ORF7a protein**	P0DTC7	49
**ORF8 protein**	P0DTC8	87
**ORF3a protein**	P0DTC3	171
**Membrane protein**	P0DTC5	218
**Nucleoprotein**	P0DTC9	512
**Spike glycoprotein**	P0DTC2	1284
**Replicase polyprotein 1a**	P0DTC1	1292
**Replicase polyprotein 1ab**	P0DTD1	1315
	**Total**	**4996**

**Table 3:** Number of linear autoreactive epitopes collected for the 6 neurological diseases and the number of human proteins that they target.

Neurological Disease	Number of Autoreactive Linear Epitopes	Number of Human Proteins Associated with Autoreactivity
**MG**	284	10
**GBS**	11	5
**MS**	3512	1289
**AD**	62	6
**PD**	66	4
**ALS**	7	3

**Table 4:** Matching 5-mer linear motifs between SARS-CoV-2 epitopes and autoreactive epitopes found in AD, MG and AD.

Disease	Disease-Associated Autoreactive Epitopes	SARS-CoV-2 Epitopes	Consensus Sequence
**AD**	P05067-52 (APP) DAEFRHDSGYEVHHQKLVFFAQDVGSNKGAIIGLMVGGVV	P0DTD1-830 (Replicase polyprotein 1ab) ELKHFFFAQDGNAAI	K??FFAQD
**AD**	P05067-52 (APP) DAEFRHDSGYEVHHQKLVFFAQDVGSNKGAIIGLMVGGVV	P0DTD1-858 (Replicase polyprotein 1ab) FFFAQDGNAAISDYD	FFAQD
**AD**	P05067-52 (APP) DAEFRHDSGYEVHHQKLVFFAQDVGSNKGAIIGLMVGGVV	P0DTD1-1178 (Replicase polyprotein 1ab) SVELKHFFFAQDGNA	K??FFAQD
**MG**	P02708-64 (Acetylcholine receptor subunit alpha) LPTDSGEKMTLSISVLLSLTV	P0DTC1-21 (Replicase polyprotein 1a) KMVSLLSVLLSMQGA	KM????SVLLS?
**MG**	P07510-24 (Acetylcholine receptor subunit gamma) WQRQGLVAAALEKLEKGPEL	P0DTD1-685 (Replicase polyprotein 1ab) SQGLVASIKNFKSV	QGLVA?
**PD**	P10636-22 (MAPT) PKSPSSAKSRLQTAPV	P0DTD1-288 (Replicase polyprotein 1ab) EESSAKSASVY	SSAKS
**PD**	P10636-22 (MAPT) PKSPSSAKSRLQTAPV	P0DTD1-410 (Replicase polyprotein 1ab) SSAKSASVY	SSAKS
**PD**	P10636-22 (MAPT) PKSPSSAKSRLQTAPV	P0DTC1-64 (Replicase polyprotein 1a) CEESSAKSASVYYSQ	SSAKS
**PD**	P10636-22 (MAPT) PKSPSSAKSRLQTAPV	P0DTC1-330 (Replicase polyprotein 1a) EESSAKSASVYYSQL	SSAKS
**PD**	P10636-22 (MAPT) PKSPSSAKSRLQTAPV	P0DTC1-1178 (Replicase polyprotein 1a) VFDGKSKCEESSAKS	SSAKS

**Table 5:** Matching 5-mer linear motifs found in epitopes against SARS-CoV-2 viral proteins that are also present in autoreactive epitopes against self-protein found in MS.

SARS-CoV-2 Viral Proteins Names	Uniprot ID of Human Protein Targets of MS-Associated Autoreactive Epitopes and Matching 5-Mer Motifs Found between SARS-CoV-2 Epitopes and MS Autoreactive Epitopes
**P0DTC1** **(Replicase polyprotein 1a)**	J3QL64-[YLATA], P09543-[GKPVP], P02686-[YLATA], P10809-[IPKEE], P37837-[NYYKK], P60201-[ATLVS], P60201-[FFFLY], P04271-[NNELS], Q9H209-[VATVQ], P42701-[STVLS], P01042-[AVDAA], Q13571-[SKTPE], P11586-[QVNGL], P62861-[ARAGK], P62736-[EKSYE], P14921-[FITES], P04150-[EVVEN], Q06323-[DIILK], P05783-[LGSAL], P62316-[EEEEF], Q53YP1-[KDYLA], P08575-[KALRK], F1MPL6-[KDYLA], P05109-[LNSII], P29401-[YFDKA], Q05329-[QTTLK], A0A0D9SGF6-[RQGFV], P09972-[EASFN], A0A1W2PRT3-[DGEVI], P29401-[AEAEL], P08779-[AGALN], P08238-[SAFVE], Q13813-[KAGQK], O75508-[LALCA], P07196-[EEVLS], P09972-[VTALR], A0A0C4DGB6-[TFLKK], Q00653-[DFKLN], O43318-[DIAST], Q7L8L6-[TSSSK], Q15052-[ITGNT], P25440-[KKGAK], Q6ZV29-[KKPAS], Q8WXI7-[STVLS], P30153-[NVAKS], C9JXB8-[SFSGY], P18669-[GKAKK], O75486-[LLFLM], Q8MH48-[KDYLA], Q8NAP8-[VTDTP], Q9HCE3-[KLKAL], Q92688-[EEEEF], P0C7M7-[VSSPD], Q6V0I7-[GTGTI], P11487-[GIVAI], Q9Y6P5-[SEDAQ], A0A0C4DGB6-[LPSLA], P48039-[VLQVR], Q9BYE9-[LLVLV], P18858-[GKAKK], Q8NGD1-[DIQLL], P62854-[FDAYV], O95425-[LPTGV], Q5JNZ5-[FDAYV], Q9HC29-[LAKNV], Q96L11-[PILLL], Q9Y657-[RGMVL]
**P0DTC2** **(Spike glycoprotein)**	P0DTU3-[FLLKY], O95674-[LNRAL], H7C2W9-[VTYVP], Q9NQ90-[GETGK], Q9BZR6-[LQELG], I3L0Y5-[GAGAA], O43318-[TNTSN], Q75MD7-[GAGAA], Q9ULK6-[GAGAA], P15880-[SYLTP], P05164-[DQLTP], P05164-[IVRFP], O75486-[EDLLF], Q8N446-[CCSCG], Q9NW61-[SFIED], Q6V0I7-[LTGTG], Q9NWB7-[KSNLK], Q9NTJ3-[VEAEV], Q9NTJ3-[KVEAE], Q13507-[VVLSF], Q15397-[AQEKN], Q5JNZ5-[VKLHY]
**P0DTC3 (ORF3a protein)**	Q6H3 × 3-[RATAT], Q9BYE9-[GVALL], P19875-[LLVAA]
**P0DTC4 (Envelope small membrane protein)**	P69905-[LVTLA]
**P0DTC5 (Membrane protein)**	P37108-[LLESE], P09972-[VLAAV], P09972-[LAAVY], Q5RI18-[KLLEQ], Q16695-[ARKSA]
**P0DTC8 (ORF8 protein)**	Q16695-[ARKSA], K7ERT8-[RKSAP], Q13630-[VGARK]
**P0DTC9 (Nucleoprotein)**	P10321-[ALLLL], Q13875-[SRGGS], A0A075B6H9-[PSASA], P05783-[SSSRS], P57073-[APSAS], P57073-[PSASA], P11161-[APSAS], P34903-[APSAS], Q8N729-[LALLL], Q8N729-[ALLLL], O95450-[ALLLL], O75486-[KDKKK], Q96GD3-[ALLLL], P82914-[YYRRA], Q6UX71-[VTQAF], P19875-[ALLLL], Q8IX21-[SSRSS]
**P0DTD1 (Replicase polyprotein 1ab)**	J3QL64-[YLATA], Q53Z42-[APRTL], P09543-[GKPVP], P02686-[YLATA], P37837-[NYYKK], A0A0A6YYK6-[SRQRL], Q8IUQ4-[LPTGT], Q6UWS5-[TVAGV], Q01082-[SSVEL], Q16663-[SVAAL], P14174-[VPRAS], P11586-[QVNGL], P14921-[FITES], P04150-[EVVEN], Q06323-[DIILK], P62316-[EEEEF], P47914-[TQAPT], P62937-[TVFFD], P06733-[ADLYK], Q05329-[LKYAI], Q05329-[QTTLK], P02489-[DDFVE], P09972-[EASFN], A0A1W2PRT3-[DGEVI], P08238-[LFENK], P29401-[AEAEL], P02511-[IRRPF], P10809-[KGVIT], P23297-[VAALT], Q86V81-[LDAYN], Q7L8L6-[TSSSK],Q6ZV29-[KKPAS], P30153-[RFNVA], P47914-[KPRSQ], C9JXB8-[SFSGY], P23528-[KEILV], P62851-[LLSKG], Q9UHN6-[YTFEK], Q96PV4-[KAVFI], P07355-[RDLYD], Q7Z4T9-[VYSFL], Q7Z4T9-[KYFVK],Q8NAP8-[VTDTP], Q92688-[EEEEF], P0C7M7-[VSSPD], P32246-[RARTV], P11487-[GIVAI], Q9Y6P5-[SEDAQ], Q8WXH0-[DTLKE], A0A0C4DGB6-[LPSLA], P62854-[FDAYV], O95425-[LPTGV], Q5JNZ5-[FDAYV], Q9HC29-[LAKNV], Q96L11-[PILLL]

**Table 6:** Matching 6-mer linear motifs found in epitopes against SARS-CoV-2 viral proteins that are also present in MS-associated autoreactive epitopes that target “self” proteins.

Autoreactive Epitope Human Protein Target	Viral Epitope Protein Target	6-Mer Motif
P10809 (60 kDa heat shock protein, mitochondrial)	P0DTD1 (Replicase polyprotein 1ab)	EIPKEE
P10809 (60 kDa heat shock protein, mitochondrial)	P0DTC1 (Replicase polyprotein 1a)	EIPKEE
P0DP02 (Immunoglobulin heavy variable 3-30-3)	P0DTD1 (Replicase polyprotein 1ab)	YYRARA
P0DP02 (Immunoglobulin heavy variable 3-30-3)	P0DTC1 (Replicase polyprotein 1a)	YYRARA
P62861 (40S ribosomal protein S30)	P0DTC1 (Replicase polyprotein 1a)	LARAGK
P62736 (Actin, aortic smooth muscle)	P0DTC1 (Replicase polyprotein 1a)	EKSYEL
P02489 (Alpha-crystallin A chain)	P0DTD1 (Replicase polyprotein 1ab)	DDFVEI
P09972 (Fructose-bisphosphate aldolase C)	P0DTC5 (Membrane protein (M))	VLAAVY
Q16695 (Histone H3.1t)	P0DTC8 (ORF8 protein)	ARKSAP
P25440 (Bromodomain-containing protein 2)	P0DTD1 (Replicase polyprotein 1ab)	KKGAKL
P25440 (Bromodomain-containing protein 2)	P0DTC1 (Replicase polyprotein 1a)	KKGAKL
P57073 (Transcription factor SOX-8)	P0DTC9 (Nucleoprotein (N))	APSASA
Q8N729 (Neuropeptide W)	P0DTC9 (Nucleoprotein (N))	LALLLL
Q9NWB7 (Intraflagellar transport protein 57 homolog)	P0DTC2 (Spike glycoprotein (S))	RKSNLK
Q9NTJ3 (Structural maintenance of chromosomes protein 4)	P0DTC2 (Spike glycoprotein (S))	KVEAEV
P19875 (C-X-C motif chemokine 2)	P0DTC3 (ORF3a protein)	LLLVAA

## Data Availability

Publicly available datasets were analyzed in this study. This data can be found here: IEDB (https://www.iedb.org/) (accesed on 28 November 2021), PHISTO (https://phisto.org/) (accessed on 16 September 2021), VirHostNet 3.0 (https://virhostnet.prabi.fr/) (accessed on 16 September 2021), DISEASES (https://diseases.jensenlab.org/Search) (accessed on 26 August 2021).

## Supplementary Materials

The following supporting information can be downloaded at: https://www.mdpi.com/article/10.3390/v14102270/s1, Figure S1: Functional enrichement analysis results of the overlapping GO biological processes between Prion Disease and COVID-19, indicating the functional groups and the percentage of terms that belong to each group; Figure S2: Functional enrichement analysis results of the overalpping GO biological processes between Bipolar Disorder and COVID-19, indicating the functional groups and the percentage of terms that belong to each group. Figure S3: Functional enrichement analysis results of the overlapping GO biological processes between Cerebellar Ataxia and COVID-19, indicating the functional groups and the percentage of terms that belong to each group; Figure S4: Functional enrichement analysis results of the overlapping GO biological processes between Myasthenia Gravis and COVID-19, indicating the functional groups and the percentage of terms that belong to each group; Figure S5: Functional enrichement analysis results of the overlapping GO biological processes between Multiple Sclerosis and COVID-19, indicating the functional groups and the percentage of terms that belong to each group; Figure S6: Functional enrichement analysis results of the overlapping GO biological processes between Schizophrenia Disorder and COVID-19, indicating the functional groups and the percentage of terms that belong to each group; Figure S7: Functional enrichement analysis results of the overlapping GO biological processes between Epilepsy and COVID-19, indicating the functional groups and the percentage of terms that belong to each group; Figure S8: Functional enrichement analysis results of the overlapping GO biological processes between Neurotic Disorder and COVID-19, indicating the functional groups and the percentage of terms that belong to each group; Figure S9: Functional enrichement analysis results of the overlapping GO biological processes between Guillain-Barre Syndrome and COVID-19, indicating the functional groups and the percentage of terms that belong to each group; Figure S10: Functional enrichement analysis results of the overlapping GO biological processes between Parkinson’s Disease and COVID-19, indicating the functional groups and the percentage of terms that belong to each group; Figure S11: Functional enrichement analysis results of the overlapping GO biological processes between Amyotrophic Lateral Sclerosis and COVID-19, indicating the functional groups and the percentage of terms that belong to each group; Figure S12: Functional enrichement analysis results of the overlapping GO biological processes between Huntington’s Disease and COVID-19, indicating the functional groups and the percentage of terms that belong to each group; Figure S13: Functional enrichement analysis results of the overlapping GO biological processes between Alzheimer’s Disease and COVID-19, indicating the functional groups and the percentage of terms that belong to each group; Table S1: Matching 5-mer linear motifs found in the consensus sequences between SARS-CoV-2 epitopes and autoreactive epitopes found in MS, with the 5-mer motif highlighted in their epitope sequences.
