# Supplementary material for: Systems Bioinformatics Reveals Possible Relationship between COVID-19 and the Development of Neurological Diseases and Neuropsychiatric Disorders
Source: Viruses. 2022 Oct 16;14(10):2270. doi: 10.3390/v14102270 (PMC9611753; doi:10.3390/v14102270)
Supplement: Supplementary file 1 [file viruses-14-02270-s001.zip › viruses-1943446-supplementary.pdf]

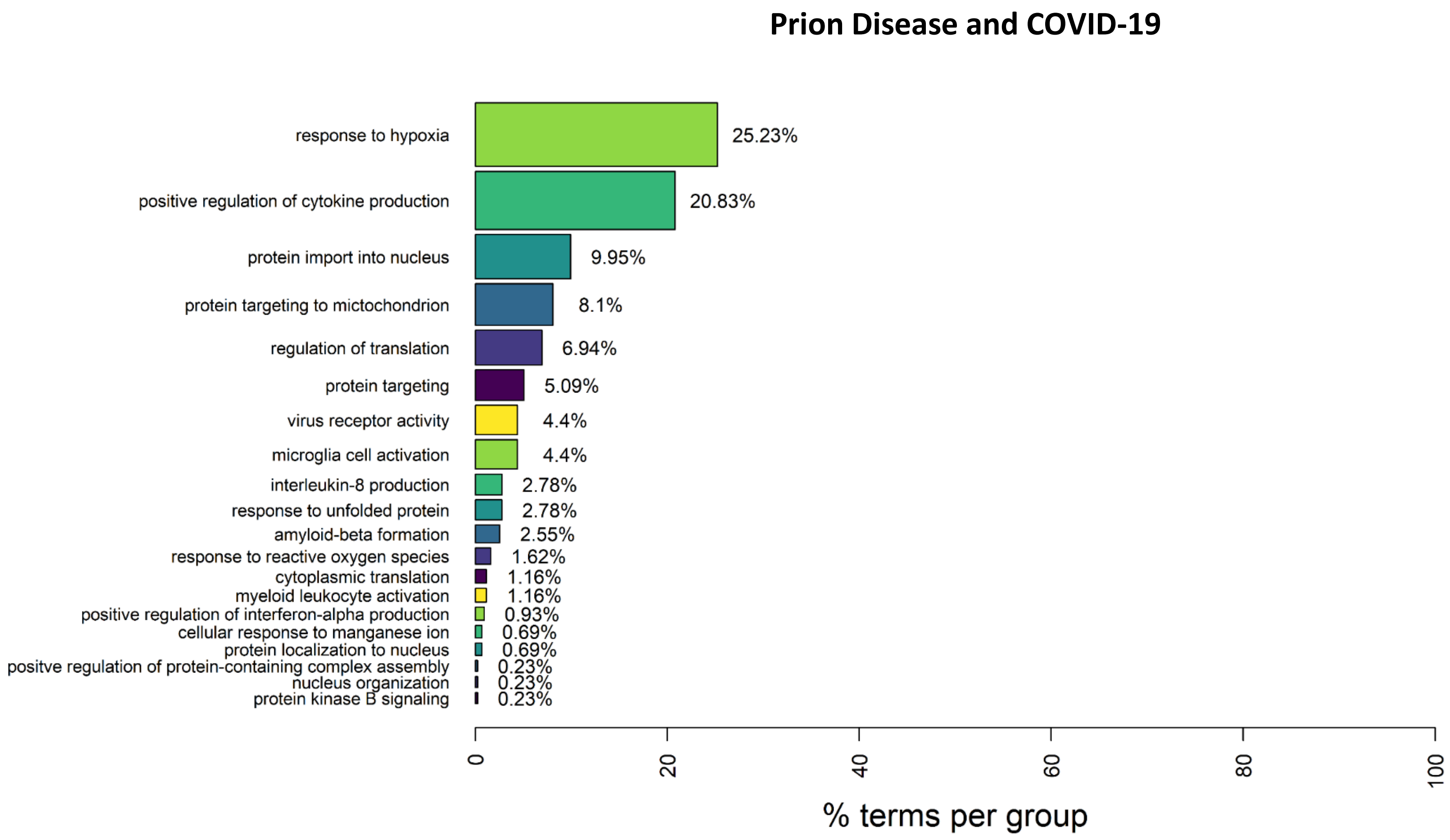

Supplementary Figure S1: Functional enrichement analysis results of the overlapping GO biological processes between Prion Disease and COVID-19, indicating the functional groups and the percentage of terms that belong to each group.

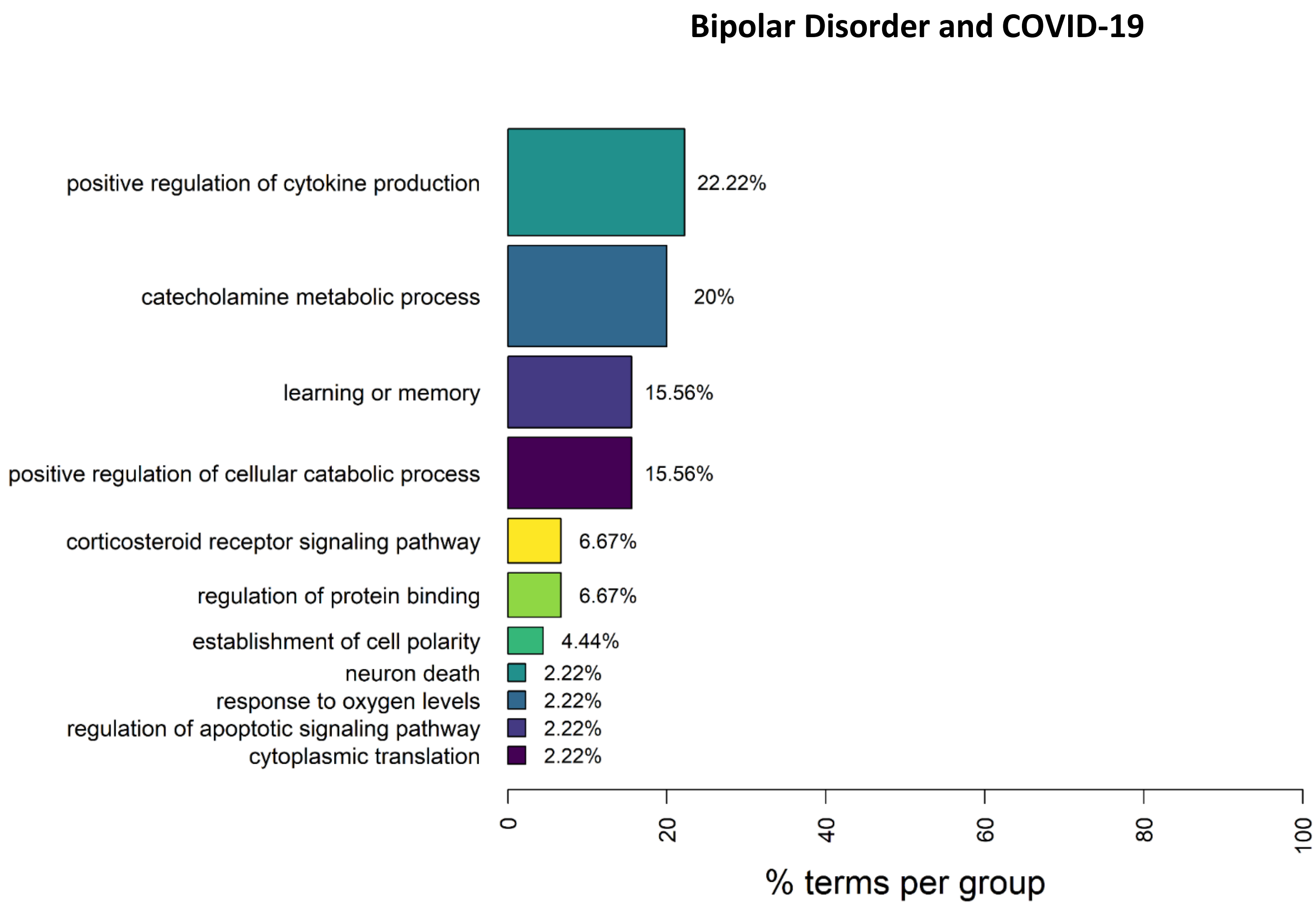

Supplementary Figure S2: Functional enrichement analysis results of the overalpping GO biological processes between Bipolar Disorder and COVID-19, indicating the functional groups and the percentage of terms that belong to each group.

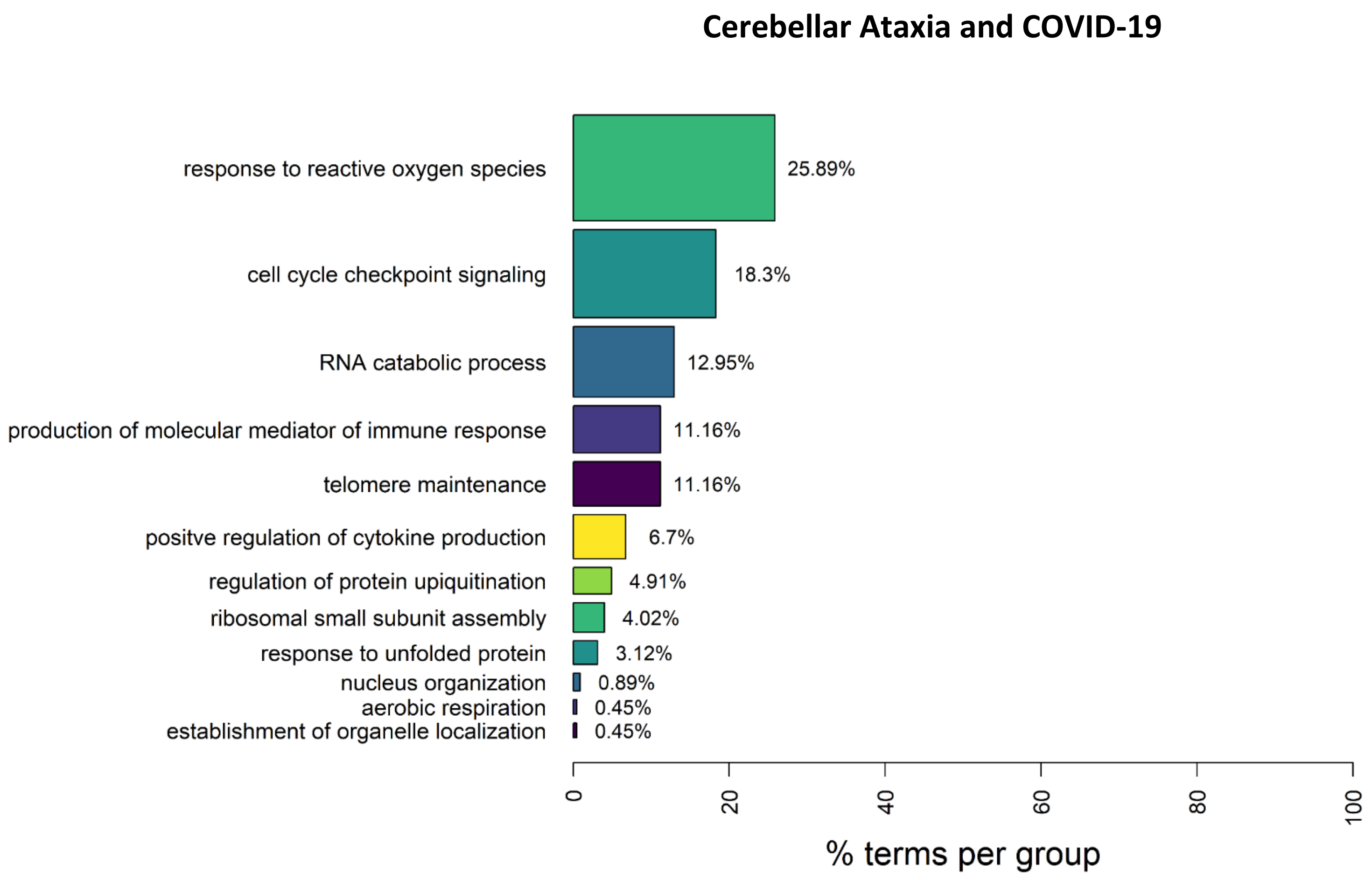

Supplementary Figure S3: Functional enrichement analysis results of the overlapping GO biological processes between Cerebellar Ataxia and COVID-19, indicating the functional groups and the percentage of terms that belong to each group.

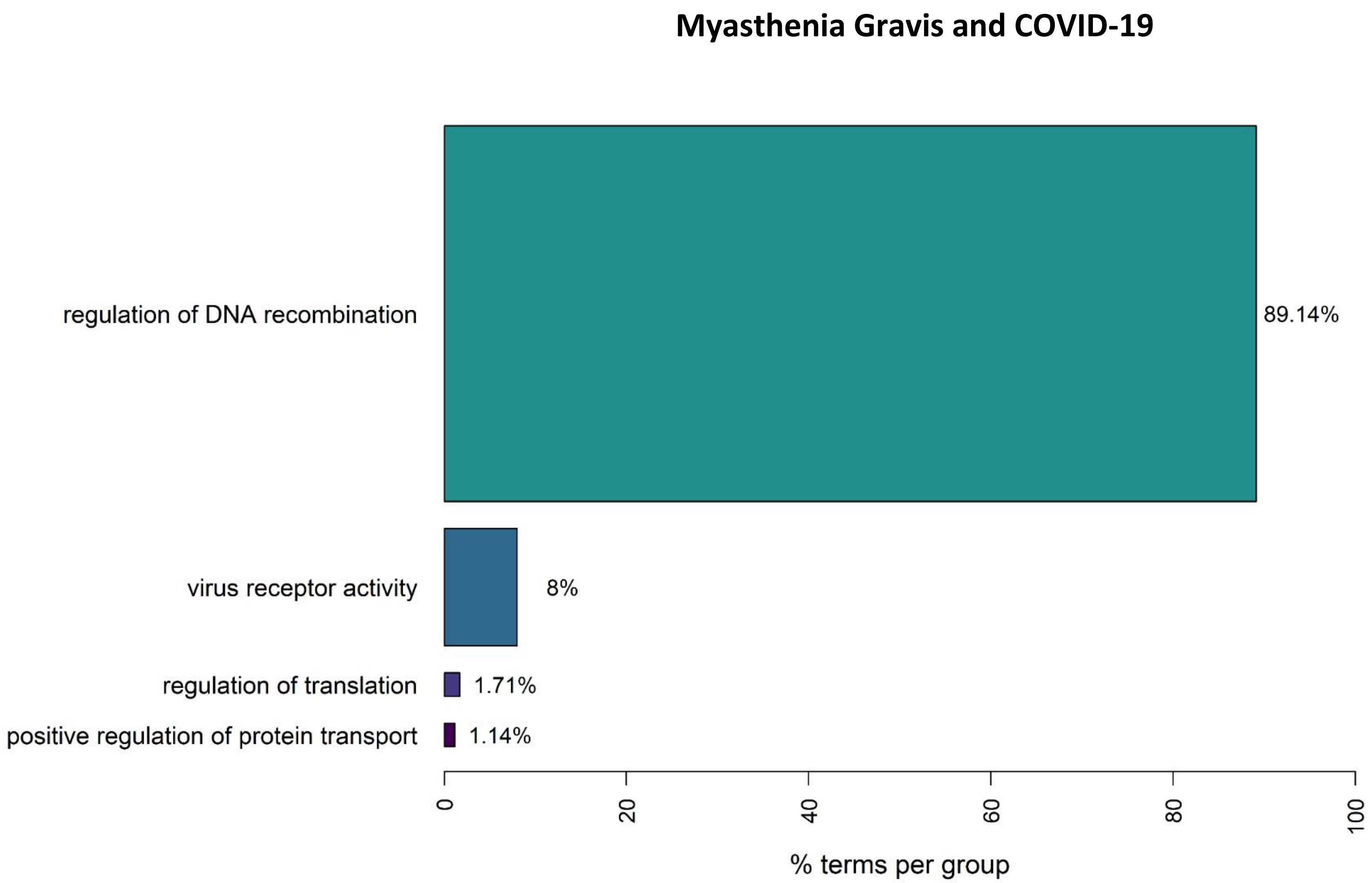

Supplementary Figure S4: Functional enrichement analysis results of the overlapping GO biological processes between Myasthenia Gravis and COVID-19, indicating the functional groups and the percentage of terms that belong to each group.

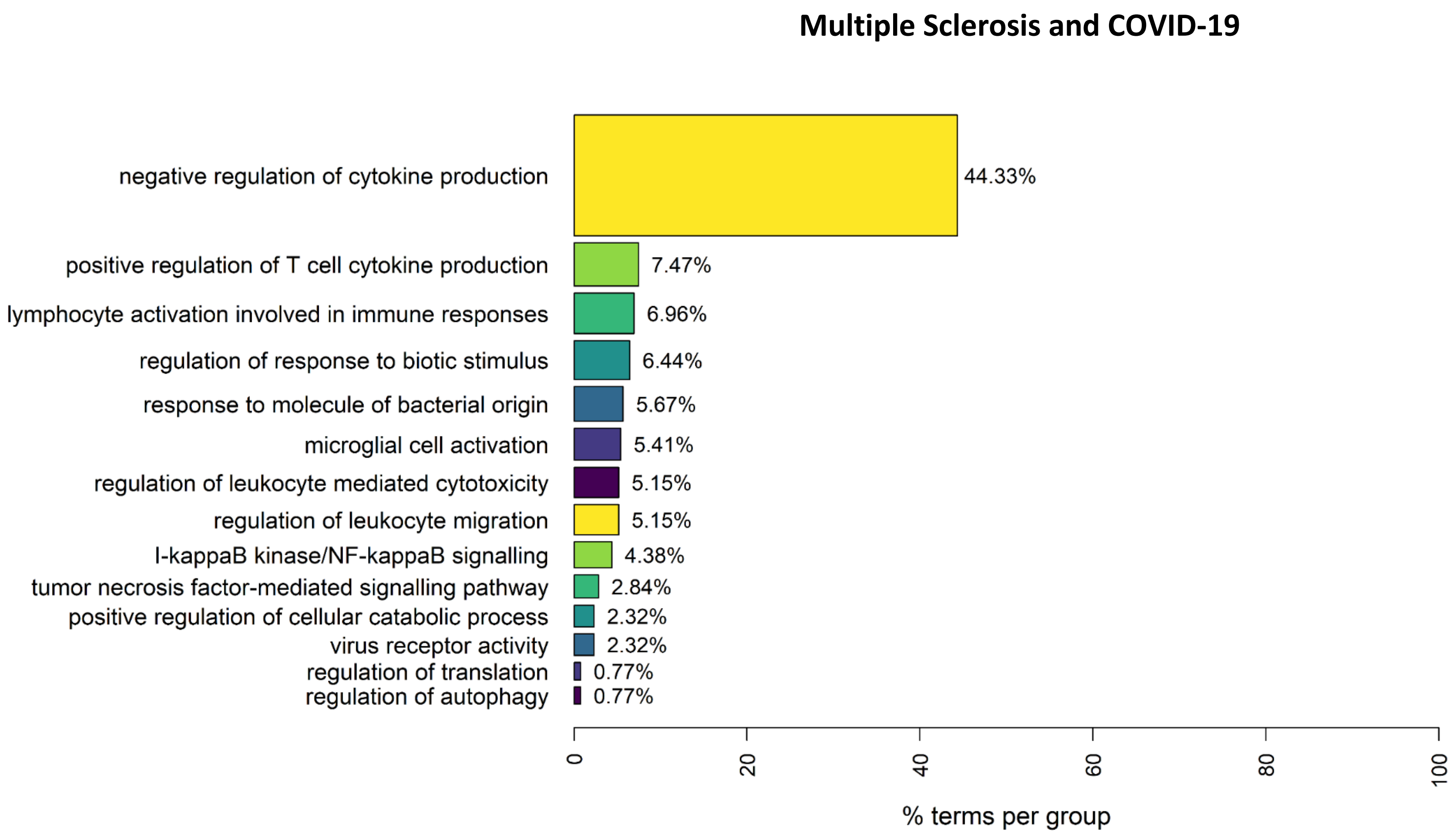

Supplementary Figure S5: Functional enrichement analysis results of the overlapping GO biological processes between Multiple Sclerosis and COVID-19, indicating the functional groups and the percentage of terms that belong to each group.

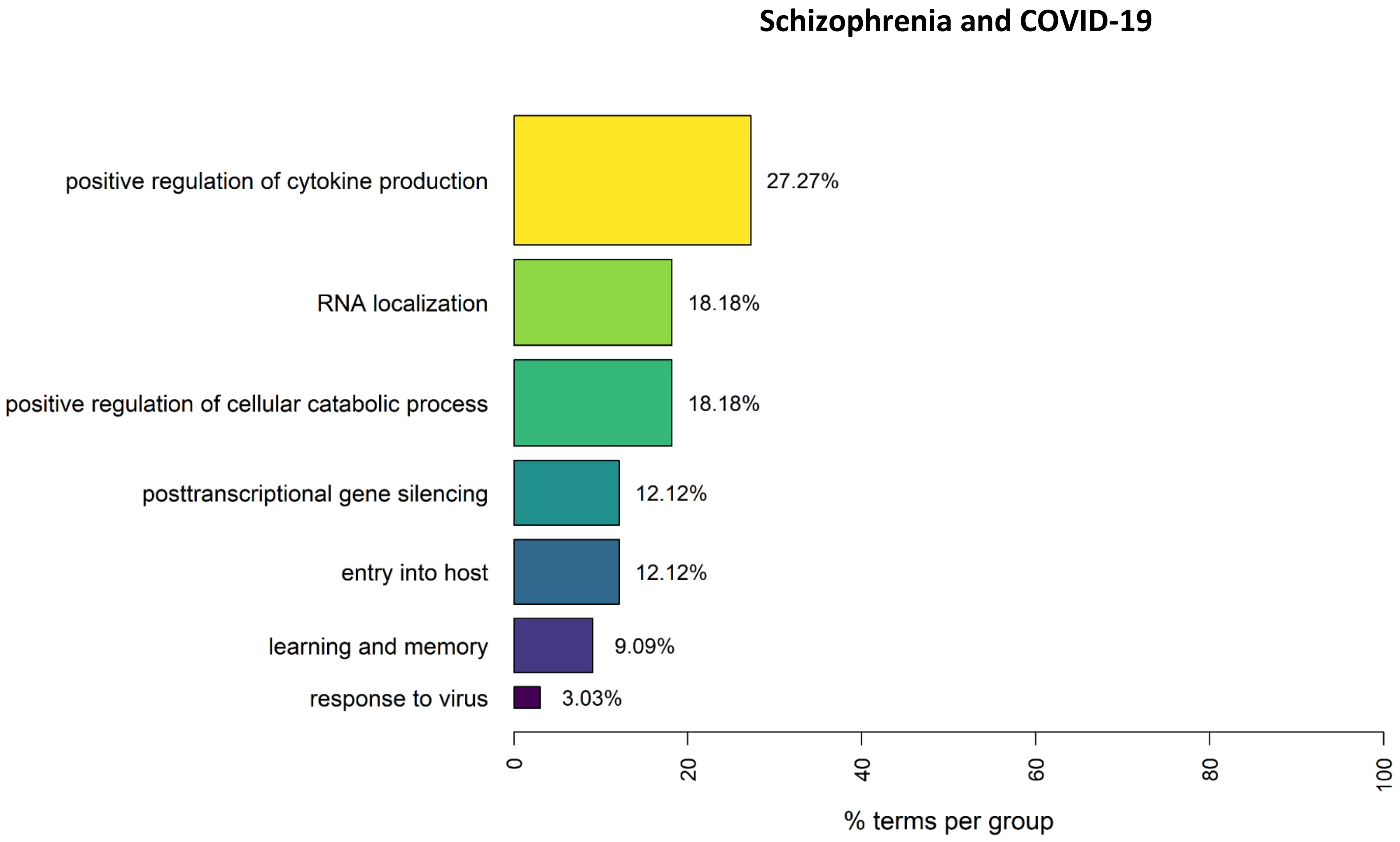

Supplementary Figure S6: Functional enrichement analysis results of the overlapping GO biological processes between Schizophrenia Disorder and COVID-19, indicating the functional groups and the percentage of terms that belong to each group.

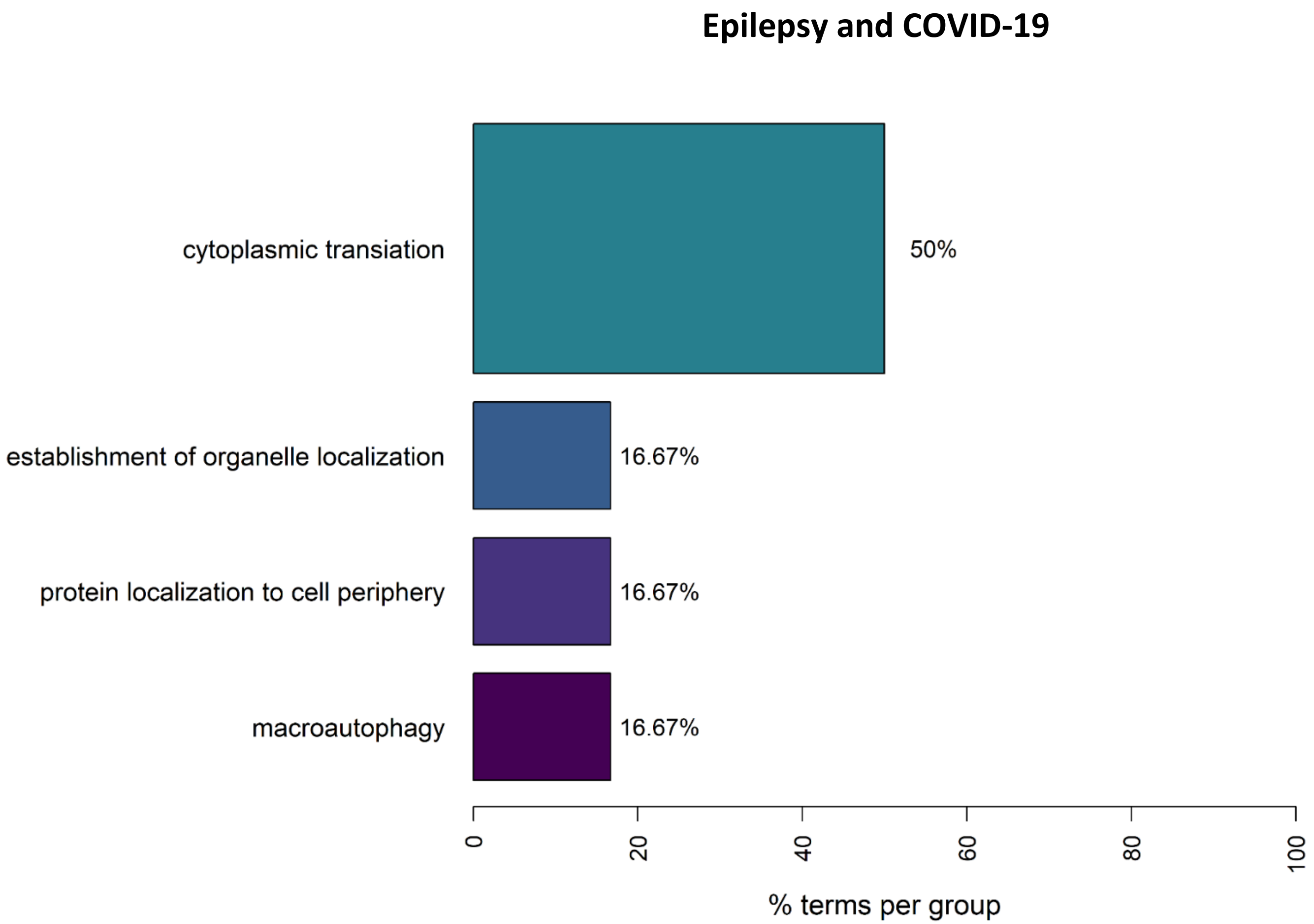

Supplementary Figure S7: Functional enrichement analysis results of the overlapping GO biological processes between Epilepsy and COVID-19, indicating the functional groups and the percentage of terms that belong to each group.

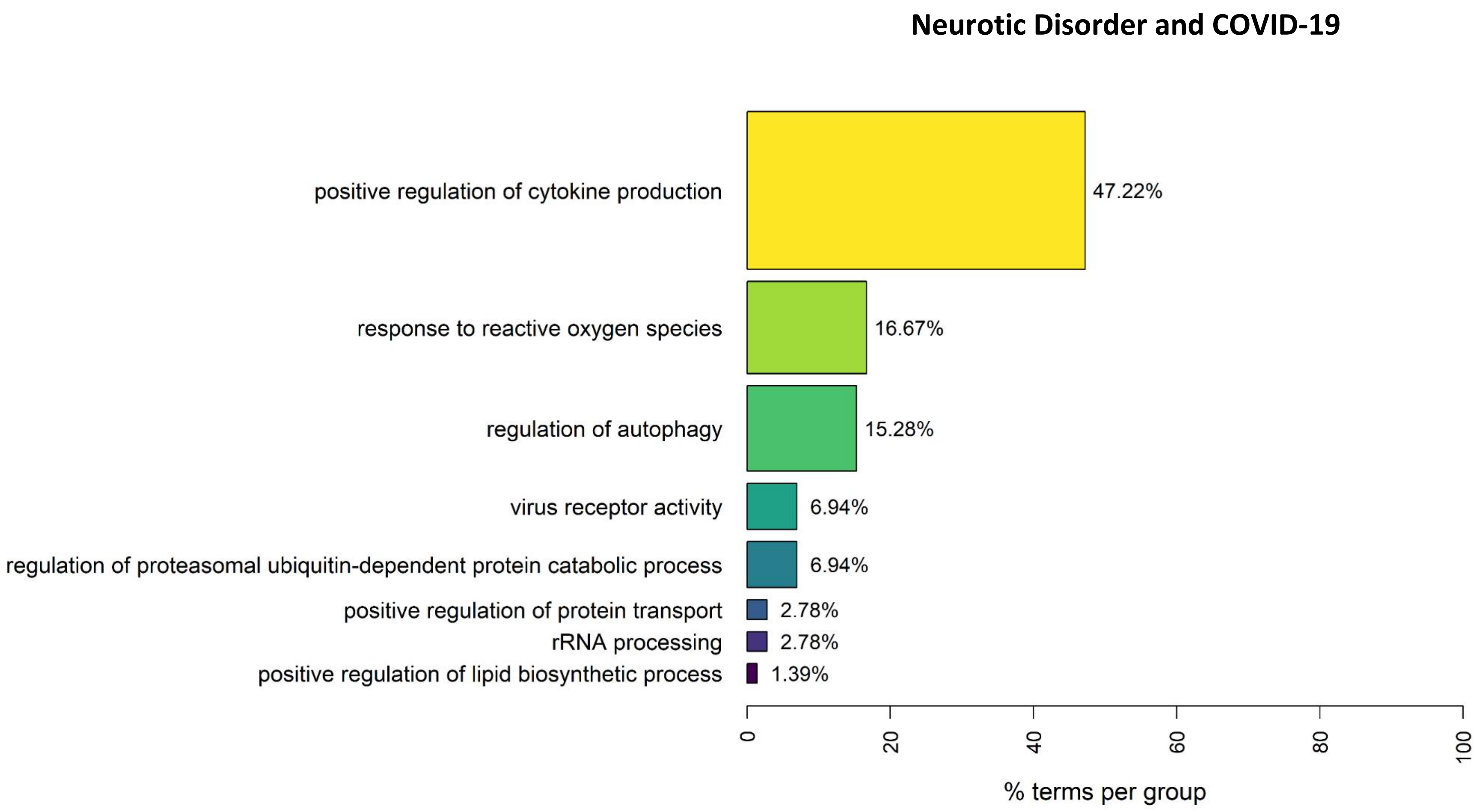

Supplementary Figure S8: Functional enrichement analysis results of the overlapping GO biological processes between Neurotic Disorder and COVID-19, indicating the functional groups and the percentage of terms that belong to each group.

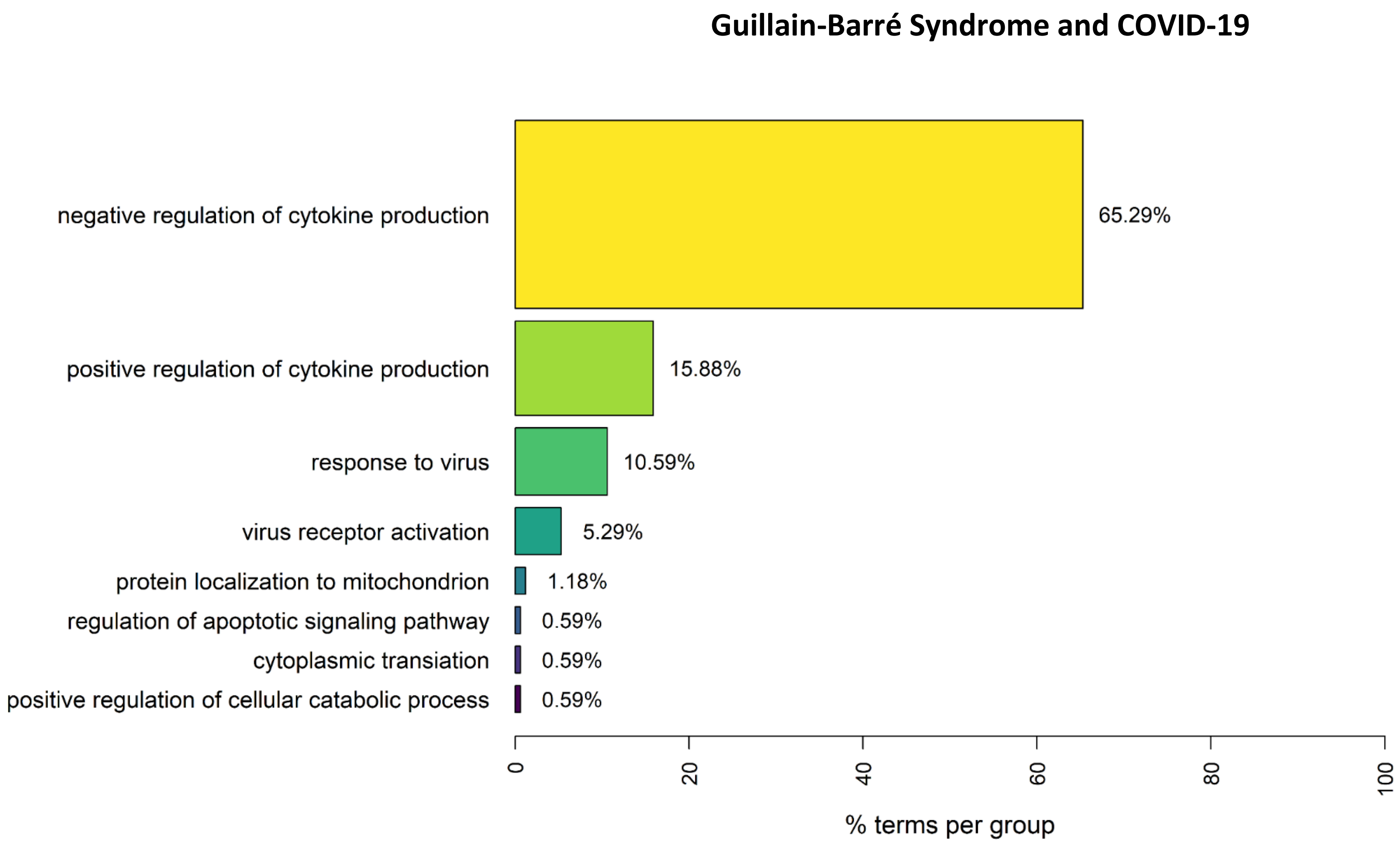

Supplementary Figure S9: Functional enrichement analysis results of the overlapping GO biological processes between Guillain-Barre Syndrome and COVID-19, indicating the functional groups and the percentage of terms that belong to each group.

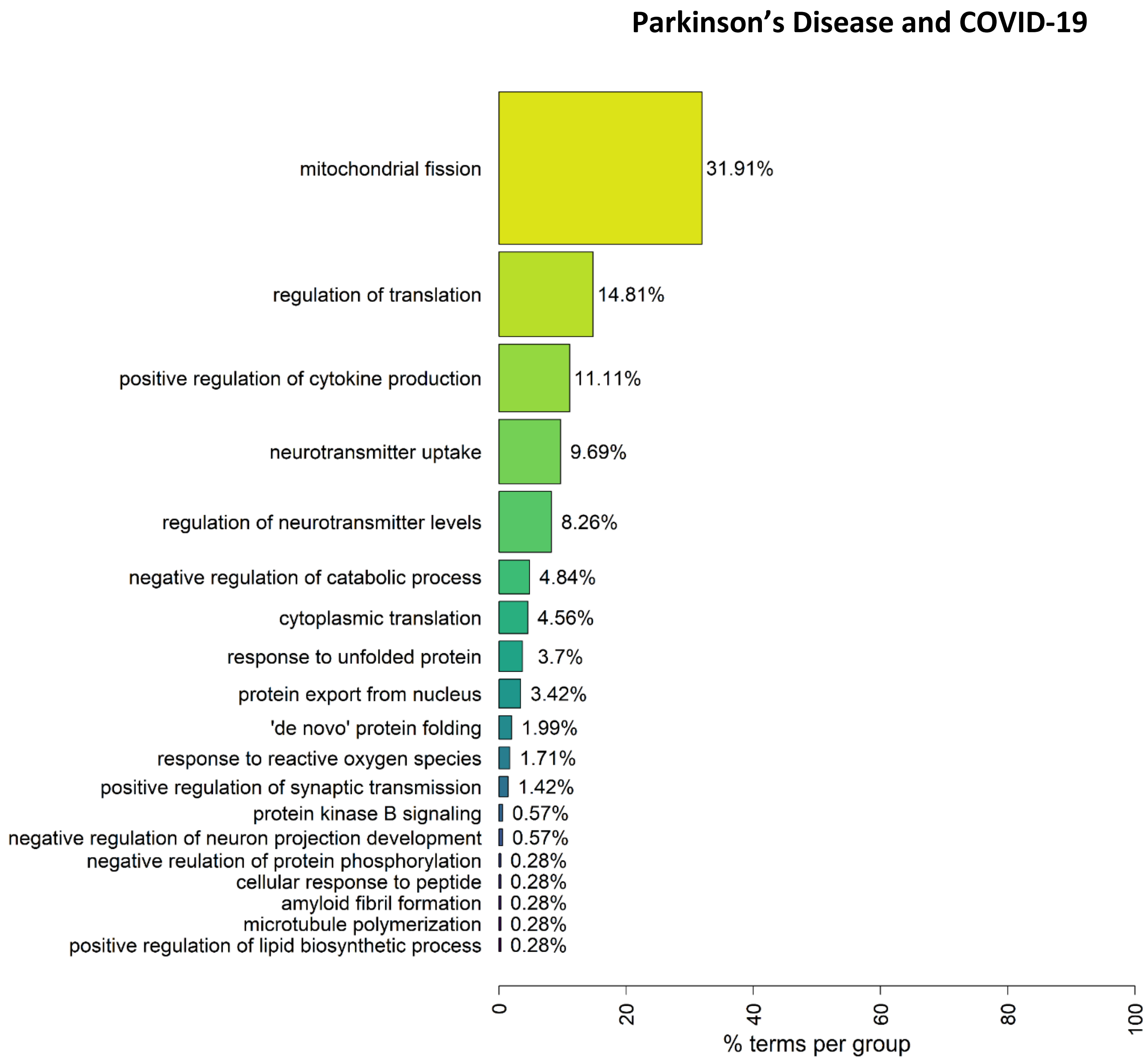

Supplementary Figure S10: Functional enrichement analysis results of the overlapping GO biological processes between Parkinson’s Disease and COVID-19, indicating the functional groups and the percentage of terms that belong to each group

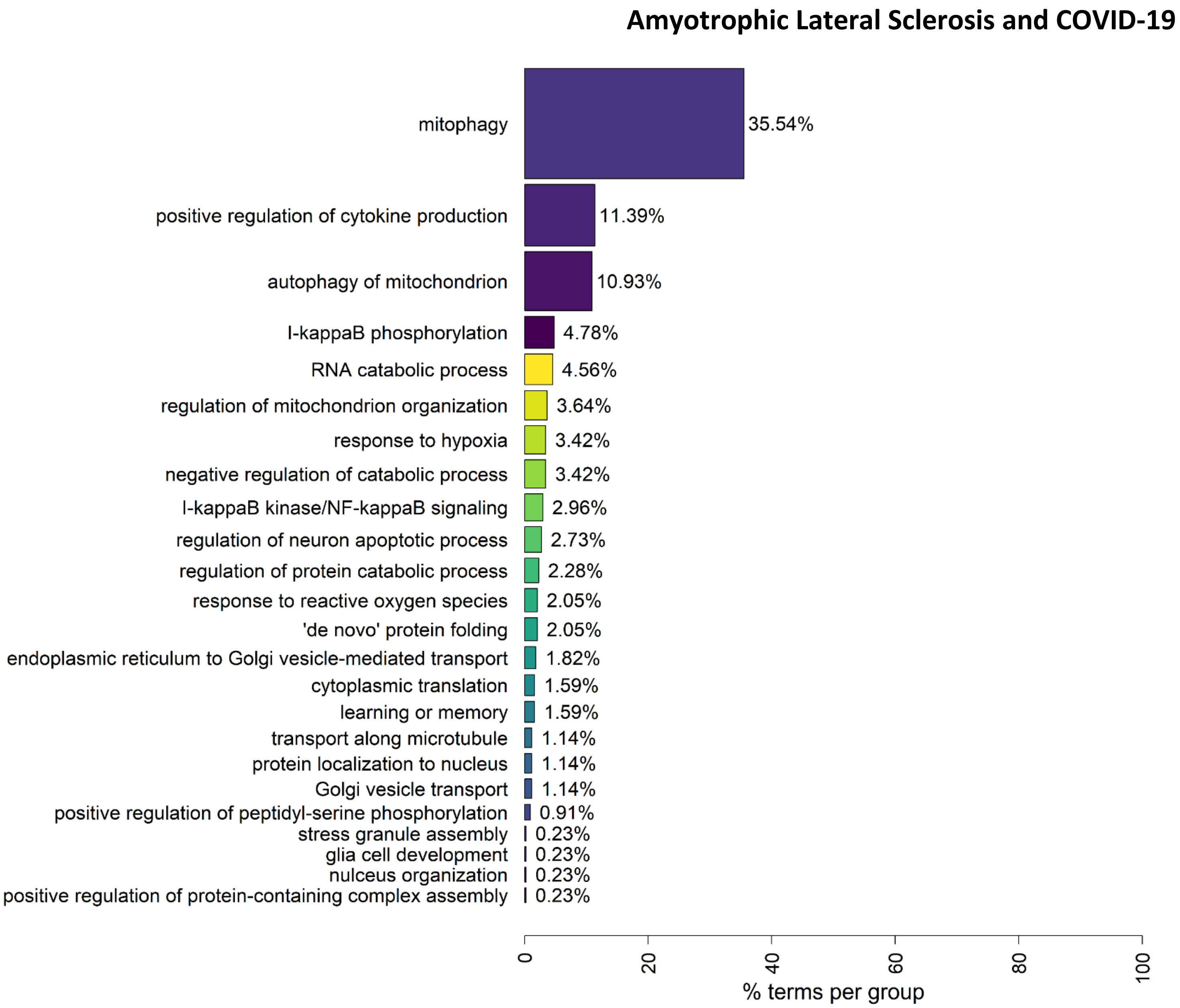

Supplementary Figure S11: Functional enrichement analysis results of the overlapping GO biological processes between Amyotrophic Lateral Sclerosis and COVID-19, indicating the functional groups and the percentage of terms that belong to each group

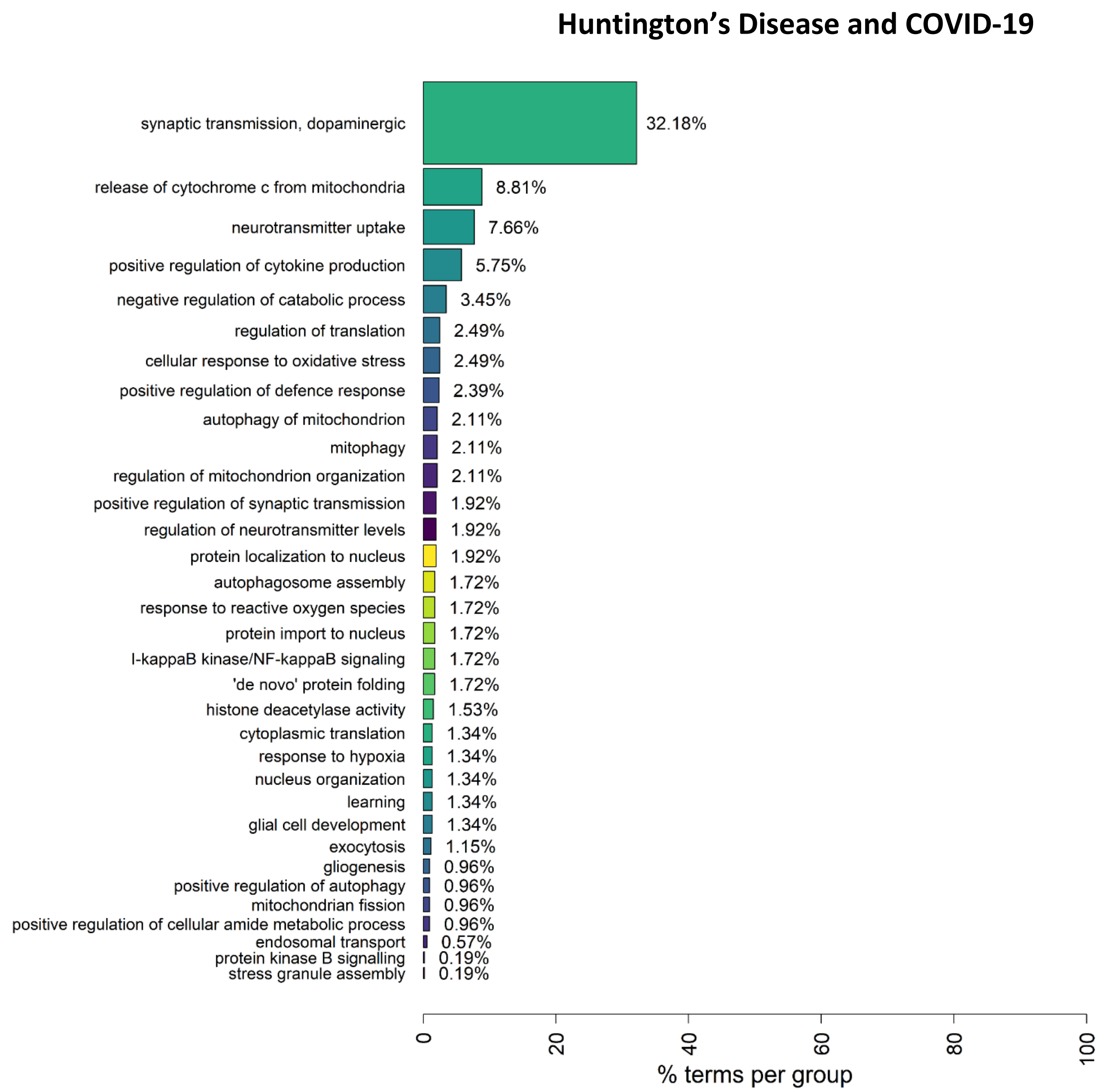

Supplementary Figure S12: Functional enrichement analysis results of the overlapping GO biological processes between Huntington’s Disease and COVID-19, indicating the functional groups and the percentage of terms that belong to each group

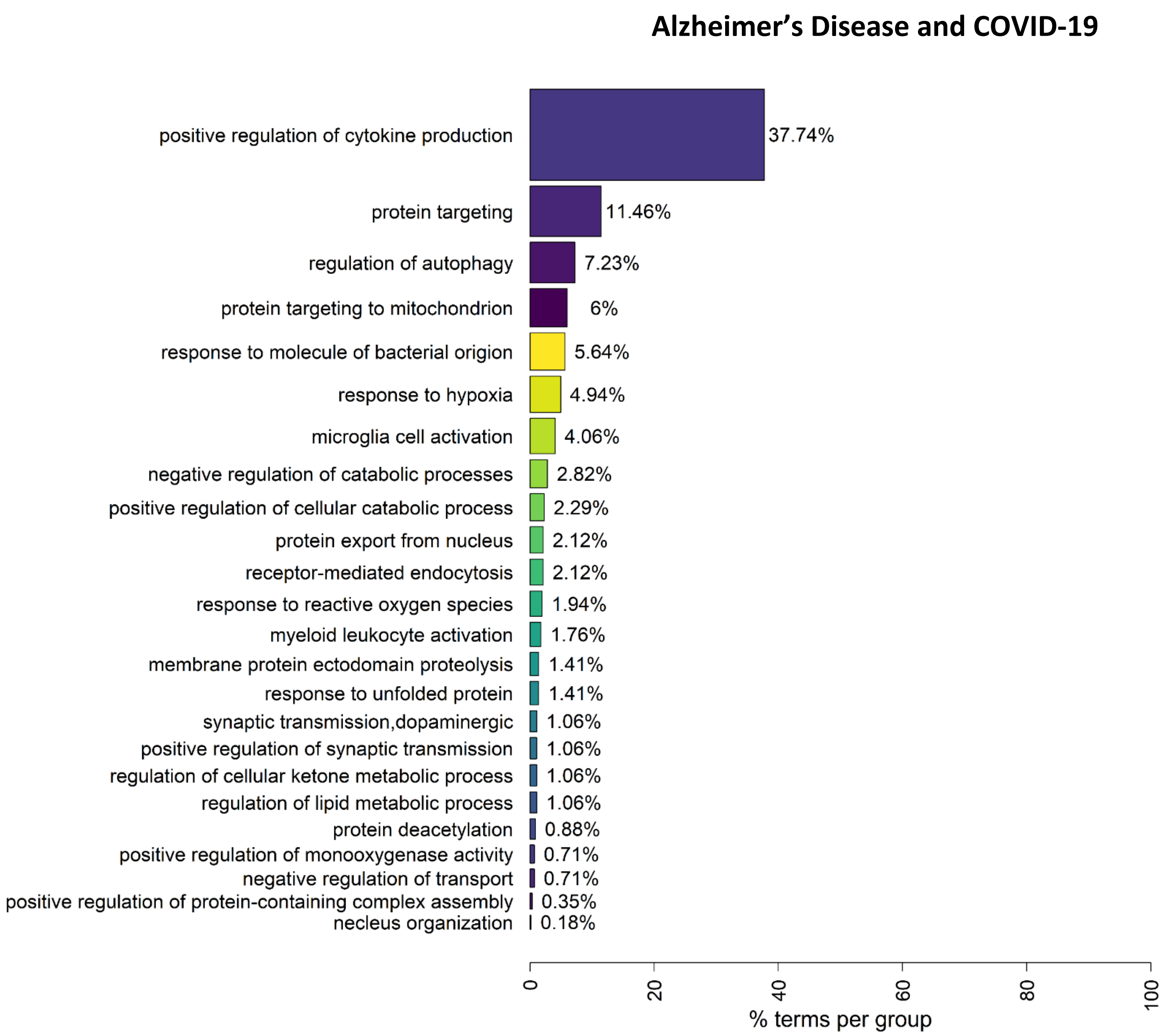

Supplementary Figure S13: Functional enrichement analysis results of the overlapping GO biological processes between Alzheimer's Disease and COVID-19, indicating the functional groups and the percentage of terms that belong to each group

Supplementary Table S1: Matching 5-mer linear motifs found in the consensus sequences between SARS-CoV-2 epitopes and autoreactive epitopes found in MS, with the 5-mer motif highlighted in their epitope sequences.

MS epitope ID	COVID-19 epitope ID	Consensus sequence	MS epitope sequence	COVID-19 epitope sequence
P10321	P0DTC9-16	ALLLL	APRALLLLL	LALLLLDRL
P10321	P0DTC9-42	A??ALLLL	APRALLLLL	GDAALALLLLDRLNQL
P10321	P0DTC9-93	A??ALLLL	APRALLLLL	DAALALLLLDRLNQL
P10321	P0DTC9-118	A??ALLLL	APRALLLLL	PARMAGNGGDAALALLLLDRLNQLESKMSGKGQQQ QGQ
P10321	P0DTC9-120	A??ALLLL	APRALLLLL	AALALLLLDRLNQLE
P10321	P0DTC9-157	A??ALLLL	APRALLLLL	AGNGGDAALALLLLD
P10321	P0DTC9-216	A??ALLLL	APRALLLLL	AALALLLLDRLNQLES
P10321	P0DTC9-221	A??ALLLL	APRALLLLL	AGNGGDAALALLLLDR
P10321	P0DTC9-224	ALLLL	APRALLLLL	ALALLLLDRLNQLESK
P10321	P0DTC9-225	ALLLL	APRALLLLL	ALLLLDRLNQLESKMS
P10321	P0DTC9-229	A??ALLLL	APRALLLLL	ARMAGNGGDAALALLLLDRLNQLESKMSGKG
P10321	P0DTC9-231	A??ALLLL	APRALLLLL	DAALALLLLDRLNQLE
P10321	P0DTC9-245	A??ALLLL	APRALLLLL	GGDAALALLLLDRLNQ
P10321	P0DTC9-249	A??ALLLL	APRALLLLL	GNGGDAALALLLLDRL
P10321	P0DTC9-262	ALLLL	APRALLLLL	LALLLLDRLNQLESKM
P10321	P0DTC9-277	A??ALLLL	APRALLLLL	MAGNGGDAALALLLLD
P10321	P0DTC9-282	A??ALLLL	APRALLLLL	NGGDAALALLLLDRLN
P10321	P0DTC9-313	A??ALLLL	APRALLLLL	RMAGNGGDAALALLLL
P10321	P0DTC9-339	A??ALLLL	APRALLLLL	SSRGTSPARMAGNGGDAALALLLLDRLNQLESKMSGK GQ
P10321	P0DTC9-359	A??ALLLL	APRALLLLL	AALALLLLDRLNQLESKMS
P10321	P0DTC9-363	ALLLL	APRALLLLL	ALLLLDRLNQLESKMSG
P10321	P0DTC9-394	A??ALLLL	APRALLLLL	MAGNGGDAALALLLLDR
P10321	P0DTC9-417	A??ALLLL	APRALLLLL	RMAGNGGDAALALLLLDRL
P10321	P0DTC9-438	ALLLL	APRALLLLL	ALALLLLDRLNQLES
P10321	P0DTC9-439	ALLLL	APRALLLLL	ALLLLDRLNQLESKM
P10321	P0DTC9-453	A??ALLLL	APRALLLLL	GGDAALALLLLDRLN
P10321	P0DTC9-456	A??ALLLL	APRALLLLL	GNGGDAALALLLLDR

P10321	P0DTC9-480	A??ALLLL	APRALLLLL	MAGNGGDAALALLLL
J3QL64-2	P0DTD1-241	YLATA	ASQKRPSQRHGSKYLATAST	YLATALLTL
J3QL64-2	P0DTC1-161	YLATA	ASQKRPSQRHGSKYLATAST	ADNNCYLATALLTLQ
J3QL64-2	P0DTC1-253	YLATA	ASQKRPSQRHGSKYLATAST	CYLATALLTLQQIEL
Q53Z42-3	P0DTD1-602	APRTL?	VMAPRTLVL	DPAQLPAPRTLLTK
Q53Z42-3	P0DTD1-1085	APRTL?	VMAPRTLVL	PAPRTLLTKGTLEPE
Q53Z42-3	P0DTD1-1117	APRTL?	VMAPRTLVL	QLPAPRTLLTKGTLE
Q53Z42-3	P0DTD1-1302	APRTL?	VMAPRTLVL	YIGDPAQLPAPRTLL
P09543-2	P0DTD1-274	??GKPVP	GNGRWMLTLAKNMEVRAIFT GYYGKGKPVPTQG	DASGKPVPY
P09543-2	P0DTC1-256	??GKPVP	GNGRWMLTLAKNMEVRAIFT GYYGKGKPVPTQG	DASGKPVPYCYDTNV
P09543-2	P0DTC1-314	??GKPVP	GNGRWMLTLAKNMEVRAIFT GYYGKGKPVPTQG	ECTIFKDASGKPVPY
P09543-2	P0DTC1-1010	?GKPVP	GNGRWMLTLAKNMEVRAIFT GYYGKGKPVPTQG	SGKPVPYCYDTNVLE
P02686-14	P0DTD1-241	YLATA	KYLATASTMDHARHGFLPRH	YLATALLTL
P02686-14	P0DTC1-161	YLATA	KYLATASTMDHARHGFLPRH	ADNNCYLATALLTLQ
P02686-14	P0DTC1-253	YLATA	KYLATASTMDHARHGFLPRH	CYLATALLTLQQIEL
J3QL64-16	P0DTD1-241	YLATA	ASQKRPSQRHGSKYLATASTM	YLATALLTL
J3QL64-16	P0DTC1-161	YLATA	ASQKRPSQRHGSKYLATASTM	ADNNCYLATALLTLQ
J3QL64-16	P0DTC1-253	YLATA	ASQKRPSQRHGSKYLATASTM	CYLATALLTLQQIEL
J3QL64-17	P0DTD1-241	YLATA	ASQKRPSQRHGSKYLATASTM DHARHGFLPRHRDTGILDSIGR F	YLATALLTL
J3QL64-17	P0DTC1-161	YLATA	ASQKRPSQRHGSKYLATASTM DHARHGFLPRHRDTGILDSIGR F	ADNNCYLATALLTLQ
J3QL64-17	P0DTC1-253	YLATA	ASQKRPSQRHGSKYLATASTM DHARHGFLPRHRDTGILDSIGR F	CYLATALLTLQQIEL
P02686-19	P0DTD1-241	YLATA	RHGSKYLATASTMDHARHGFL	YLATALLTL
P02686-19	P0DTC1-161	YLATA	RHGSKYLATASTMDHARHGFL	ADNNCYLATALLTLQ

P02686-19	P0DTC1-253	YLATA	RHGSKYLATASTMDHARHGFL	CYLATALLTLQQIEL
P10809-2	P0DTC1-566	IPKEE??P	VVTEIPKEEKDPGM	IPKEEVKPFITESKP
Q13875-8	P0DTC9-1	?SRGGS	QPRSSPLRGPGASRGGSPVKAS RF	AEGSRGGSQA
Q13875-8	P0DTC9-332	?SRGGS	QPRSSPLRGPGASRGGSPVKAS RF	LPQGTTLPKGFYAEGSRGGSQASSRSSSRSRNSSRNSTP
Q13875-8	P0DTC9-335	?SRGGS	QPRSSPLRGPGASRGGSPVKAS RF	QGTTLPKGFYAEGSRGGS
Q13875-8	P0DTC9-448	?SRGGS	QPRSSPLRGPGASRGGSPVKAS RF	EGSRGGSQASSRSSS
Q13875-8	P0DTC9-501	SRGGS	QPRSSPLRGPGASRGGSPVKAS RF	SRGGSQASSRSSSRS
A0A075B6H9-1	P0DTC9-3	?PSASA	QSVLTQSPSASAS	APSASAFFGM
A0A075B6H9-1	P0DTC9-4	?PSASA	QSVLTQSPSASAS	AQFAPSASA
A0A075B6H9-1	P0DTC9-5	?PSASA	QSVLTQSPSASAS	AQFAPSASAFFGMSR
A0A075B6H9-1	P0DTC9-38	?PSASA	QSVLTQSPSASAS	FAPSASAFF
A0A075B6H9-1	P0DTC9-73	?PSASA	QSVLTQSPSASAS	WPQIAQFAPSASAFF
A0A075B6H9-1	P0DTC9-103	?PSASA	QSVLTQSPSASAS	QFAPSASAFFGMSRI
A0A075B6H9-1	P0DTC9-107	?PSASA	QSVLTQSPSASAS	AQFAPSASAF
A0A075B6H9-1	P0DTC9-178	?PSASA	QSVLTQSPSASAS	QFAPSASAFF
A0A075B6H9-1	P0DTC9-405	PSASA	QSVLTQSPSASAS	PSASAFFGMSRIGMEVTPS
A0A075B6H9-1	P0DTC9-440	?PSASA	QSVLTQSPSASAS	APSASAFFGMSRIGM
A0A075B6H9-1	P0DTC9-461	?PSASA	QSVLTQSPSASAS	IAQFAPSASAFFGMS
P02686-35	P0DTD1-241	YLATA	SKYLATASTMDHARHGFLPR	YLATALLTL
P02686-35	P0DTC1-161	YLATA	SKYLATASTMDHARHGFLPR	ADNNCYLATALLTLQ
P02686-35	P0DTC1-253	YLATA	SKYLATASTMDHARHGFLPR	CYLATALLTLQQIEL
P02686-36	P0DTD1-241	YLATA	SQRHGSKYLATASTMDHARH	YLATALLTL
P02686-36	P0DTC1-161	YLATA	SQRHGSKYLATASTMDHARH	ADNNCYLATALLTLQ
P02686-36	P0DTC1-253	YLATA	SQRHGSKYLATASTMDHARH	CYLATALLTLQQIEL
P37837-24	P0DTD1-920	?NYYKK	YNYYKKFSYKTIVMG	IGEAVKTQFNYYKKV
P37837-24	P0DTC1-94	NYYKK	YNYYKKFSYKTIVMG	LDNYYKKDNSYFTE
P37837-24	P0DTC1-536	NYYKK	YNYYKKFSYKTIVMG	IDPKLDNYYKKDNSY
P37837-24	P0DTC1-683	NYYKK	YNYYKKFSYKTIVMG	LDNYYKKDNSYFTEQ

P37837-24	P0DTC1-886	NYYKK	YNYYKKFSYKTIVMG	NYYKKDNSYFTEQPI
P37837-24	P0DTC1-1070	NYYKK	YNYYKKFSYKTIVMG	TEIDPKLDNYYKKDN
J3QL64-25	P0DTD1-241	YLATA	ASQKRPSQRHGSKYLATASTM DHARHGFLPRHRDTGIL	YLATALLTL
J3QL64-25	P0DTC1-161	YLATA	ASQKRPSQRHGSKYLATASTM DHARHGFLPRHRDTGIL	ADNNCYLATALLTLQ
J3QL64-25	P0DTC1-253	YLATA	ASQKRPSQRHGSKYLATASTM DHARHGFLPRHRDTGIL	CYLATALLTLQQIEL
P02686-38	P0DTD1-241	YLATA	GSKYLATASTMDHARHGFL	YLATALLTL
P02686-38	P0DTC1-161	YLATA	GSKYLATASTMDHARHGFL	ADNNCYLATALLTLQ
P02686-38	P0DTC1-253	YLATA	GSKYLATASTMDHARHGFL	CYLATALLTLQQIEL
J3QL64-27	P0DTD1-241	YLATA	GSKYLATASTMDHARHGFLP	YLATALLTL
J3QL64-27	P0DTC1-161	YLATA	GSKYLATASTMDHARHGFLP	ADNNCYLATALLTLQ
J3QL64-27	P0DTC1-253	YLATA	GSKYLATASTMDHARHGFLP	CYLATALLTLQQIEL
P02686-44	P0DTD1-241	YLATA	KYLATASTM	YLATALLTL
P02686-44	P0DTC1-161	YLATA	KYLATASTM	ADNNCYLATALLTLQ
P02686-44	P0DTC1-253	YLATA	KYLATASTM	CYLATALLTLQQIEL
P02686-56	P0DTD1-241	YLATA	ASQKRPSQRHGKYLATAST	YLATALLTL
P02686-56	P0DTC1-161	YLATA	ASQKRPSQRHGKYLATAST	ADNNCYLATALLTLQ
P02686-56	P0DTC1-253	YLATA	ASQKRPSQRHGKYLATAST	CYLATALLTLQQIEL
P60201-47	P0DTC1-214	ATLVS	AAFVGAAATLVSLLTFMIAATY NFAVLKLMGR	ATLVSDIDITFLKKD
P60201-47	P0DTC1-294	?ATLVS	AAFVGAAATLVSLLTFMIAATY NFAVLKLMGR	DSATLVSDIDITFLK
P60201-47	P0DTC1-508	?ATLVS	AAFVGAAATLVSLLTFMIAATY NFAVLKLMGR	HPDSATLVSDIDITF
P60201-47	P0DTC1-838	?ATLVS	AAFVGAAATLVSLLTFMIAATY NFAVLKLMGR	NGNLHPDSATLVSDI
P60201-47	P0DTC1-857	?ATLVS	AAFVGAAATLVSLLTFMIAATY NFAVLKLMGR	NLHPDSATLVSDIDI
J3QL64-91	P0DTD1-241	YLATA	GSKYLATASTMDHARHGFLPR HRDTGILDSIGRFFGGDRG	YLATALLTL

J3QL64-91	P0DTC1-161	YLATA	GSKYLATASTMDHARHGFLPR HRDTGILDSIGRFFGGDRG	ADNNCYLATALLTLQ
J3QL64-91	P0DTC1-253	YLATA	GSKYLATASTMDHARHGFLPR HRDTGILDSIGRFFGGDRG	CYLATALLTLQQIEL
P60201-50	P0DTC1-790	FFFLY	GTASFFFLYGALLLAYGFYTTGA VRQIFGDYK	LVQSTQWSLFFFLYE
P60201-50	P0DTC1-1247	FFFLY????L??	GTASFFFLYGALLLAYGFYTTGA VRQIFGDYK	WSLFFFLYENAFLPF
P0DTU3-14	P0DTC2-95	FLLKY	YPNRGLQFLLKYITGDNLVKGS	QPRTFLLKYNENGTI
P0DTU3-14	P0DTC2-215	FLLKY	YPNRGLQFLLKYITGDNLVKGS	AYYVGYLQPRTFLLKYNENG
P0DTU3-14	P0DTC2-282	FLLKY	YPNRGLQFLLKYITGDNLVKGS	TFLLKYNENGTITDAVDCAL
P0DTU3-14	P0DTC2-539	FLLKY	YPNRGLQFLLKYITGDNLVKGS	RTFLLKYNENGTITD
P0DTU3-14	P0DTC2-577	FLLKY	YPNRGLQFLLKYITGDNLVKGS	YYVGYLQPRTFLLKY
P0DTU3-14	P0DTC2-931	FLLKY	YPNRGLQFLLKYITGDNLVKGS	GAAAYYVGYLQPRTFLLKYNENGTITDAVDCALDPLSET
P0DTU3-14	P0DTC2-956	FLLKY	YPNRGLQFLLKYITGDNLVKGS	PRTFLLKYNENGTITDA
P0DTU3-14	P0DTC2-1020	FLLKY	YPNRGLQFLLKYITGDNLVKGS	FLLKYNENGTITDAV
P0DTU3-14	P0DTC2-1267	FLLKY	YPNRGLQFLLKYITGDNLVKGS	VGYLQPRTFLLKYNE
A0A0A6YYK6	P0DTD1-946	SRQRL	YSRQRLQLLLRHISRESI	ISRQRLTKYTMADLV
Q8IUQ4	P0DTD1-1148	LPTGT	ATALPTGTSK	RQWLPTGTLLVDSDL
Q8IUQ4	P0DTD1-1286	LPTGT	ATALPTGTSK	WLPTGTLLVDSDLND
Q6UWS5	P0DTD1-749	TVAGV??	ATVAGVHVK	ARTVAGVSICSTMTN
Q6UWS5	P0DTD1-941	TVAGV??	ATVAGVHVK	ISAKNRARTVAGVSI
P04271-1	P0DTC1-1	?L?NNELS	ELINNELSHF	AVKLQNNELSPVALR
P04271-1	P0DTC1-100	?L?NNELS	ELINNELSHF	LRANSAVKLQNNELS
P04271-1	P0DTC1-939	NNELS	ELINNELSHF	QNNELSPVALRQMSC
P04271-1	P0DTC1-960	?L?NNELS	ELINNELSHF	RANSAVKLQNNELSP
Q9H209	P0DTC1-2	??VATVQ?	FSVATVQTTW	CIKVATVQSKMSDVK
Q9H209	P0DTC1-494	??VATVQ?	FSVATVQTTW	GVGGKPCIKVATVQS
P02686-84	P0DTD1-241	YLATA	HGSKYLATASTMDHA	YLATALLTL
P02686-84	P0DTC1-161	YLATA	HGSKYLATASTMDHA	ADNNCYLATALLTLQ
P02686-84	P0DTC1-253	YLATA	HGSKYLATASTMDHA	CYLATALLTLQQIEL
Q01082-1	P0DTD1-627	???SSVEL	KPRDVSSVEL	GFFKEGSSVELKHF
Q01082-1	P0DTD1-859	???SSVEL	KPRDVSSVEL	FFKEGSSVELKHFFF

O95674	P0DTC2-188	LNRAL???	PEVLNRALSNL	TQLNRALTGI
O95674	P0DTC2-298	LNRAL???	PEVLNRALSNL	YGSFCTQLNRALTGIAVEQD
O95674	P0DTC2-406	LNRAL???	PEVLNRALSNL	TQLNRALTGIAVEQD
O95674	P0DTC2-434	LNRAL???	PEVLNRALSNL	CTQLNRALTGIAVEQDKNTQEVFAQVKQI
O95674	P0DTC2-506	LNRAL	PEVLNRALSNL	LLQYGSFCTQLNRAL
O95674	P0DTC2-903	LNRAL???	PEVLNRALSNL	TQLNRALTGIAVEQDK
O95674	P0DTC2-941	LNRAL???	PEVLNRALSNL	TQLNRALTGIAVEQDKNTQEVFAQVKQIYKTPPIKDFG G
O95674	P0DTC2-1141	LNRAL???	PEVLNRALSNL	TQLNRALTGIAVEQDKNTQ
O95674	P0DTC2-1142	LNRAL???	PEVLNRALSNL	TQLNRALTGIAVEQDKNTQEVFA
O95674	P0DTC2-1190	LNRAL???	PEVLNRALSNL	FCTQLNRALTGIAVE
O95674	P0DTC2-1221	LNRAL???	PEVLNRALSNL	LNRALTGIAVEQDKN
P02686-87	P0DTD1-241	YLATA	RHGSKYLATA	YLATALLTL
P02686-87	P0DTC1-161	YLATA	RHGSKYLATA	ADNNCYLATALLTLQ
P02686-87	P0DTC1-253	YLATA	RHGSKYLATA	CYLATALLTLQQIEL
P02686-88	P0DTD1-241	YLATA	RHGSKYLATASTMDHARH	YLATALLTL
P02686-88	P0DTC1-161	YLATA	RHGSKYLATASTMDHARH	ADNNCYLATALLTLQ
P02686-88	P0DTC1-253	YLATA	RHGSKYLATASTMDHARH	CYLATALLTLQQIEL
Q16663	P0DTD1-795	SVAAL?	SVAALSCLMLVA	DKRTTCFSVAALTNN
Q16663	P0DTD1-1012	SVAAL?	SVAALSCLMLVA	LLDKRTTCFSVAALT
Q16663	P0DTD1-1150	SVAAL?	SVAALSCLMLVA	RTTCFSVAALTNNVA
Q16663	P0DTD1-1189	SVAAL?	SVAALSCLMLVA	TCFSVAALTNNVAFQ
P14174-1	P0DTD1-633	VPRAS	TNVPRASVPDGFLSEL	HNKCAYWVPRAS
P42701	P0DTC1-176	STVLS	WSTVLSTYHF	AGNATEVPANSTVLS
P01042-1	P0DTC1-102	AVDAA	KDLFKAVDAALKK	LSFCAFAVDAAKAY
Q13571-1	P0DTC1-497	L???SKTPE	LPSYEEALSLPSKTPE	GYLTSSSKTPEEHFI
Q13571-1	P0DTC1-784	L???SKTPE	LPSYEEALSLPSKTPE	LTSSSKTPEEHFIET
Q13571-1	P0DTC1-1020	SKTPE	LPSYEEALSLPSKTPE	SKTPEEHFIETISLA
Q13571-1	P0DTC1-1033	SKTPE	LPSYEEALSLPSKTPE	SSSKTPEEHFIETIS
Q13571-1	P0DTC1-1057	?Y???L???SKTPE	LPSYEEALSLPSKTPE	TAYNGYLTSSSKTPE
Q13571-1	P0DTC1-1282	Y???L???SKTPE	LPSYEEALSLPSKTPE	YNGYLTSSSKTPEEH
Q13571-2	P0DTC1-497	L???SKTPE	VVLPSYEEALSLPSKTPE	GYLTSSSKTPEEHFI
Q13571-2	P0DTC1-784	L???SKTPE	VVLPSYEEALSLPSKTPE	LTSSSKTPEEHFIET

Q13571-2	P0DTC1-1020	SKTPE	VVLPSYEEALSLPSKTPE	SKTPEEHFIETISLA
Q13571-2	P0DTC1-1033	SKTPE	VVLPSYEEALSLPSKTPE	SSSKTPEEHFIETIS
Q13571-2	P0DTC1-1057	?Y???L???SKTPE	VVLPSYEEALSLPSKTPE	TAYNGYLTSSSKTPE
Q13571-2	P0DTC1-1282	Y???L???SKTPE	VVLPSYEEALSLPSKTPE	YNGYLTSSSKTPEEH
H7C2W9-1	P0DTC2-137	VTYVP	VTYVPVTTFKNLQTVNVDEN	HVTYVPAQEKNF
H7C2W9-1	P0DTC2-212	VTYVP	VTYVPVTTFKNLQTVNVDEN	APHGVVFLHVTYVPAQEKNF
H7C2W9-1	P0DTC2-306	VTYVP	VTYVPVTTFKNLQTVNVDEN	APHGVVFLHVTYVPA
H7C2W9-1	P0DTC2-422	VTYVP	VTYVPVTTFKNLQTVNVDEN	VTYVPAQEK
H7C2W9-1	P0DTC2-565	VTYVP	VTYVPVTTFKNLQTVNVDEN	VTYVPAQEKNFTTAP
H7C2W9-1	P0DTC2-615	VTYVP	VTYVPVTTFKNLQTVNVDEN	HVTYVPAQEK
H7C2W9-3	P0DTC2-137	VTYVP	VTYVPVTTFK	HVTYVPAQEKNF
H7C2W9-3	P0DTC2-212	VTYVP	VTYVPVTTFK	APHGVVFLHVTYVPAQEKNF
H7C2W9-3	P0DTC2-306	VTYVP	VTYVPVTTFK	APHGVVFLHVTYVPA
H7C2W9-3	P0DTC2-422	VTYVP	VTYVPVTTFK	VTYVPAQEK
H7C2W9-3	P0DTC2-565	VTYVP	VTYVPVTTFK	VTYVPAQEKNFTTAP
H7C2W9-3	P0DTC2-615	VTYVP	VTYVPVTTFK	HVTYVPAQEK
P11586-1	P0DTD1-377	QVNGL	IDLDPETEQVNGLF	NHTKKWKYPQVNGLT
P11586-1	P0DTD1-461	QVNGL	IDLDPETEQVNGLF	YPQVNGLTSI
P11586-1	P0DTD1-543	QVNGL	IDLDPETEQVNGLF	KYPQVNGLTSIKWAD
P11586-1	P0DTC1-142	QVNGL	IDLDPETEQVNGLF	YPQVNGLTSIKWAD
P11586-1	P0DTC1-633	QVNGL	IDLDPETEQVNGLF	KKWKYPQVNGLTSIK
P11586-1	P0DTC1-749	QVNGL	IDLDPETEQVNGLF	LNHTKKWKYPQVNGL
P11586-1	P0DTC1-953	QVNGL	IDLDPETEQVNGLF	QVNGLTSIKWADNNC
P11586-1	P0DTC1-1245	QVNGL	IDLDPETEQVNGLF	WKYPQVNGLTSIKWA
P14174-2	P0DTD1-633	VPRAS	IVNTNVPRASVPDGFLSEL	HNKCAYWVPRAS
P62861-1	P0DTC1-202	ARAGK	KVHGSLARAGKVRGQTPK	ARAGKASCTLSEQLD
P62736-3	P0DTC1-501	EKSYE	SLEKSYELPDGQVI	HEIAWYTERSEKSYE
P37108-1	P0DTC5-36	LLESE	VLLESEQFLTELTRL	LLESELVIGAVILRGHLR
P37108-1	P0DTC5-64	LLESE	VLLESEQFLTELTRL	HGTILTRPLLESELV
P37108-1	P0DTC5-72	LLESE	VLLESEQFLTELTRL	LLESELVIGAVILRG
P37108-1	P0DTC5-76	LLESE	VLLESEQFLTELTRL	LTRPLLESELVIGAV
P37108-1	P0DTC5-97	LLESE	VLLESEQFLTELTRL	GTILTRPLLESELVI
P37108-1	P0DTC5-117	LLESE	VLLESEQFLTELTRL	RPLLESEL

P37108-1	P0DTC5-118	LLESE	VLLESEQFLTELTRL	RPLLESELVIGAVIL
P37108-1	P0DTC5-214	LLESE	VLLESEQFLTELTRL	PLHGTILTRPLLESE
P37108-1	P0DTC5-216	LLESE	VLLESEQFLTELTRL	TILTRPLLESELVIG
P14921	P0DTD1-298	FITES	EPSFITESY	EVKPFITESK
P14921	P0DTC1-165	FITES	EPSFITESY	AEIPKEEVKPFITES
P14921	P0DTC1-388	FITES	EPSFITESY	EVKPFITESKPSVEQ
P14921	P0DTC1-421	FITES	EPSFITESY	FITESKPSVEQRKQD
P14921	P0DTC1-566	FITES	EPSFITESY	IPKEEVKPFITESKP
P14921	P0DTC1-614	FITES	EPSFITESY	KEEVKPFITESKPSV
P14921	P0DTC1-643	FITES	EPSFITESY	KPFITESKPSVEQRK
P04150	P0DTD1-17	EVVEN	EVVENLLNY	EEVVENPTI
P04150	P0DTC1-120	EVVEN	EVVENLLNY	SEEVVENPTIQKDVLECN
P04150	P0DTC1-152	EVVEN	EVVENLLNY	ACEDLKPVSEEVVEN
P04150	P0DTC1-318	EVVEN	EVVENLLNY	EDLKPVSEEVVENPT
P04150	P0DTC1-396	EVVEN	EVVENLLNY	EVVENPTIQKDVLEC
P04150	P0DTC1-716	EVVEN	EVVENLLNY	LKPVSEEVVENPTIQ
P04150	P0DTC1-919	EVVEN	EVVENLLNY	PVSEEVVENPTIQKD
P04150	P0DTC1-997	EVVEN	EVVENLLNY	SEEVVENPTIQKDVL
Q06323-1	P0DTD1-625	DIILK	LYDIILKNF	GDIILKPANNSL
Q06323-1	P0DTC1-275	DIILK	LYDIILKNF	DIILKPANNSLKITE
Q06323-1	P0DTC1-397	DIILK	LYDIILKNF	EVVGDIILKPANNSL
Q06323-1	P0DTC1-1128	DIILK	LYDIILKNF	TTEVVGDIILKPANN
Q06323-1	P0DTC1-1196	DIILK	LYDIILKNF	VKTTEVVGDIILKPA
H7C2W9-4	P0DTC2-137	VTYVP	TLVTYVPVTTFK	HVTYVPAQEKNF
H7C2W9-4	P0DTC2-212	VTYVP	TLVTYVPVTTFK	APHGVVFLHVTYVPAQEKNF
H7C2W9-4	P0DTC2-306	VTYVP	TLVTYVPVTTFK	APHGVVFLHVTYVPA
H7C2W9-4	P0DTC2-422	VTYVP	TLVTYVPVTTFK	VTYVPAQEK
H7C2W9-4	P0DTC2-565	VTYVP	TLVTYVPVTTFK	VTYVPAQEKNFTTAP
H7C2W9-4	P0DTC2-615	VTYVP	TLVTYVPVTTFK	HVTYVPAQEK
P37108-2	P0DTC5-36	LLESE	VLLESEQFLTEL	LLESELVIGAVILRGHLR
P37108-2	P0DTC5-64	LLESE	VLLESEQFLTEL	HGTILTRPLLESELV
P37108-2	P0DTC5-72	LLESE	VLLESEQFLTEL	LLESELVIGAVILRG
P37108-2	P0DTC5-76	LLESE	VLLESEQFLTEL	LTRPLLESELVIGAV

P37108-2	P0DTC5-97	LLESE	VLLESEQFLTEL	GTILTRPLLESELVI
P37108-2	P0DTC5-117	LLESE	VLLESEQFLTEL	RPLLESEL
P37108-2	P0DTC5-118	LLESE	VLLESEQFLTEL	RPLLESELVIGAVIL
P37108-2	P0DTC5-214	LLESE	VLLESEQFLTEL	PLHGTILTRPLLESE
P37108-2	P0DTC5-216	LLESE	VLLESEQFLTEL	TILTRPLLESELVIG
H7C2W9-5	P0DTC2-137	VTYVP	VTYVPVTTF	HVTYVPAQEKNF
H7C2W9-5	P0DTC2-212	VTYVP	VTYVPVTTF	APHGVVFLHVTYVPAQEKNF
H7C2W9-5	P0DTC2-306	VTYVP	VTYVPVTTF	APHGVVFLHVTYVPA
H7C2W9-5	P0DTC2-422	VTYVP	VTYVPVTTF	VTYVPAQEK
H7C2W9-5	P0DTC2-565	VTYVP	VTYVPVTTF	VTYVPAQEKNFTTAP
H7C2W9-5	P0DTC2-615	VTYVP	VTYVPVTTF	HVTYVPAQEK
P05783-9	P0DTC1-80	LGSAL	SLGSALRP	GMNGRTILGSALLED
P05783-9	P0DTC1-560	LGSAL	SLGSALRP	ILGSALLEDEFTPFD
P05783-9	P0DTC1-839	LGSAL	SLGSALRP	NGRTILGSALLEDEF
P05783-9	P0DTC1-973	LGSAL	SLGSALRP	RTILGSALLEDEFTP
P05783-10	P0DTC1-80	LGSAL	SLGSALRPSTSRSL	GMNGRTILGSALLED
P05783-10	P0DTC1-560	LGSAL	SLGSALRPSTSRSL	ILGSALLEDEFTPFD
P05783-10	P0DTC1-839	LGSAL	SLGSALRPSTSRSL	NGRTILGSALLEDEF
P05783-10	P0DTC1-973	LGSAL	SLGSALRPSTSRSL	RTILGSALLEDEFTP
P62316	P0DTD1-132	EEEEF	EEEEFNTGP	GDCEEEEFEPSTQYEY
P62316	P0DTD1-612	EEEEF	EEEEFNTGP	EEEEFEPSTQYE
P62316	P0DTC1-234	EEEEF	EEEEFNTGP	CEEEEFEPSTQYEYG
P62316	P0DTC1-261	EEEEF	EEEEFNTGP	DEDEEEGDCEEEEFE
P62316	P0DTC1-262	EEEEF	EEEEFNTGP	DEEEGDCEEEEFEPS
P62316	P0DTC1-327	EEEEF	EEEEFNTGP	EEGDCEEEEFEPSTQ
P62316	P0DTC1-453	EEEEF	EEEEFNTGP	GDCEEEEFEPSTQYE
P47914-1	P0DTD1-890	TQAPT??S	AQAAAPASVPAQAPKRTQAPT KASE	GLHPTQAPTHLSVDT
P47914-1	P0DTD1-949	TQAPT??S	AQAAAPASVPAQAPKRTQAPT KASE	ITGLHPTQAPTHLSV
Q53YP1-1	P0DTC1-607	KDYLA	DGKDYLALNEDLRSW	KAYKDYLASGGQPIT
Q53YP1-1	P0DTC1-1267	KDYLA	DGKDYLALNEDLRSW	YKDYLASGGQPITNC
P08575-1	P0DTC1-1072	???KALRK	VDIFQVVKALRKA	TEMLAKALRKVPTDN

P08575-2	P0DTC1-1072	???KALRK	VVDIFQVVKALRKARPG	TEMLAKALRKVPTDN
Q9NQ90-1	P0DTC2-981	GETGK	GETGKEPHAGGPGDIELGPL	VRQIAPGETGKIADY
Q53YP1-2	P0DTC1-607	KDYLA	AYDGKDYLALNEDLRSWT	KAYKDYLASGGQPIT
Q53YP1-2	P0DTC1-1267	KDYLA	AYDGKDYLALNEDLRSWT	YKDYLASGGQPITNC
Q53YP1-3	P0DTC1-607	KDYLA	DGKDYLALNEDLR	KAYKDYLASGGQPIT
Q53YP1-3	P0DTC1-1267	KDYLA	DGKDYLALNEDLR	YKDYLASGGQPITNC
Q53YP1-4	P0DTC1-607	KDYLA	DGKDYLALNEDLRS	KAYKDYLASGGQPIT
Q53YP1-4	P0DTC1-1267	KDYLA	DGKDYLALNEDLRS	YKDYLASGGQPITNC
F1MPL6	P0DTC1-607	KDYLA	DGKDYLALNEDLRSWT	KAYKDYLASGGQPIT
F1MPL6	P0DTC1-1267	KDYLA	DGKDYLALNEDLRSWT	YKDYLASGGQPITNC
Q53YP1-5	P0DTC1-607	KDYLA	DGKDYLALNEDLRSWTA	KAYKDYLASGGQPIT
Q53YP1-5	P0DTC1-1267	KDYLA	DGKDYLALNEDLRSWTA	YKDYLASGGQPITNC
Q53YP1-6	P0DTC1-607	KDYLA	GKDYLALNEDLR	KAYKDYLASGGQPIT
Q53YP1-6	P0DTC1-1267	KDYLA	GKDYLALNEDLR	YKDYLASGGQPITNC
Q53YP1-7	P0DTC1-607	KDYLA	GKDYLALNEDLRSW	KAYKDYLASGGQPIT
Q53YP1-7	P0DTC1-1267	KDYLA	GKDYLALNEDLRSW	YKDYLASGGQPITNC
Q53YP1-8	P0DTC1-607	KDYLA	GKDYLALNEDLRSWTA	KAYKDYLASGGQPIT
Q53YP1-8	P0DTC1-1267	KDYLA	GKDYLALNEDLRSWTA	YKDYLASGGQPITNC
P05109-1	P0DTC1-832	LNSII	MLTELEKALNSIIDV	NFVFPLNSIIKTIQP
P05109-1	P0DTC1-1179	LNSII	MLTELEKALNSIIDV	VFPLNSIIKTIQPRV
P62937-2	P0DTD1-970	TVFFD	VNPTVFFDIAVDGEPLGRVS	KPTETICAPLTVFFD
P62937-2	P0DTD1-1098	TVFFD??VDG?	VNPTVFFDIAVDGEPLGRVS	PLTVFFDGRVDGQVD
P62937-2	P0DTD1-1197	TVFFD	VNPTVFFDIAVDGEPLGRVS	TETICAPLTVFFDGR
P62937-2	P0DTD1-1202	TVFFD??VD	VNPTVFFDIAVDGEPLGRVS	TICAPLTVFFDGRVD
P62937-2	P0DTD1-1234	TVFFD??VDG?	VNPTVFFDIAVDGEPLGRVS	TVFFDGRVDGQVDLF
Q53YP1-9	P0DTC1-607	KDYLA	YDGKDYLALNEDLRSWT	KAYKDYLASGGQPIT
Q53YP1-9	P0DTC1-1267	KDYLA	YDGKDYLALNEDLRSWT	YKDYLASGGQPITNC
H7C2W9-6	P0DTC2-137	VTYVP	TLVTYVPVTTF	HVTYVPAQEKNF
H7C2W9-6	P0DTC2-212	VTYVP	TLVTYVPVTTF	APHGVVFLHVTYVPAQEKNF
H7C2W9-6	P0DTC2-306	VTYVP	TLVTYVPVTTF	APHGVVFLHVTYVPA
H7C2W9-6	P0DTC2-422	VTYVP	TLVTYVPVTTF	VTYVPAQEK
H7C2W9-6	P0DTC2-565	VTYVP	TLVTYVPVTTF	VTYVPAQEKNFTTAP
H7C2W9-6	P0DTC2-615	VTYVP	TLVTYVPVTTF	HVTYVPAQEK

P06733-3	P0DTD1-638	ADLYK	ADLYKSFIKDYPVVS	ITEHSWNADLYKLM
P06733-3	P0DTD1-909	ADLYK	ADLYKSFIKDYPVVS	HSWNADLYKLMGHFA
P06733-3	P0DTD1-1287	ADLYK	ADLYKSFIKDYPVVS	WNADLYKLMGHFAWW
P29401-5	P0DTC1-505	YFDKA	CGMAYTGKYFDKASY	HLYFDKAGQKTYERH
P29401-5	P0DTC1-1015	YFDKA	CGMAYTGKYFDKASY	SIHLYFDKAGQKTYE
P29401-5	P0DTC1-1195	YFDKA	CGMAYTGKYFDKASY	VKNGSIHLYFDKAGQ
P29401-5	P0DTC1-1232	YFDKA	CGMAYTGKYFDKASY	VTVKNGSIHLYFDKA
P29401-5	P0DTC1-1260	YFDKA	CGMAYTGKYFDKASY	YFDKAGQKTYERHSL
Q05329-2	P0DTD1-37	Q??LKYAI	CQTTLKYAIKTGHPR	IPTITQMNLKYAISA
Q05329-2	P0DTD1-548	QTTLK	CQTTLKYAIKTGHPR	NVVCKTCGQQQTTLK
Q05329-2	P0DTD1-1056	?LKYAI	CQTTLKYAIKTGHPR	MNLKYAISAKNRART
Q05329-2	P0DTD1-1204	Q??LKYAI	CQTTLKYAIKTGHPR	TITQMNLKYAISAKN
Q05329-2	P0DTD1-1220	Q??LKYAI	CQTTLKYAIKTGHPR	TQMNLKYAISAKNRA
Q05329-2	P0DTC1-238	QTTLK	CQTTLKYAIKTGHPR	CKTCGQQQTTLKGVE
Q05329-2	P0DTC1-482	QTTLK	CQTTLKYAIKTGHPR	GQQQTTLKGVEAVMY
Q05329-2	P0DTC1-1060	QTTLK	CQTTLKYAIKTGHPR	TCGQQQTTLKGVEAV
Q05329-2	P0DTC1-1235	QTTLK	CQTTLKYAIKTGHPR	VVCKTCGQQQTTLKG
P02489	P0DTD1-776	DDFVE	DDFVEIHGKHNERQD	CVCSVIDLLLDDFVE
A0A0D9SGF6-3	P0DTC1-149	RQGFV	DWWKVEVNDRQGFVP	AARQGFVDSDVETKD
A0A0D9SGF6-3	P0DTC1-576	RQGFV	DWWKVEVNDRQGFVP	ISAARQGFVDSDVET
A0A0D9SGF6-3	P0DTC1-772	RQGFV	DWWKVEVNDRQGFVP	LSTFISAARQGFVDS
A0A0D9SGF6-3	P0DTC1-970	RQGFV	DWWKVEVNDRQGFVP	RQGFVDSDVETKDVV
A0A0D9SGF6-3	P0DTC1-1082	RQGFV	DWWKVEVNDRQGFVP	TFISAARQGFVDSDV
P09972-3	P0DTD1-338	EASFN	EASFNLNAINRCPLP	KFCLEASFNY
P09972-3	P0DTD1-476	EASFN	EASFNLNAINRCPLP	CLEASFNYL
P09972-3	P0DTC1-95	EASFN	EASFNLNAINRCPLP	LEASFNYLKSPNFS
P09972-3	P0DTC1-310	EASFN	EASFNLNAINRCPLP	EASFNYLKSPNFSKL
A0A1W2PRT3-1	P0DTD1-568	DGEVI	EMRDGEVIKESKQEH	LDGEVITFDNLKTLLS
A0A1W2PRT3-1	P0DTC1-417	DGEVI	EMRDGEVIKESKQEH	FHLDGEVITFDNLKT
A0A1W2PRT3-1	P0DTC1-678	DGEVI	EMRDGEVIKESKQEH	LDGEVITFDNLKTLL
A0A1W2PRT3-1	P0DTC1-868	DGEVI	EMRDGEVIKESKQEH	NPTTFHLDGEVITFD
A0A1W2PRT3-1	P0DTC1-1125	DGEVI	EMRDGEVIKESKQEH	TSNPTTFHLDGEVIT
A0A1W2PRT3-1	P0DTC1-1129	DGEVI	EMRDGEVIKESKQEH	TTFHLDGEVITFDNL

P08238-5	P0DTD1-789	?LFENK	FIPRRAPFDLFENKK	DGVDVELFENKTTLP
P08238-5	P0DTD1-992	LFENK	FIPRRAPFDLFENKK	LFENKTTLPVNVAFE
P08238-5	P0DTD1-1246	?LFENK	FIPRRAPFDLFENKK	VDVELFENKTTLPVN
P08238-5	P0DTD1-1312	?LFENK	FIPRRAPFDLFENKK	YTKVDGVDVELFENK
P29401-7	P0DTD1-255	AEAEL	FLAEAELLNLRKISS	AEAELAKNV
P29401-7	P0DTC1-129	AEAEL	FLAEAELLNLRKISS	TLVATAEAELAKNVSL
P29401-7	P0DTC1-212	AEAEL	FLAEAELLNLRKISS	ATAEAELAKNVSLDN
P29401-7	P0DTC1-787	AEAEL	FLAEAELLNLRKISS	LVATAEAELAKNVSL
P29401-7	P0DTC1-802	AEAEL	FLAEAELLNLRKISS	MEKLKTLVATAEAEL
P08779-10	P0DTC1-174	AGALN	FSLAGALNAGFKETR	AGALNKATNNAMQVE
P08779-10	P0DTC1-464	??AGALN	FSLAGALNAGFKETR	GGGVAGALNKATNNA
P08779-10	P0DTC1-492	??AGALN	FSLAGALNAGFKETR	GVAGALNKATNNAMQ
P08779-10	P0DTC1-626	??AGALN	FSLAGALNAGFKETR	KHGGGVAGALNKATN
P08238-9	P0DTC1-31	????SAFVE	GESKEQVANSAFVER	ILASFSASTSAFVET
P08238-9	P0DTC1-208	????SAFVE	GESKEQVANSAFVER	ASFSASTSAFVETVK
P08238-9	P0DTC1-211	???SAFVE	GESKEQVANSAFVER	ASTSAFVETVKGLDY
P02511-7	P0DTD1-940	IRRPF	HPWIRRPFFPFHSPS	IRRPFLCCKCCYDHV
P02511-7	P0DTD1-1174	IRRPF	HPWIRRPFFPFHSPS	SQTSLRCGACIRRPF
P02511-7	P0DTD1-1229	IRRPF	HPWIRRPFFPFHSPS	TSLRCGACIRRPFLC
A0A1W2PRT3-2	P0DTD1-568	DGEVI	IVVKTVEMRDGEVIK	LDGEVITFDNLKTLLS
A0A1W2PRT3-2	P0DTC1-417	DGEVI	IVVKTVEMRDGEVIK	FHLDGEVITFDNLKT
A0A1W2PRT3-2	P0DTC1-678	DGEVI	IVVKTVEMRDGEVIK	LDGEVITFDNLKTLL
A0A1W2PRT3-2	P0DTC1-868	DGEVI	IVVKTVEMRDGEVIK	NPTTFHLDGEVITFD
A0A1W2PRT3-2	P0DTC1-1125	DGEVI	IVVKTVEMRDGEVIK	TSNPTTFHLDGEVIT
A0A1W2PRT3-2	P0DTC1-1129	DGEVI	IVVKTVEMRDGEVIK	TTFHLDGEVITFDNL
Q13813-16	P0DTC1-505	??KAGQK	LEKAGQKLIDVNHYA	HLYFDKAGQKTYERH
Q13813-16	P0DTC1-1015	??KAGQK	LEKAGQKLIDVNHYA	SIHLYFDKAGQKTYE
Q13813-16	P0DTC1-1260	??KAGQK	LEKAGQKLIDVNHYA	YFDKAGQKTYERHSL
O75508-9	P0DTC1-618	?LALCA	LILLALCALVATIWF	KFLALCADSIIIGGA
O75508-9	P0DTC1-667	LALCA	LILLALCALVATIWF	LALCADSIIIGGAKL
O75508-9	P0DTC1-1206	?LALCA	LILLALCALVATIWF	VNKFLALCADSIIIG
P02511-11	P0DTD1-940	IRRPF	MDIAIHHPWIRRPFF	IRRPFLCCKCCYDHV
P02511-11	P0DTD1-1174	IRRPF	MDIAIHHPWIRRPFF	SQTSLRCGACIRRPF

P02511-11	P0DTD1-1229	IRRPF	MDIAIHHPWIRRPFF	TSLRCGACIRRPFLC
P08238-16	P0DTD1-789	?LFENK	PFDLFENKKKKNNIK	DGVDVELFENKTTLP
P08238-16	P0DTD1-992	LFENK	PFDLFENKKKKNNIK	LFENKTTLPVNVAFE
P08238-16	P0DTD1-1246	?LFENK	PFDLFENKKKKNNIK	VDVELFENKTTLPVN
P08238-16	P0DTD1-1312	?LFENK	PFDLFENKKKKNNIK	YTKVDGVDVELFENK
P07196-13	P0DTC1-333	EEVLS	QARYEEEVLSREDAE	EEVLSEARQHLKDGT
P07196-13	P0DTC1-461	EEVLS	QARYEEEVLSREDAE	GFGDSVEEVLSEARQ
P07196-13	P0DTC1-1204	EEVLS	QARYEEEVLSREDAE	VLVRGFGDSVEEVLS
P07196-13	P0DTC1-1219	EEVLS	QARYEEEVLSREDAE	VRGFGDSVEEVLSEA
Q13813-24	P0DTC1-505	??KAGQK	QSRIDALEKAGQKLI	HLYFDKAGQKTYERH
Q13813-24	P0DTC1-1015	??KAGQK	QSRIDALEKAGQKLI	SIHLYFDKAGQKTYE
Q13813-24	P0DTC1-1260	??KAGQK	QSRIDALEKAGQKLI	YFDKAGQKTYERHSL
P09543-16	P0DTD1-274	??GKPVP	RAIFTGYYGKGKPVP	DASGKPVPY
P09543-16	P0DTC1-256	??GKPVP	RAIFTGYYGKGKPVP	DASGKPVPYCYDTNV
P09543-16	P0DTC1-314	??GKPVP	RAIFTGYYGKGKPVP	ECTIFKDASGKPVPY
P09543-16	P0DTC1-1010	?GKPVP	RAIFTGYYGKGKPVP	SGKPVPYCYDTNVLE
Q9BZR6-16	P0DTC2-29	LQELG	RCGLQELGPGLFRGL	SLIDLQELGKYEQYIKW
Q9BZR6-16	P0DTC2-61	LQELG	RCGLQELGPGLFRGL	ESLIDLQELGKYEQY
Q9BZR6-16	P0DTC2-202	LQELG	RCGLQELGPGLFRGL	LIDLQELGKYEQYI
Q9BZR6-16	P0DTC2-246	LQELG	RCGLQELGPGLFRGL	LNEVAKNLNESLIDLQELGK
Q9BZR6-16	P0DTC2-277	LQELG	RCGLQELGPGLFRGL	SLIDLQELGKYEQYIKWPWY
Q9BZR6-16	P0DTC2-509	LQELG	RCGLQELGPGLFRGL	LNESLIDLQELGKYE
P10809-15	P0DTD1-715	KGVIT	SDAMKKVGRKGVITV	YKGVITHDVSSAIN
P10809-15	P0DTD1-1111	K???KGVIT	SDAMKKVGRKGVITV	QCFKMFYKGVITHDV
P23297-4	P0DTD1-795	VAALT	VAALTVACNNFFWEN	DKRTTCFSVAALTNN
P23297-4	P0DTD1-1012	VAALT	VAALTVACNNFFWEN	LLDKRTTCFSVAALT
P23297-4	P0DTD1-1150	VAALT	VAALTVACNNFFWEN	RTTCFSVAALTNNVA
P23297-4	P0DTD1-1189	VAALT	VAALTVACNNFFWEN	TCFSVAALTNNVAFQ
P08238-20	P0DTC1-31	???SAFVE	VANSAFVERVRKRGF	ILASFSASTSAFVET
P08238-20	P0DTC1-208	???SAFVE?V?	VANSAFVERVRKRGF	ASFSASTSAFVETVK
P08238-20	P0DTC1-211	???SAFVE?V?	VANSAFVERVRKRGF	ASTSAFVETVKGLDY
P09972-8	P0DTC5-13	VLAAV	VLAAVYKALSDHHVY	TLACFVLAAV
P09972-8	P0DTC5-22	VLAAV	VLAAVYKALSDHHVY	FLWLLWPVTLACFVLAAV

P09972-8	P0DTC5-101	LAAVY?	VLAAVYKALSDHHVY	LAAVYRINW
P09972-8	P0DTC5-105	VLAAV	VLAAVYKALSDHHVY	LLWPVTLACFVLAAV
P29401-26	P0DTD1-255	AEAEL	VWAEAGFLAEAELLN	AEAELAKNV
P29401-26	P0DTC1-129	AEAEL	VWAEAGFLAEAELLN	TLVATAEAELAKNVSL
P29401-26	P0DTC1-212	AEAEL	VWAEAGFLAEAELLN	ATAEAELAKNVSLDN
P29401-26	P0DTC1-787	AEAEL	VWAEAGFLAEAELLN	LVATAEAELAKNVSL
P29401-26	P0DTC1-802	AEAEL	VWAEAGFLAEAELLN	MEKLKTLVATAEAEL
P09972-9	P0DTC1-872	VTALR	YTPEEIAMATVTALR	NSPNLAWPLIVTALR
P09972-9	P0DTC1-903	VTALR	YTPEEIAMATVTALR	PNLAWPLIVTALRAN
P09543-19	P0DTD1-274	??GKPVP	YYGKGKPVPTQGSRK	DASGKPVPY
P09543-19	P0DTC1-256	??GKPVP	YYGKGKPVPTQGSRK	DASGKPVPYCYDTNV
P09543-19	P0DTC1-314	??GKPVP	YYGKGKPVPTQGSRK	ECTIFKDASGKPVPY
P09543-19	P0DTC1-1010	?GKPVP	YYGKGKPVPTQGSRK	SGKPVPYCYDTNVLE
P01042-2	P0DTC1-102	AVDAA	DKDLFKAVDAALKK	LSFCAFAVDAAKAY
P05783-19	P0DTC9-332	G???AS??SSSRS	FGGPGTASRPSSSRS	LPQGTTLPKGFYAEGSRGGSQASSRSSSRSRNSSRNSTP
P05783-19	P0DTC9-441	AS??SSSRS	FGGPGTASRPSSSRS	ASSRSSSRSRNSSRN
P05783-19	P0DTC9-454	G???AS??SSSRS	FGGPGTASRPSSSRS	GGSQASSRSSSRSRN
P05783-19	P0DTC9-501	G???AS??SSSRS	FGGPGTASRPSSSRS	SRGGSQASSRSSSRS
P05783-19	P0DTC9-502	SSSRS	FGGPGTASRPSSSRS	SRSSSRSRNSSRNST
P01042-3	P0DTC1-102	AVDAA	NDKDLFKAVDAALKK	LSFCAFAVDAAKAY
P05783-20	P0DTC1-80	LGSAL	SLGSALRPSTSRS	GMNGRTILGSALLED
P05783-20	P0DTC1-560	LGSAL	SLGSALRPSTSRS	ILGSALLEDEFTPFD
P05783-20	P0DTC1-839	LGSAL	SLGSALRPSTSRS	NGRTILGSALLEDEF
P05783-20	P0DTC1-973	LGSAL	SLGSALRPSTSRS	RTILGSALLEDEFTP
P05783-21	P0DTC1-80	LGSAL	SLGSALRPSTSRSLY	GMNGRTILGSALLED
P05783-21	P0DTC1-560	LGSAL	SLGSALRPSTSRSLY	ILGSALLEDEFTPFD
P05783-21	P0DTC1-839	LGSAL	SLGSALRPSTSRSLY	NGRTILGSALLEDEF
P05783-21	P0DTC1-973	LGSAL	SLGSALRPSTSRSLY	RTILGSALLEDEFTP
P62736-6	P0DTC1-501	EKSYE	SSSSLEKSYELPDGQVI	HEIAWYTERSEKSYE
Q16695-3	P0DTC8-7	ARKSA	VARKSAPATGGVKKPHRYRPG TVAL	FYSKWYIRVGARKSA
P01042-4	P0DTC1-102	AVDAA	DKDLFKAVDAALKKYN	LSFCAFAVDAAKAY
A0A0C4DGB6-15	P0DTC1-34	TFLKK	DNEETFLKKYLYEIARRHP	ITFLKKDAPYIVGDV

A0A0C4DGB6-15	P0DTC1-214	D???TFLKK	DNEETFLKKYLYEIARRHP	ATLVSDIDITFLKKD
A0A0C4DGB6-15	P0DTC1-535	TFLKK	DNEETFLKKYLYEIARRHP	IDITFLKKDAPYIVG
A0A0C4DGB6-15	P0DTC1-791	D???TFLKK	DNEETFLKKYLYEIARRHP	LVSDIDITFLKKDAP
A0A0C4DGB6-15	P0DTC1-988	D???TFLKK	DNEETFLKKYLYEIARRHP	SDIDITFLKKDAPYI
P01042-5	P0DTC1-102	AVDAA	NDKDLFKAVDAALKKYN	LSFCAFAVDAAKAY
P11586-2	P0DTD1-377	QVNGL	FYDIDLDPETEQVNGLF	NHTKKWKYPQVNGLT
P11586-2	P0DTD1-461	QVNGL	FYDIDLDPETEQVNGLF	YPQVNGLTSI
P11586-2	P0DTD1-543	QVNGL	FYDIDLDPETEQVNGLF	KYPQVNGLTSIKWAD
P11586-2	P0DTC1-142	QVNGL	FYDIDLDPETEQVNGLF	YPQVNGLTSIKWAD
P11586-2	P0DTC1-633	QVNGL	FYDIDLDPETEQVNGLF	KKWKYPQVNGLTSIK
P11586-2	P0DTC1-749	QVNGL	FYDIDLDPETEQVNGLF	LNHTKKWKYPQVNGL
P11586-2	P0DTC1-953	QVNGL	FYDIDLDPETEQVNGLF	QVNGLTSIKWADNNC
P11586-2	P0DTC1-1245	QVNGL	FYDIDLDPETEQVNGLF	WKYPQVNGLTSIKWA
Q86V81-2	P0DTD1-51	LDAYN	SAEELDAQLDAYNARMDTS	LDAYNMMISAGFSLW
Q86V81-2	P0DTD1-119	LDAYN	SAEELDAQLDAYNARMDTS	YLDAYNMMI
Q86V81-2	P0DTD1-902	A?E????LDAYN	SAEELDAQLDAYNARMDTS	HANEYRLYLDAYNMM
Q86V81-2	P0DTD1-1045	LDAYN	SAEELDAQLDAYNARMDTS	LYLDAYNMMISAGFS
Q86V81-2	P0DTD1-1135	A?E????LDAYN	SAEELDAQLDAYNARMDTS	RHHANEYRLYLDAYN
P08575-3	P0DTC1-1072	???KALRK	DIFQVVKALRK	TEMLAKALRKVPTDN
P08575-4	P0DTC1-1072	???KALRK	DIFQVVKALRKA	TEMLAKALRKVPTDN
P08575-5	P0DTC1-1072	???KALRK	DIFQVVKALRKARP	TEMLAKALRKVPTDN
P08575-6	P0DTC1-1072	???KALRK	DIFQVVKALRKARPG	TEMLAKALRKVPTDN
Q00653	P0DTC1-265	DFKLN	NPGLDGIIEYDDFKLNSS	DFKLNEEIAIILASF
Q00653	P0DTC1-661	DFKLN	NPGLDGIIEYDDFKLNSS	KVNINIVGDFKLNEE
Q00653	P0DTC1-848	DFKLN	NPGLDGIIEYDDFKLNSS	NINIVGDFKLNEEIA
Q00653	P0DTC1-851	DFKLN	NPGLDGIIEYDDFKLNSS	NIVGDFKLNEEIAII
Q00653	P0DTC1-1182	DFKLN	NPGLDGIIEYDDFKLNSS	VGDFKLNEEIAIILA
P08575-7	P0DTC1-1072	???KALRK	VDIFQVVKALRKARP	TEMLAKALRKVPTDN
P08575-8	P0DTC1-1072	???KALRK	VDIFQVVKALRKARPG	TEMLAKALRKVPTDN
P08575-9	P0DTC1-1072	???KALRK	VVDIFQVVKALRKA	TEMLAKALRKVPTDN
P08575-10	P0DTC1-1072	???KALRK	VVDIFQVVKALRKARP	TEMLAKALRKVPTDN
I3L0Y5-1	P0DTC2-13	?GAGAA	DIDTAAKFIGAGAATVGVAG	GWTFGAGAALQIPFA
I3L0Y5-1	P0DTC2-41	GAGAA	DIDTAAKFIGAGAATVGVAG	GAGAALQIPFAMQMA

I3L0Y5-1	P0DTC2-400	T?????GAGAA	DIDTAAKFIGAGAATVGVAG	TITSGWTFGAGAALQ
I3L0Y5-1	P0DTC2-684	?GAGAA	DIDTAAKFIGAGAATVGVAG	WTFGAGAAL
I3L0Y5-1	P0DTC2-965	?GAGAA	DIDTAAKFIGAGAATVGVAG	FGAGAALQIPFAMQM
K7ERT8-2	P0DTC8-3	RKSAP	RRQPKYPRKSAPR	RVGARKSAPL
K7ERT8-2	P0DTC8-9	RKSAP	RRQPKYPRKSAPR	SKWYIRVGARKSAPL
K7ERT8-2	P0DTC8-11	RKSAP	RRQPKYPRKSAPR	YIRVGARKSAPLIEL
K7ERT8-2	P0DTC8-12	RKSAP	RRQPKYPRKSAPR	ARKSAPLIELCVDEA
K7ERT8-2	P0DTC8-68	RKSAP	RRQPKYPRKSAPR	GARKSAPLIELCVDE
K7ERT8-2	P0DTC8-82	RKSAP	RRQPKYPRKSAPR	RVGARKSAPLIELCV
P05783-23	P0DTC1-80	LGSAL	SLGSALRPSTS	GMNGRTILGSALLED
P05783-23	P0DTC1-560	LGSAL	SLGSALRPSTS	ILGSALLEDEFTPFD
P05783-23	P0DTC1-839	LGSAL	SLGSALRPSTS	NGRTILGSALLEDEF
P05783-23	P0DTC1-973	LGSAL	SLGSALRPSTS	RTILGSALLEDEFTP
H7C2W9-7	P0DTC2-137	VTYVP	SPNKLYTLVTYVPVTTF	HVTYVPAQEKNF
H7C2W9-7	P0DTC2-212	????VTYVP	SPNKLYTLVTYVPVTTF	APHGVVFLHVTYVPAQEKNF
H7C2W9-7	P0DTC2-306	????VTYVP	SPNKLYTLVTYVPVTTF	APHGVVFLHVTYVPA
H7C2W9-7	P0DTC2-422	VTYVP	SPNKLYTLVTYVPVTTF	VTYVPAQEK
H7C2W9-7	P0DTC2-565	VTYVP	SPNKLYTLVTYVPVTTF	VTYVPAQEKNFTTAP
H7C2W9-7	P0DTC2-615	VTYVP	SPNKLYTLVTYVPVTTF	HVTYVPAQEK
Q5RI18-3	P0DTC5-21	EE??KLLEQ?	VELQKEEAQKLLEQY	EELKKLLEQWNLVIGFLF
Q5RI18-3	P0DTC5-35	KLLEQ?	VELQKEEAQKLLEQY	KLLEQWNLV
Q5RI18-3	P0DTC5-39	EE??KLLEQ	VELQKEEAQKLLEQY	MADSNGTITVEELKKLLEQ
Q5RI18-3	P0DTC5-71	?KLLEQ?	VELQKEEAQKLLEQY	LKKLLEQWNLVIGFL
Q5RI18-3	P0DTC5-79	EE??KLLEQ	VELQKEEAQKLLEQY	NGTITVEELKKLLEQ
Q5RI18-3	P0DTC5-93	EE??KLLEQ?	VELQKEEAQKLLEQY	EELKKLLEQWNLVIG
Q5RI18-3	P0DTC5-158	EE??KLLEQ?	VELQKEEAQKLLEQY	GTITVEELKKLLEQWN
Q5RI18-3	P0DTC5-163	EE??KLLEQ?	VELQKEEAQKLLEQY	ITVEELKKLLEQWNLV
Q5RI18-3	P0DTC5-173	EE??KLLEQ?	VELQKEEAQKLLEQY	MADSNGTITVEELKKLLEQWNLVI
Q5RI18-3	P0DTC5-174	EE??KLLEQ?	VELQKEEAQKLLEQY	NGTITVEELKKLLEQW
Q5RI18-3	P0DTC5-180	EE??KLLEQ	VELQKEEAQKLLEQY	SNGTITVEELKKLLEQ
Q5RI18-3	P0DTC5-185	EE??KLLEQ?	VELQKEEAQKLLEQY	TITVEELKKLLEQWNL
Q5RI18-3	P0DTC5-188	EE??KLLEQ?	VELQKEEAQKLLEQY	TVEELKKLLEQWNLVI
Q5RI18-3	P0DTC5-201	EE??KLLEQ?	VELQKEEAQKLLEQY	MADSNGTITVEELKKLLEQWN

O43318-1	P0DTC2-68	?TNTSN?	ASTNTSNKSDTN	GTNTSNQVAVLYQDV
O43318-1	P0DTC2-116	?TNTSN?	ASTNTSNKSDTN	VSVITPGTNTSNQVA
O43318-1	P0DTC2-232	?TNTSN?	ASTNTSNKSDTN	GVSVITPGTNTSNQVAVLYQ
O43318-1	P0DTC2-480	?TNTSN?	ASTNTSNKSDTN	GGVSVITPGTNTSNQ
O43318-1	P0DTC2-561	?TNTSN?	ASTNTSNKSDTN	VITPGTNTSNQVAVL
O43318-1	P0DTC2-1048	?TNTSN?	ASTNTSNKSDTN	ITPGTNTSNQVA
O43318-1	P0DTC2-1262	?TNTSN?	ASTNTSNKSDTN	TPGTNTSNQVAVLYQ
O43318-2	P0DTC2-68	?TNTSN?	DIASTNTSNKSD	GTNTSNQVAVLYQDV
O43318-2	P0DTC2-116	?TNTSN?	DIASTNTSNKSD	VSVITPGTNTSNQVA
O43318-2	P0DTC2-232	?TNTSN?	DIASTNTSNKSD	GVSVITPGTNTSNQVAVLYQ
O43318-2	P0DTC2-480	?TNTSN?	DIASTNTSNKSD	GGVSVITPGTNTSNQ
O43318-2	P0DTC2-561	?TNTSN?	DIASTNTSNKSD	VITPGTNTSNQVAVL
O43318-2	P0DTC2-1048	?TNTSN?	DIASTNTSNKSD	ITPGTNTSNQVA
O43318-2	P0DTC2-1262	?TNTSN?	DIASTNTSNKSD	TPGTNTSNQVAVLYQ
O43318-2	P0DTC1-533	DIAST?T	DIASTNTSNKSD	IDGGVTRDIASTDTC
O43318-3	P0DTC2-68	?TNTSN?	FMDIASTNTSNK	GTNTSNQVAVLYQDV
O43318-3	P0DTC2-116	?TNTSN?	FMDIASTNTSNK	VSVITPGTNTSNQVA
O43318-3	P0DTC2-232	?TNTSN?	FMDIASTNTSNK	GVSVITPGTNTSNQVAVLYQ
O43318-3	P0DTC2-480	?TNTSN?	FMDIASTNTSNK	GGVSVITPGTNTSNQ
O43318-3	P0DTC2-561	?TNTSN?	FMDIASTNTSNK	VITPGTNTSNQVAVL
O43318-3	P0DTC2-1048	?TNTSN?	FMDIASTNTSNK	ITPGTNTSNQVA
O43318-3	P0DTC2-1262	?TNTSN?	FMDIASTNTSNK	TPGTNTSNQVAVLYQ
O43318-3	P0DTC1-533	DIAST?T	FMDIASTNTSNK	IDGGVTRDIASTDTC
Q7L8L6	P0DTD1-532	TSSSK	GLEFSKTSSSKA	DAVTAYNGYLTSSSK
Q7L8L6	P0DTC1-123	TSSSK	GLEFSKTSSSKA	TAYNGYLTSSSKTP
Q7L8L6	P0DTC1-223	TSSSK	GLEFSKTSSSKA	AVTAYNGYLTSSSKT
Q7L8L6	P0DTC1-497	TSSSK	GLEFSKTSSSKA	GYLTSSSKTPEEHFI
Q7L8L6	P0DTC1-784	TSSSK	GLEFSKTSSSKA	LTSSSKTPEEHFIET
Q7L8L6	P0DTC1-1057	TSSSK	GLEFSKTSSSKA	TAYNGYLTSSSKTPE
Q7L8L6	P0DTC1-1282	TSSSK	GLEFSKTSSSKA	YNGYLTSSSKTPEEH
Q15052	P0DTC1-398	F?ITGNT	GNDCTFEITGNT	EYCPIFFITGNTLQC
Q15052	P0DTC1-541	F?ITGNT	GNDCTFEITGNT	IFFITGNTLQCIMLV
O43318-4	P0DTC2-68	?TNTSN?	IASTNTSNKSDT	GTNTSNQVAVLYQDV

O43318-4	P0DTC2-116	?TNTSN?	IASTNTSNKSDT	VSVITPGTNTSNQVA
O43318-4	P0DTC2-232	?TNTSN?	IASTNTSNKSDT	GVSVITPGTNTSNQVAVLYQ
O43318-4	P0DTC2-480	?TNTSN?	IASTNTSNKSDT	GGVSVITPGTNTSNQ
O43318-4	P0DTC2-561	?TNTSN?	IASTNTSNKSDT	VITPGTNTSNQVAVL
O43318-4	P0DTC2-1048	?TNTSN?	IASTNTSNKSDT	ITPGTNTSNQVA
O43318-4	P0DTC2-1262	?TNTSN?	IASTNTSNKSDT	TPGTNTSNQVAVLYQ
P25440	P0DTC1-538	KKGAK	KKGAKLAALQGS	IDYKHYTPSFKKGAK
P57073	P0DTC9-106	APSAS	KSAPSASASPTE	YKHWPQIAQFAPSAS
P57073	P0DTC9-405	PSASA	KSAPSASASPTE	PSASAFFGMSRIGMEVTPS
P11161-1	P0DTC9-3	APSAS	KSSAPSASVPA	APSASAFFGM
P11161-1	P0DTC9-4	APSAS	KSSAPSASVPA	AQFAPSASA
P11161-1	P0DTC9-5	APSAS	KSSAPSASVPA	AQFAPSASAFFGMSR
P11161-1	P0DTC9-38	APSAS	KSSAPSASVPA	FAPSASAFF
P11161-1	P0DTC9-73	APSAS	KSSAPSASVPA	WPQIAQFAPSASAFF
P11161-1	P0DTC9-103	APSAS	KSSAPSASVPA	QFAPSASAFFGMSRI
P11161-1	P0DTC9-106	APSAS	KSSAPSASVPA	YKHWPQIAQFAPSAS
P11161-1	P0DTC9-107	APSAS	KSSAPSASVPA	AQFAPSASAF
P11161-1	P0DTC9-178	APSAS	KSSAPSASVPA	QFAPSASAFF
P11161-1	P0DTC9-440	APSAS	KSSAPSASVPA	APSASAFFGMSRIGM
P11161-1	P0DTC9-461	APSAS	KSSAPSASVPA	IAQFAPSASAFFGMS
Q75MD7-2	P0DTC2-13	?GAGAA	LGAGAAGGGGAG	GWTFGAGAALQIPFA
Q75MD7-2	P0DTC2-41	GAGAA	LGAGAAGGGGAG	GAGAALQIPFAMQMA
Q75MD7-2	P0DTC2-400	?GAGAA	LGAGAAGGGGAG	TITSGWTFGAGAALQ
Q75MD7-2	P0DTC2-684	?GAGAA	LGAGAAGGGGAG	WTFGAGAAL
Q75MD7-2	P0DTC2-965	?GAGAA	LGAGAAGGGGAG	FGAGAALQIPFAMQM
O43318-5	P0DTC2-68	?TNTSN?	MDIASTNTSNKS	GTNTSNQVAVLYQDV
O43318-5	P0DTC2-116	?TNTSN?	MDIASTNTSNKS	VSVITPGTNTSNQVA
O43318-5	P0DTC2-232	?TNTSN?	MDIASTNTSNKS	GVSVITPGTNTSNQVAVLYQ
O43318-5	P0DTC2-480	?TNTSN?	MDIASTNTSNKS	GGVSVITPGTNTSNQ
O43318-5	P0DTC2-561	?TNTSN?	MDIASTNTSNKS	VITPGTNTSNQVAVL
O43318-5	P0DTC2-1048	?TNTSN?	MDIASTNTSNKS	ITPGTNTSNQVA
O43318-5	P0DTC2-1262	?TNTSN?	MDIASTNTSNKS	TPGTNTSNQVAVLYQ
O43318-5	P0DTC1-533	DIAST?T	MDIASTNTSNKS	IDGGVTRDIASTDTC

Q9ULK6	P0DTC2-13	GAGAA	PDPGAGAAGGGG	GWTFGAGAALQIPFA
Q9ULK6	P0DTC2-41	GAGAA	PDPGAGAAGGGG	GAGAALQIPFAMQMA
Q9ULK6	P0DTC2-400	GAGAA	PDPGAGAAGGGG	TITSGWTFGAGAALQ
Q9ULK6	P0DTC2-684	GAGAA	PDPGAGAAGGGG	WTFGAGAAL
Q9ULK6	P0DTC2-965	GAGAA	PDPGAGAAGGGG	FGAGAALQIPFAMQM
Q6ZV29	P0DTD1-351	KKPAS	QGPSKKPASA	LNQLTGYKKPASREL
Q6ZV29	P0DTC1-53	KKPAS	QGPSKKPASA	GYKKPASRELKVTFF
Q6ZV29	P0DTC1-159	KKPAS	QGPSKKPASA	ADDLNQLTGYKKPAS
Q6ZV29	P0DTC1-283	KKPAS	QGPSKKPASA	DLNQLTGYKKPASRE
O43318-6	P0DTC2-68	?TNTSN	SFMDIASTNTSN	GTNTSNQVAVLYQDV
O43318-6	P0DTC2-116	?TNTSN	SFMDIASTNTSN	VSVITPGTNTSNQVA
O43318-6	P0DTC2-232	?TNTSN	SFMDIASTNTSN	GVSVITPGTNTSNQVAVLYQ
O43318-6	P0DTC2-480	?TNTSN	SFMDIASTNTSN	GGVSVITPGTNTSNQ
O43318-6	P0DTC2-561	?TNTSN	SFMDIASTNTSN	VITPGTNTSNQVAVL
O43318-6	P0DTC2-1048	?TNTSN	SFMDIASTNTSN	ITPGTNTSNQVA
O43318-6	P0DTC2-1262	?TNTSN	SFMDIASTNTSN	TPGTNTSNQVAVLYQ
O43318-6	P0DTC1-533	DIAST?T	SFMDIASTNTSN	IDGGVTRDIASTDTC
O43318-7	P0DTC2-68	?TNTSN?	STNTSNKSDTNM	GTNTSNQVAVLYQDV
O43318-7	P0DTC2-116	?TNTSN?	STNTSNKSDTNM	VSVITPGTNTSNQVA
O43318-7	P0DTC2-232	?TNTSN?	STNTSNKSDTNM	GVSVITPGTNTSNQVAVLYQ
O43318-7	P0DTC2-480	?TNTSN?	STNTSNKSDTNM	GGVSVITPGTNTSNQ
O43318-7	P0DTC2-561	?TNTSN?	STNTSNKSDTNM	VITPGTNTSNQVAVL
O43318-7	P0DTC2-1048	?TNTSN?	STNTSNKSDTNM	ITPGTNTSNQVA
O43318-7	P0DTC2-1262	?TNTSN?	STNTSNKSDTNM	TPGTNTSNQVAVLYQ
Q8WXI7-1	P0DTC1-176	STVLS	STVLSGVPTGAT	AGNATEVPANSTVLS
P34903	P0DTC9-3	APSAS?	TISKGAAPSASS	APSASAFFGM
P34903	P0DTC9-4	APSAS?	TISKGAAPSASS	AQFAPSASA
P34903	P0DTC9-5	APSAS?	TISKGAAPSASS	AQFAPSASAFFGMSR
P34903	P0DTC9-38	APSAS?	TISKGAAPSASS	FAPSASAFF
P34903	P0DTC9-73	APSAS?	TISKGAAPSASS	WPQIAQFAPSASAFF
P34903	P0DTC9-103	APSAS?	TISKGAAPSASS	QFAPSASAFFGMSRI
P34903	P0DTC9-106	APSAS	TISKGAAPSASS	YKHWPQIAQFAPSAS
P34903	P0DTC9-107	APSAS?	TISKGAAPSASS	AQFAPSASAF

P34903	P0DTC9-178	APSAS?	TISKGAAPSASS	QFAPSASAFF
P34903	P0DTC9-440	APSAS?	TISKGAAPSASS	APSASAFFGMSRIGM
P34903	P0DTC9-461	APSAS?	TISKGAAPSASS	IAQFAPSASAFFGMS
P01042-6	P0DTC1-102	AVDAA	DKDLFKAVDAALKKYNS	LSFCAFAVDAAKAY
P01042-7	P0DTC1-102	AVDAA	DLFKAVDAALKK	LSFCAFAVDAAKAY
A0A0C4DGB6-21	P0DTC1-34	TFLKK	DNEETFLKKYLYEIARRHPY	ITFLKKDAPYIVGDV
A0A0C4DGB6-21	P0DTC1-214	D???TFLKK	DNEETFLKKYLYEIARRHPY	ATLVSDIDITFLKKD
A0A0C4DGB6-21	P0DTC1-535	TFLKK	DNEETFLKKYLYEIARRHPY	IDITFLKKDAPYIVG
A0A0C4DGB6-21	P0DTC1-791	D???TFLKK	DNEETFLKKYLYEIARRHPY	LVSDIDITFLKKDAP
A0A0C4DGB6-21	P0DTC1-988	D???TFLKK	DNEETFLKKYLYEIARRHPY	SDIDITFLKKDAPYI
P05783-24	P0DTC9-332	G???AS??SSSRS	GGPGTASRPSSSRSYVTTSTRTY	LPQGTTLPKGFYAEGSRGGSQASSRSSSRSRNSSRNSTP
P05783-24	P0DTC9-441	AS??SSSRS	GGPGTASRPSSSRSYVTTSTRTY	ASSRSSSRSRNSSRN
P05783-24	P0DTC9-454	G???AS??SSSRS	GGPGTASRPSSSRSYVTTSTRTY	GGSQASSRSSSRSRN
P05783-24	P0DTC9-501	G???AS??SSSRS	GGPGTASRPSSSRSYVTTSTRTY	SRGGSQASSRSSSRS
P05783-24	P0DTC9-502	SSSRS	GGPGTASRPSSSRSYVTTSTRTY	SRSSSRSRNSSRNST
P01042-8	P0DTC1-102	AVDAA	KDLFKAVDAALKKYN	LSFCAFAVDAAKAY
H7C2W9-8	P0DTC2-137	VTYVP	KLYTLVTYVPVTTF	HVTYVPAQEKNF
H7C2W9-8	P0DTC2-212	????VTYVP	KLYTLVTYVPVTTF	APHGVVFLHVTYVPAQEKNF
H7C2W9-8	P0DTC2-306	????VTYVP	KLYTLVTYVPVTTF	APHGVVFLHVTYVPA
H7C2W9-8	P0DTC2-422	VTYVP	KLYTLVTYVPVTTF	VTYVPAQEK
H7C2W9-8	P0DTC2-565	VTYVP	KLYTLVTYVPVTTF	VTYVPAQEKNFTTAP
H7C2W9-8	P0DTC2-615	VTYVP	KLYTLVTYVPVTTF	HVTYVPAQEK
P69905-29	P0DTC4-1	LVTLA	LVTLAAHLPAEFTPAVHASLDK F	FLLVTLAIL
P69905-29	P0DTC4-2	LVTLA	LVTLAAHLPAEFTPAVHASLDK F	FLLVTLAILTALRLC
P01042-9	P0DTC1-102	AVDAA	NDKDLFKAVDAALKKYNSQ	LSFCAFAVDAAKAY
P30153	P0DTD1-1185	RFNVA	NVRFNVAKSLQKIGPI	TAHSCNVNRFNVAIT
P30153	P0DTC1-46	?NVAKS	NVRFNVAKSLQKIGPI	VLKKLKKSLNVAKSE
P30153	P0DTC1-89	?NVAKS	NVRFNVAKSLQKIGPI	KLKKSLNVAKSEFDRD
P30153	P0DTC1-656	?NVAKS	NVRFNVAKSLQKIGPI	KSLNVAKSEFDRDAA
P30153	P0DTC1-714	?NVAKS	NVRFNVAKSLQKIGPI	LKKSLNVAKSEFDRD
P47914-2	P0DTD1-819	KPRSQ	RKWHRNGIKKPRSQRY	EFKPRSQMEIDFLEL

P47914-2	P0DTD1-969	KPRSQ	RKWHRNGIKKPRSQRY	KPRSQMEIDFLELAM
P47914-2	P0DTD1-1023	KPRSQ	RKWHRNGIKKPRSQRY	LQEFKPRSQMEIDFL
P47914-2	P0DTD1-1127	KPRSQ	RKWHRNGIKKPRSQRY	QSRNLQEFKPRSQME
P47914-2	P0DTD1-1144	KPRSQ	RKWHRNGIKKPRSQRY	RNLQEFKPRSQMEID
K7ERT8-4	P0DTC8-3	RKSAP	RLRRQPKYPRKSAPR	RVGARKSAPL
K7ERT8-4	P0DTC8-9	RKSAP	RLRRQPKYPRKSAPR	SKWYIRVGARKSAPL
K7ERT8-4	P0DTC8-11	RKSAP	RLRRQPKYPRKSAPR	YIRVGARKSAPLIEL
K7ERT8-4	P0DTC8-12	RKSAP	RLRRQPKYPRKSAPR	ARKSAPLIELCVDEA
K7ERT8-4	P0DTC8-68	RKSAP	RLRRQPKYPRKSAPR	GARKSAPLIELCVDE
K7ERT8-4	P0DTC8-82	RKSAP	RLRRQPKYPRKSAPR	RVGARKSAPLIELCV
C9JXB8-4	P0DTD1-330	SFSGY	SFSGYKIYPGHGRRYARTDGKVF	IEVNSFSGY
C9JXB8-4	P0DTD1-504	SFSGY	SFSGYKIYPGHGRRYARTDGKVF	NSFSGYLKL
C9JXB8-4	P0DTD1-518	SFSGY	SFSGYKIYPGHGRRYARTDGKVF	TIEVNSFSGY
C9JXB8-4	P0DTD1-710	SFSGY	SFSGYKIYPGHGRRYARTDGKVF	VQTIEVNSFSGY
C9JXB8-4	P0DTC1-41	SFSGY	SFSGYKIYPGHGRRYARTDGKVF	NSFSGYLKLTDNVYI
C9JXB8-4	P0DTC1-391	SFSGY	SFSGYKIYPGHGRRYARTDGKVF	EVNSFSGYLKLTDNV
C9JXB8-4	P0DTC1-920	SFSGY	SFSGYKIYPGHGRRYARTDGKVF	PVVQTIEVNSFSGYL
C9JXB8-4	P0DTC1-1217	SFSGY	SFSGYKIYPGHGRRYARTDGKVF	VQTIEVNSFSGYLKL
C9JXB8-5	P0DTD1-330	SFSGY	SFSGYKIYPGHGRRYARTDGKVFQF	IEVNSFSGY
C9JXB8-5	P0DTD1-504	SFSGY	SFSGYKIYPGHGRRYARTDGKVFQF	NSFSGYLKL
C9JXB8-5	P0DTD1-518	SFSGY	SFSGYKIYPGHGRRYARTDGKVFQF	TIEVNSFSGY

C9JXB8-5	P0DTD1-710	SFSGY	SFSGYKIYPGHGRRYARTDGKV FQF	VQTIEVNSFSGY
C9JXB8-5	P0DTC1-41	SFSGY	SFSGYKIYPGHGRRYARTDGKV FQF	NSFSGYLKLTDNVYI
C9JXB8-5	P0DTC1-391	SFSGY	SFSGYKIYPGHGRRYARTDGKV FQF	EVNSFSGYLKLTDNV
C9JXB8-5	P0DTC1-920	SFSGY	SFSGYKIYPGHGRRYARTDGKV FQF	PVVQTIEVNSFSGYL
C9JXB8-5	P0DTC1-1217	SFSGY	SFSGYKIYPGHGRRYARTDGKV FQF	VQTIEVNSFSGYLKL
K7ERT8-6	P0DTC8-3	RKSAP	TLRLRRQPKYPRKSAPRRNKLD HY	RVGARKSAPL
K7ERT8-6	P0DTC8-9	RKSAP	TLRLRRQPKYPRKSAPRRNKLD HY	SKWYIRVGARKSAPL
K7ERT8-6	P0DTC8-11	RKSAP	TLRLRRQPKYPRKSAPRRNKLD HY	YIRVGARKSAPLIEL
K7ERT8-6	P0DTC8-12	RKSAP	TLRLRRQPKYPRKSAPRRNKLD HY	ARKSAPLIELCVDEA
K7ERT8-6	P0DTC8-68	RKSAP	TLRLRRQPKYPRKSAPRRNKLD HY	GARKSAPLIELCVDE
K7ERT8-6	P0DTC8-82	RKSAP	TLRLRRQPKYPRKSAPRRNKLD HY	RVGARKSAPLIELCV
P08575-11	P0DTC1-1072	???KALRK	VDIFQVVKALRK	TEMLAKALRKVPTDN
P08575-12	P0DTC1-1072	???KALRK	VVDIFQVVKALRK	TEMLAKALRKVPTDN
P37108-3	P0DTC5-36	LLESE	VLLESEQFLTE	LLESELVIGAVILRGHLR
P37108-3	P0DTC5-64	LLESE	VLLESEQFLTE	HGTILTRPLLESELV
P37108-3	P0DTC5-72	LLESE	VLLESEQFLTE	LLESELVIGAVILRG
P37108-3	P0DTC5-76	LLESE	VLLESEQFLTE	LTRPLLESELVIGAV
P37108-3	P0DTC5-97	LLESE	VLLESEQFLTE	GTILTRPLLESELVI
P37108-3	P0DTC5-117	LLESE	VLLESEQFLTE	RPLLESEL
P37108-3	P0DTC5-118	LLESE	VLLESEQFLTE	RPLLESELVIGAVIL
P37108-3	P0DTC5-214	LLESE	VLLESEQFLTE	PLHGTILTRPLLESE
P37108-3	P0DTC5-216	LLESE	VLLESEQFLTE	TILTRPLLESELVIG

P05783-26	P0DTC1-80	LGSAL	SLGSALRPSTSRSLYA	GMNGRTILGSALLED
P05783-26	P0DTC1-560	LGSAL	SLGSALRPSTSRSLYA	ILGSALLEDEFTPFD
P05783-26	P0DTC1-839	LGSAL	SLGSALRPSTSRSLYA	NGRTILGSALLEDEF
P05783-26	P0DTC1-973	LGSAL	SLGSALRPSTSRSLYA	RTILGSALLEDEFTP
Q13630-17	P0DTC8-3	?VGARK	NVLHSAFEVGARKVV	RVGARKSAPL
Q13630-17	P0DTC8-7	??VGARK	NVLHSAFEVGARKVV	FYSKWYIRVGARKSA
Q13630-17	P0DTC8-9	??VGARK	NVLHSAFEVGARKVV	SKWYIRVGARKSAPL
Q13630-17	P0DTC8-11	??VGARK	NVLHSAFEVGARKVV	YIRVGARKSAPLIEL
Q13630-17	P0DTC8-82	?VGARK	NVLHSAFEVGARKVV	RVGARKSAPLIELCV
P15880-2	P0DTC2-244	SYLTP	DAISKTYSYLTPDLW	LHRSYLTPGDSSSGWTAGAA
P15880-2	P0DTC2-254	SYLTP	DAISKTYSYLTPDLW	NITRFQTLLALHRSYLTPGD
P15880-2	P0DTC2-739	SYLTP	DAISKTYSYLTPDLW	ALHRSYLTPGDSSSG
P15880-2	P0DTC2-840	SYLTP	DAISKTYSYLTPDLW	LLALHRSYLTPGDSSS
P15880-2	P0DTC2-945	SYLTP	DAISKTYSYLTPDLW	QTLLALHRSYLTPGDSS
P15880-2	P0DTC2-1039	SYLTP	DAISKTYSYLTPDLW	HRSYLTPGDSSS
P15880-2	P0DTC2-1059	SYLTP	DAISKTYSYLTPDLW	LALHRSYLTPGD
P15880-2	P0DTC2-1060	SYLTP	DAISKTYSYLTPDLW	LALHRSYLTPGDSSSGWT
P15880-2	P0DTC2-1135	SYLTP	DAISKTYSYLTPDLW	SYLTPGDSSSGW
P15880-2	P0DTC2-1238	SYLTP	DAISKTYSYLTPDLW	QTLLALHRSYLTPGD
P15880-2	P0DTC2-1256	SYLTP	DAISKTYSYLTPDLW	SYLTPGDSSSGWTAG
P23528-8	P0DTD1-419	KEILV	EEGKEILVGDVGQTVDDPYAT	TLKEILVTY
P23528-8	P0DTD1-536	KEILV	EEGKEILVGDVGQTVDDPYAT	GNCDTLKEILVTYNC
P23528-8	P0DTD1-806	KEILV	EEGKEILVGDVGQTVDDPYAT	DTLKEILVTYNCCDD
P23528-8	P0DTD1-820	KEILV	EEGKEILVGDVGQTVDDPYAT	EGNCDTLKEILVTYN
P23528-8	P0DTD1-855	KEILV	EEGKEILVGDVGQTVDDPYAT	FDEGNCDTLKEILVT
P23528-8	P0DTD1-1004	KEILV	EEGKEILVGDVGQTVDDPYAT	LKEILVTYNCCDDDY
P05164-3	P0DTC2-62	V????DQLTP	VSNEIVRFPTDQLTPD	EVPVAIHADQLTPTW
P05164-3	P0DTC2-173	???IVRFP	VSNEIVRFPTDQLTPD	VQPTESIVRFPNITNLCPF
P05164-3	P0DTC2-221	V????DQLTP	VSNEIVRFPTDQLTPD	DVNCTEVPVAIHADQLTPTW
P05164-3	P0DTC2-235	?DQLTP	VSNEIVRFPTDQLTPD	IHADQLTPTWRVYSTGSNVF
P05164-3	P0DTC2-302	?DQLTP	VSNEIVRFPTDQLTPD	ADQLTPTWRVYSTGS
P05164-3	P0DTC2-345	IVRFP	VSNEIVRFPTDQLTPD	IVRFPNITNLCPFGE
P05164-3	P0DTC2-377	V????DQLTP	VSNEIVRFPTDQLTPD	PVAIHADQLTPTWRV

P05164-3	P0DTC2-379	???IVRFP	VSNEIVRFPTDQLTPD	QPTESIVRFPNITNL
P05164-3	P0DTC2-393	???IVRFP	VSNEIVRFPTDQLTPD	SNFRVQPTESIVRFP
P05164-3	P0DTC2-517	???IVRFP	VSNEIVRFPTDQLTPD	NFRVQPTESIVRFPN
P05164-3	P0DTC2-542	?IVRFP	VSNEIVRFPTDQLTPD	SIVRFPNITNLCPFG
P05164-3	P0DTC2-619	?DQLTP	VSNEIVRFPTDQLTPD	IHADQLTPTW
P05164-3	P0DTC2-669	???IVRFP	VSNEIVRFPTDQLTPD	TESIVRFPNITNL
P05164-3	P0DTC2-673	V????DQLTP	VSNEIVRFPTDQLTPD	VAIHADQLTPTW
P05164-3	P0DTC2-722	V????DQLTP	VSNEIVRFPTDQLTPD	EVPVAIHADQLTPTWRVYSTGSNVFQTRA
P05164-3	P0DTC2-765	?DQLTP	VSNEIVRFPTDQLTPD	ADQLTPTWRVYSTGSN
P05164-3	P0DTC2-776	V????DQLTP	VSNEIVRFPTDQLTPD	CTEVPVAIHADQLTPT
P05164-3	P0DTC2-786	DQLTP	VSNEIVRFPTDQLTPD	DQLTPTWRVYSTGSNV
P05164-3	P0DTC2-796	V????DQLTP	VSNEIVRFPTDQLTPD	EVPVAIHADQLTPTWR
P05164-3	P0DTC2-815	?DQLTP	VSNEIVRFPTDQLTPD	HADQLTPTWRVYSTGS
P05164-3	P0DTC2-822	?DQLTP	VSNEIVRFPTDQLTPD	IHADQLTPTWRVYSTG
P05164-3	P0DTC2-823	?DQLTP	VSNEIVRFPTDQLTPD	IHADQLTPTWRVYSTGSNVFQTR
P05164-3	P0DTC2-844	V????DQLTP	VSNEIVRFPTDQLTPD	NCTEVPVAIHADQLTP
P05164-3	P0DTC2-863	V????DQLTP	VSNEIVRFPTDQLTPD	PVAIHADQLTPTWRVY
P05164-3	P0DTC2-865	V????DQLTP	VSNEIVRFPTDQLTPD	QDVNCTEVPVAIHADQLTPTWRVYS
P05164-3	P0DTC2-895	V????DQLTP	VSNEIVRFPTDQLTPD	TEVPVAIHADQLTPTW
P05164-3	P0DTC2-905	V????DQLTP	VSNEIVRFPTDQLTPD	VAIHADQLTPTWRVYS
P05164-3	P0DTC2-916	V????DQLTP	VSNEIVRFPTDQLTPD	VPVAIHADQLTPTWRV
P05164-3	P0DTC2-925	???IVRFP	VSNEIVRFPTDQLTPD	CTLKSFTVEKGIYQTSNFRVQPTESIVRFPNITNLCPFG
P05164-3	P0DTC2-938	V????DQLTP	VSNEIVRFPTDQLTPD	PVAIHADQLTPTWRVYSTGSNVFQTRAGCLIGAEHVNNS
P05164-3	P0DTC2-943	?DQLTP	VSNEIVRFPTDQLTPD	IHADQLTPTWRVYSTGS
P05164-3	P0DTC2-985	?DQLTP	VSNEIVRFPTDQLTPD	ADQLTPTWRVYS
P05164-3	P0DTC2-990	?DQLTP	VSNEIVRFPTDQLTPD	AIHADQLTPTWR
P05164-3	P0DTC2-1037	?DQLTP	VSNEIVRFPTDQLTPD	HADQLTPTWRVY
P05164-3	P0DTC2-1045	?DQLTP	VSNEIVRFPTDQLTPD	IHADQLTPTWRV
P05164-3	P0DTC2-1046	?DQLTP	VSNEIVRFPTDQLTPD	IHADQLTPTWRVYSTGSN
P05164-3	P0DTC2-1098	V????DQLTP	VSNEIVRFPTDQLTPD	PVAIHADQLTPT
P05164-3	P0DTC2-1100	V????DQLTP	VSNEIVRFPTDQLTPD	QDVNCTEVPVAIHADQLTP
P05164-3	P0DTC2-1109	V????DQLTP	VSNEIVRFPTDQLTPD	QVAVLYQDVNCTEVPVAIHADQLTPTW

P05164-3	P0DTC2-1171	???IVRFP	VSNEIVRFPTDQLTPD	YQTSNFRVQPTESIVRFPN
P05164-3	P0DTC2-1250	???IVRFP	VSNEIVRFPTDQLTPD	SNFRVQPTESIVRFPNITNL
P05164-3	P0DTC2-1257	???IVRFP	VSNEIVRFPTDQLTPD	TESIVRFPNITNLCP
P06733-17	P0DTD1-638	ADLYK	PSRYISPDQLADLYKSFIKD	ITEHSWNADLYKLM
P06733-17	P0DTD1-909	ADLYK	PSRYISPDQLADLYKSFIKD	HSWNADLYKLMGHFA
P06733-17	P0DTD1-1287	ADLYK	PSRYISPDQLADLYKSFIKD	WNADLYKLMGHFAWW
P18669-5	P0DTC1-235	???GKAKK	VRKAMEAVAAQGKAKK	CGNFKVTKGKAKKGA
P18669-5	P0DTC1-375	???GKAKK	VRKAMEAVAAQGKAKK	ESCGNFKVTKGKAKK
P01042-10	P0DTC1-102	AVDAA	DKDLFKAVDAALKKY	LSFCAFAVDAAKAY
P01042-11	P0DTC1-102	AVDAA	DLFKAVDAALKKYN	LSFCAFAVDAAKAY
P05783-35	P0DTC9-332	G???AS??SSSRS	FGGPGTASRPSSSRSYVTTSTRTY	LPQGTTLPKGFYAEGSRGGSQASSRSSSRSRNSSRNSTP
P05783-35	P0DTC9-441	AS??SSSRS	FGGPGTASRPSSSRSYVTTSTRTY	ASSRSSSRSRNSSRN
P05783-35	P0DTC9-454	G???AS??SSSRS	FGGPGTASRPSSSRSYVTTSTRTY	GGSQASSRSSSRSRN
P05783-35	P0DTC9-501	G???AS??SSSRS	FGGPGTASRPSSSRSYVTTSTRTY	SRGGSQASSRSSSRS
P05783-35	P0DTC9-502	SSSRS	FGGPGTASRPSSSRSYVTTSTRTY	SRSSSRSRNSSRNST
P01042-12	P0DTC1-102	AVDAA	KDLFKAVDAALKKY	LSFCAFAVDAAKAY
P62851-2	P0DTD1-821	???LLSKG	KIRGSLARAALQELLSKGLIKL	EGQINDMILSLLSKG
P62851-2	P0DTD1-1016	LLSKG	KIRGSLARAALQELLSKGLIKL	LLSKGRLIIRENNRV
P62851-2	P0DTD1-1032	?LLSKG	KIRGSLARAALQELLSKGLIKL	LSLLSKGRLIIRENN
P62851-2	P0DTD1-1052	???LLSKG	KIRGSLARAALQELLSKGLIKL	MILSLLSKGRLIIRE
P62851-4	P0DTD1-821	???LLSKG	LKIRGSLARAALQELLSKGLIKL	EGQINDMILSLLSKG
P62851-4	P0DTD1-1016	LLSKG	LKIRGSLARAALQELLSKGLIKL	LLSKGRLIIRENNRV
P62851-4	P0DTD1-1032	?LLSKG	LKIRGSLARAALQELLSKGLIKL	LSLLSKGRLIIRENN
P62851-4	P0DTD1-1052	???LLSKG	LKIRGSLARAALQELLSKGLIKL	MILSLLSKGRLIIRE
Q9UHN6-1	P0DTD1-1078	???G?YTFEK	LPFGSYTFEKDFSRGL	NSKVQIGEYTFEKGD
Q9UHN6-1	P0DTD1-1115	?G?YTFEK	LPFGSYTFEKDFSRGL	QIGEYTFEKGDYGDA
Q9UHN6-1	P0DTD1-1310	YTFEK	LPFGSYTFEKDFSRGL	YTFEKGDYGDAVVYR
P01042-13	P0DTC1-102	AVDAA	NDKDLFKAVDAALKKY	LSFCAFAVDAAKAY

P01042-14	P0DTC1-102	AVDAA	NDKDLFKAVDAALKKYNS	LSFCAFAVDAAKAY
Q96PV4	P0DTD1-1145	KAVFI	LCAYRVLGRMFRREDNAKAVFI EL	RNPAWRKAVFISPYN
Q96PV4	P0DTD1-1289	KAVFI	LCAYRVLGRMFRREDNAKAVFI EL	WRKAVFISPYNSQNA
P47914-4	P0DTD1-819	KPRSQ	RNGIKKPRSQRY	EFKPRSQMEIDFLEL
P47914-4	P0DTD1-969	KPRSQ	RNGIKKPRSQRY	KPRSQMEIDFLELAM
P47914-4	P0DTD1-1023	KPRSQ	RNGIKKPRSQRY	LQEFKPRSQMEIDFL
P47914-4	P0DTD1-1127	KPRSQ	RNGIKKPRSQRY	QSRNLQEFKPRSQME
P47914-4	P0DTD1-1144	KPRSQ	RNGIKKPRSQRY	RNLQEFKPRSQMEID
P07355-8	P0DTD1-801	RDLYD	AEDGSVIDYELIDQDARDLYDA	DRDLYDKLQFTSLEI
P07355-8	P0DTD1-928	RDLYD	AEDGSVIDYELIDQDARDLYDA	ILCIMSDRDLYDKLQ
Q7Z4T9	P0DTD1-386	VYSFL	AELVYSFLIPEVQKYFVKEKVRN AQ	PVYSFLPGVYSVIYL
Q7Z4T9	P0DTD1-833	?KYFVK	AELVYSFLIPEVQKYFVKEKVRN AQ	ELTSMKYFVKIGPER
Q8N729	P0DTC9-145	LALLL	APASRPRLALLLLLLLLPLP	RMAGNGGDAALALLL
Q8N729	P0DTC9-225	ALLLL	APASRPRLALLLLLLLLPLP	ALLLLDRLNQLESKMS
Q8N729	P0DTC9-228	LALLL	APASRPRLALLLLLLLLPLP	ARMAGNGGDAALALLL
Q8N729	P0DTC9-363	ALLLL	APASRPRLALLLLLLLLPLP	ALLLLDRLNQLESKMSG
Q8N729	P0DTC9-439	ALLLL	APASRPRLALLLLLLLLPLP	ALLLLDRLNQLESKM
Q6H3X3	P0DTC3-4	RATAT	AQPRATAT	FVRATATIPI
Q6H3X3	P0DTC3-81	RATAT	AQPRATAT	KDATPSDFVRATATIP
Q6H3X3	P0DTC3-94	RATAT	AQPRATAT	ATPSDFVRATAT
Q6H3X3	P0DTC3-103	RATAT	AQPRATAT	PSDFVRATATIP
Q6H3X3	P0DTC3-108	RATAT	AQPRATAT	VRATATIPIQAS
Q6H3X3	P0DTC3-114	RATAT	AQPRATAT	ATPSDFVRATATIPI
O95450	P0DTC9-16	ALLLL	ARRLLCPALLLLLLLLP	LALLLLDRL
O95450	P0DTC9-225	ALLLL	ARRLLCPALLLLLLLLP	ALLLLDRLNQLESKMS
O95450	P0DTC9-262	ALLLL	ARRLLCPALLLLLLLLP	LALLLLDRLNQLESKM
O95450	P0DTC9-363	ALLLL	ARRLLCPALLLLLLLLP	ALLLLDRLNQLESKMSG
O95450	P0DTC9-439	ALLLL	ARRLLCPALLLLLLLLP	ALLLLDRLNQLESKM

O75486	P0DTC9-15	?KDKKK	ARVITPEDLLFLMRKDKKKLRRLL	KTFPPTEPKKDKKKK
O75486	P0DTC9-35	?KDKKK	ARVITPEDLLFLMRKDKKKLRRLL	YKTFPPTEPKKDKKK
O75486	P0DTC9-59	?KDKKK	ARVITPEDLLFLMRKDKKKLRRLL	KKDKKKKADETQALPQRQ
O75486	P0DTC9-61	?KDKKK	ARVITPEDLLFLMRKDKKKLRRLL	KTFPPTEPKKDKKKKADE
O75486	P0DTC9-87	?KDKKK	ARVITPEDLLFLMRKDKKKLRRLL	TEPKKDKKKKADETQ
O75486	P0DTC9-109	?KDKKK	ARVITPEDLLFLMRKDKKKLRRLL	DAYKTFPPTEPKKDKKKKADETQA
O75486	P0DTC9-110	?KDKKK	ARVITPEDLLFLMRKDKKKLRRLL	FPPTEPKKDKKKKADETQALPQRQKKQQTVTLLPAADLDDFSKQLQ
O75486	P0DTC9-170	?KDKKK	ARVITPEDLLFLMRKDKKKLRRLL	KTFPPTEPKKDKKK
O75486	P0DTC9-330	?KDKKK	ARVITPEDLLFLMRKDKKKLRRLL	DKDPNFKDQVILLNKHIDAYKTFPPTEPKKDKKKKADET
O75486	P0DTC9-346	?KDKKK	ARVITPEDLLFLMRKDKKKLRRLL	KHIDAYKTFPPTEPKKDKKKK
O75486	P0DTC9-351	?KDKKK	ARVITPEDLLFLMRKDKKKLRRLL	PKKDKKKKTDEAQPLPQRQKKQP
O75486	P0DTC9-366	?KDKKK	ARVITPEDLLFLMRKDKKKLRRLL	AYKTFPPTEPKKDKKKK
O75486	P0DTC9-387	KDKKK	ARVITPEDLLFLMRKDKKKLRRLL	KDKKKKADETQALPQR
O75486	P0DTC9-388	?KDKKK	ARVITPEDLLFLMRKDKKKLRRLL	KKDKKKKADETQ
O75486	P0DTC9-467	KDKKK	ARVITPEDLLFLMRKDKKKLRRLL	KDKKKKADETQALPQ
O75486	P0DTC9-485	?KDKKK	ARVITPEDLLFLMRKDKKKLRRLL	PKKDKKKKADETQAL
O75486	P0DTC9-487	?KDKKK	ARVITPEDLLFLMRKDKKKLRRLL	PPTEPKKDKKKKADE

O75486	P0DTC9-503	?KDKKK	ARVITPEDLLFLMRKDKKKLRRLL	TFPPTEPKKDKKKKA
O75486	P0DTC2-8	EDLLF	ARVITPEDLLFLMRKDKKKLRRLL	EDLLFNKVTLADAGF
O75486	P0DTC2-76	EDLLF	ARVITPEDLLFLMRKDKKKLRRLL	KPSKRSFIEDLLFNK
O75486	P0DTC2-90	EDLLF	ARVITPEDLLFLMRKDKKKLRRLL	PSKPSKRSFIEDLLFNKV
O75486	P0DTC2-247	EDLLF	ARVITPEDLLFLMRKDKKKLRRLL	LPDPSKPSKRSFIEDLLFNK
O75486	P0DTC2-276	EDLLF	ARVITPEDLLFLMRKDKKKLRRLL	SFIEDLLFNKVTLADAGFIK
O75486	P0DTC2-388	EDLLF	ARVITPEDLLFLMRKDKKKLRRLL	SFIEDLLFNKVTLAD
O75486	P0DTC2-448	EDLLF	ARVITPEDLLFLMRKDKKKLRRLL	PDPSKPSKRSFIEDLLFNKVTLAD
O75486	P0DTC2-453	EDLLF	ARVITPEDLLFLMRKDKKKLRRLL	RSFIEDLLFNK
O75486	P0DTC2-454	EDLLF	ARVITPEDLLFLMRKDKKKLRRLL	RSFIEDLLFNKV
O75486	P0DTC2-532	EDLLF	ARVITPEDLLFLMRKDKKKLRRLL	PSKPSKRSFIEDLLF
O75486	P0DTC2-543	EDLLF	ARVITPEDLLFLMRKDKKKLRRLL	SKRSFIEDLLFNKVT
O75486	P0DTC2-707	EDLLF	ARVITPEDLLFLMRKDKKKLRRLL	RSFIEDLLFNKVTLA
O75486	P0DTC2-720	EDLLF	ARVITPEDLLFLMRKDKKKLRRLL	EDLLFN
O75486	P0DTC2-759	EDLLF	ARVITPEDLLFLMRKDKKKLRRLL	SKRSFIEDLLF
O75486	P0DTC2-764	EDLLF	ARVITPEDLLFLMRKDKKKLRRLL	SKRSFIEDLLFNKVTLA
O75486	P0DTC2-785	EDLLF	ARVITPEDLLFLMRKDKKKLRRLL	DPSKPSKRSFIEDLLF

O75486	P0DTC2-799	EDLLF	ARVITPEDLLFLMRKDKKKLRRLL	FIEDLLFNKVTLADAG
O75486	P0DTC2-835	EDLLF	ARVITPEDLLFLMRKDKKKLRRLL	KPSKRSFIEDLLFNKV
O75486	P0DTC2-837	EDLLF	ARVITPEDLLFLMRKDKKKLRRLL	KRSFIEDLLFNKVTLA
O75486	P0DTC2-852	EDLLF	ARVITPEDLLFLMRKDKKKLRRLL	PDPSKPSKRSFIEDLLFNKVTLADAG
O75486	P0DTC2-860	EDLLF	ARVITPEDLLFLMRKDKKKLRRLL	PSKPSKRSFIEDLLFN
O75486	P0DTC2-861	EDLLF	ARVITPEDLLFLMRKDKKKLRRLL	PSKRSFIEDLLFNKVT
O75486	P0DTC2-875	EDLLF	ARVITPEDLLFLMRKDKKKLRRLL	RSFIEDLLFNKVTLAD
O75486	P0DTC2-880	EDLLF	ARVITPEDLLFLMRKDKKKLRRLL	SFIEDLLFNKVTLADA
O75486	P0DTC2-883	EDLLF	ARVITPEDLLFLMRKDKKKLRRLL	SKPSKRSFIEDLLFNK
O75486	P0DTC2-884	EDLLF	ARVITPEDLLFLMRKDKKKLRRLL	SKRSFIEDLLFNKVTL
O75486	P0DTC2-934	EDLLF	ARVITPEDLLFLMRKDKKKLRRLL	NFSQILPDPSKPSKRSFIEDLLFNKVTLADAGFIKQYGD
O75486	P0DTC2-983	EDLLF	ARVITPEDLLFLMRKDKKKLRRLL	SKPSKRSFIEDLLF
O75486	P0DTC2-1053	EDLLF	ARVITPEDLLFLMRKDKKKLRRLL	KRSFIEDLLFNKVT
O75486	P0DTC2-1124	EDLLF	ARVITPEDLLFLMRKDKKKLRRLL	SKRSFIEDLLFNKVTLADA
O75486	P0DTC1-517	?V?????LLFLM	ARVITPEDLLFLMRKDKKKLRRLL	HVVAFNTLLFLMSFT
O75486	P0DTC1-1151	LLFLM	ARVITPEDLLFLMRKDKKKLRRLL	VAFNTLLFLMSFTVL
Q8MH48	P0DTC1-607	KDYLA	AYDGKDYLALNEDLRSWTA	KAYKDYLASGGQPIT
Q8MH48	P0DTC1-1267	KDYLA	AYDGKDYLALNEDLRSWTA	YKDYLASGGQPITNC

Q8NAP8	P0DTD1-220	VTDTP	CDSCTCVTDTPDDDDDLM	VTDTPKGPK
Q8NAP8	P0DTC1-15	C??VTDTP	CDSCTCVTDTPDDDDDLM	PCRFVTDTPKGPKVK
Q8NAP8	P0DTC1-116	C??VTDTP	CDSCTCVTDTPDDDDDLM	PPCRFVTDTPKGPK
Q8NAP8	P0DTC1-356	C??VTDTP	CDSCTCVTDTPDDDDDLM	ELEPPCRFVTDTPKG
Q8N446	P0DTC2-52	C???CCSCG	CPFCCCSCGD	CLKGCCSCGSCCKFD
Q8N446	P0DTC2-216	C???CCSCG	CPFCCCSCGD	CMTSCCSCLKGCCSCGSCCK
Q8N446	P0DTC2-230	CCSCG	CPFCCCSCGD	GCCSCGSCCKFDEDDSEPVL
Q8N446	P0DTC2-433	CCSCG	CPFCCCSCGD	CCSCGSCCKFDEDDSEPVLKGVKL
Q8N446	P0DTC2-498	CCSCG	CPFCCCSCGD	KGCCSCGSCCKFDED
Q8N446	P0DTC2-771	CCSCG	CPFCCCSCGD	CCSCGSCCKFDEDDSE
Q8N446	P0DTC2-772	CCSCG	CPFCCCSCGD	CCSCGSCCKFDEDDSEPVLKGVKLHYT
Q8N446	P0DTC2-924	C???CCSCG	CPFCCCSCGD	CCMTSCCSCLKGCCSCGSCCKFDEDDSEPVLKGVKLHYT
Q8N446	P0DTC2-1179	CCSCG	CPFCCCSCGD	CCSCGSCCKFDEDDS
Q9HCE3	P0DTC1-163	KLKAL	DYDKLKALGGEN	ADSIIGGAKLKALN
P07355-9	P0DTD1-801	RDLYD	EDGSVIDYELIDQDARDLYDA	DRDLYDKLQFTSLEI
P07355-9	P0DTD1-928	RDLYD	EDGSVIDYELIDQDARDLYDA	ILCIMSDRDLYDKLQ
Q92688-1	P0DTD1-132	D?EEEEF?	EEDEDDEDGEEEEFDEEDDEDEDVE	GDCEEEEFEPSTQYEY
Q92688-1	P0DTD1-612	EEEEF?	EEDEDDEDGEEEEFDEEDDEDEDVE	EEEEFEPSTQYE
Q92688-1	P0DTC1-234	EEEEF?	EEDEDDEDGEEEEFDEEDDEDEDVE	CEEEEFEPSTQYEYG
Q92688-1	P0DTC1-261	?EDE???D?EEEEF?	EEDEDDEDGEEEEFDEEDDEDEDVE	DEDEEEGDCEEEEFE
Q92688-1	P0DTC1-262	DE???D?EEEEF?	EEDEDDEDGEEEEFDEEDDEDEDVE	DEEEGDCEEEEFEPS
Q92688-1	P0DTC1-327	???D?EEEEF?	EEDEDDEDGEEEEFDEEDDEDEDVE	EEGDCEEEEFEPSTQ
Q92688-1	P0DTC1-453	D?EEEEF?	EEDEDDEDGEEEEFDEEDDEDEDVE	GDCEEEEFEPSTQYE
Q9NW61	P0DTC2-76	SFIED	EEPGTFSISFIEDPERKYH	KPSKRSFIEDLLFNK
Q9NW61	P0DTC2-90	SFIED	EEPGTFSISFIEDPERKYH	PSKPSKRSFIEDLLFNKV

Q9NW61	P0DTC2-247	SFIED	EEPGTFSISFIEDPERKYH	LPDPSKPSKRSFIEDLLFNK
Q9NW61	P0DTC2-276	SFIED	EEPGTFSISFIEDPERKYH	SFIEDLLFNKVTLADAGFIK
Q9NW61	P0DTC2-357	SFIED	EEPGTFSISFIEDPERKYH	LPDPSKPSKRSFIED
Q9NW61	P0DTC2-388	SFIED	EEPGTFSISFIEDPERKYH	SFIEDLLFNKVTLAD
Q9NW61	P0DTC2-448	SFIED	EEPGTFSISFIEDPERKYH	PDPSKPSKRSFIEDLLFNKVTLAD
Q9NW61	P0DTC2-453	SFIED	EEPGTFSISFIEDPERKYH	RSFIEDLLFNK
Q9NW61	P0DTC2-454	SFIED	EEPGTFSISFIEDPERKYH	RSFIEDLLFNKV
Q9NW61	P0DTC2-532	SFIED	EEPGTFSISFIEDPERKYH	PSKPSKRSFIEDLLF
Q9NW61	P0DTC2-543	SFIED	EEPGTFSISFIEDPERKYH	SKRSFIEDLLFNKVT
Q9NW61	P0DTC2-707	SFIED	EEPGTFSISFIEDPERKYH	RSFIEDLLFNKVTLA
Q9NW61	P0DTC2-759	SFIED	EEPGTFSISFIEDPERKYH	SKRSFIEDLLF
Q9NW61	P0DTC2-764	SFIED	EEPGTFSISFIEDPERKYH	SKRSFIEDLLFNKVTLA
Q9NW61	P0DTC2-785	SFIED	EEPGTFSISFIEDPERKYH	DPSKPSKRSFIEDLLF
Q9NW61	P0DTC2-826	SFIED	EEPGTFSISFIEDPERKYH	ILPDPSKPSKRSFIED
Q9NW61	P0DTC2-835	SFIED	EEPGTFSISFIEDPERKYH	KPSKRSFIEDLLFNKV
Q9NW61	P0DTC2-837	SFIED	EEPGTFSISFIEDPERKYH	KRSFIEDLLFNKVTLA
Q9NW61	P0DTC2-851	SFIED	EEPGTFSISFIEDPERKYH	PDPSKPSKRSFIEDLL
Q9NW61	P0DTC2-852	SFIED	EEPGTFSISFIEDPERKYH	PDPSKPSKRSFIEDLLFNKVTLADAG
Q9NW61	P0DTC2-860	SFIED	EEPGTFSISFIEDPERKYH	PSKPSKRSFIEDLLFN
Q9NW61	P0DTC2-861	SFIED	EEPGTFSISFIEDPERKYH	PSKRSFIEDLLFNKVT
Q9NW61	P0DTC2-867	SFIED	EEPGTFSISFIEDPERKYH	QILPDPSKPSKRSFIED
Q9NW61	P0DTC2-875	SFIED	EEPGTFSISFIEDPERKYH	RSFIEDLLFNKVTLAD
Q9NW61	P0DTC2-880	SFIED	EEPGTFSISFIEDPERKYH	SFIEDLLFNKVTLADA
Q9NW61	P0DTC2-883	SFIED	EEPGTFSISFIEDPERKYH	SKPSKRSFIEDLLFNK
Q9NW61	P0DTC2-884	SFIED	EEPGTFSISFIEDPERKYH	SKRSFIEDLLFNKVTL
Q9NW61	P0DTC2-934	SFIED	EEPGTFSISFIEDPERKYH	NFSQILPDPSKPSKRSFIEDLLFNKVTLADAGFIKQYGD
Q9NW61	P0DTC2-983	SFIED	EEPGTFSISFIEDPERKYH	SKPSKRSFIEDLLF
Q9NW61	P0DTC2-1053	SFIED	EEPGTFSISFIEDPERKYH	KRSFIEDLLFNKVT
Q9NW61	P0DTC2-1124	SFIED	EEPGTFSISFIEDPERKYH	SKRSFIEDLLFNKVTLADA
Q9NW61	P0DTC2-1232	SFIED	EEPGTFSISFIEDPERKYH	PDPSKPSKRSFIEDL
P0C7M7	P0DTD1-440	VSSPD??	ESAVVSSPDQIRGEVVK	VSSPDAVTAY
P0C7M7	P0DTC1-98	VSSPD	ESAVVSSPDQIRGEVVK	LKVPATVSVSSPDA
P0C7M7	P0DTC1-888	VSSPD??	ESAVVSSPDQIRGEVVK	PATVSVSSPDAVTAY

P0C7M7	P0DTC1-1043	VSSPD??	ESAVVSSPDQIRGEVVK	SVSSPDAVTAYNGYL
P32246	P0DTD1-941	RARTV	FALRARTVTFG	ISAKNRARTVAGVSI
Q96GD3	P0DTC9-16	ALLLL	GKALLLLRSDMMMKYM	LALLLLDRL
Q96GD3	P0DTC9-42	ALLLL	GKALLLLRSDMMMKYM	GDAALALLLLDRLNQL
Q96GD3	P0DTC9-93	ALLLL	GKALLLLRSDMMMKYM	DAALALLLLDRLNQL
Q96GD3	P0DTC9-118	ALLLL	GKALLLLRSDMMMKYM	PARMAGNGGDAALALLLLDRLNQLESKMSGKGQQQQGQ
Q96GD3	P0DTC9-120	ALLLL	GKALLLLRSDMMMKYM	AALALLLLDRLNQLE
Q96GD3	P0DTC9-157	ALLLL	GKALLLLRSDMMMKYM	AGNGGDAALALLLLD
Q96GD3	P0DTC9-216	ALLLL	GKALLLLRSDMMMKYM	AALALLLLDRLNQLES
Q96GD3	P0DTC9-221	ALLLL	GKALLLLRSDMMMKYM	AGNGGDAALALLLLDR
Q96GD3	P0DTC9-224	ALLLL	GKALLLLRSDMMMKYM	ALALLLLDRLNQLESK
Q96GD3	P0DTC9-225	ALLLL	GKALLLLRSDMMMKYM	ALLLLDRLNQLESKMS
Q96GD3	P0DTC9-229	ALLLL	GKALLLLRSDMMMKYM	ARMAGNGGDAALALLLLDRLNQLESKMSGKG
Q96GD3	P0DTC9-231	ALLLL	GKALLLLRSDMMMKYM	DAALALLLLDRLNQLE
Q96GD3	P0DTC9-245	ALLLL	GKALLLLRSDMMMKYM	GGDAALALLLLDRLNQ
Q96GD3	P0DTC9-249	ALLLL	GKALLLLRSDMMMKYM	GNGGDAALALLLLDRL
Q96GD3	P0DTC9-262	ALLLL	GKALLLLRSDMMMKYM	LALLLLDRLNQLESKM
Q96GD3	P0DTC9-277	ALLLL	GKALLLLRSDMMMKYM	MAGNGGDAALALLLLD
Q96GD3	P0DTC9-282	ALLLL	GKALLLLRSDMMMKYM	NGGDAALALLLLDRLN
Q96GD3	P0DTC9-313	ALLLL	GKALLLLRSDMMMKYM	RMAGNGGDAALALLLL
Q96GD3	P0DTC9-339	ALLLL	GKALLLLRSDMMMKYM	SSRGTSPARMAGNGGDAALALLLLDRLNQLESKMSGKGQ
Q96GD3	P0DTC9-359	ALLLL	GKALLLLRSDMMMKYM	AALALLLLDRLNQLESKMS
Q96GD3	P0DTC9-363	ALLLL	GKALLLLRSDMMMKYM	ALLLLDRLNQLESKMSG
Q96GD3	P0DTC9-394	ALLLL	GKALLLLRSDMMMKYM	MAGNGGDAALALLLLDR
Q96GD3	P0DTC9-417	ALLLL	GKALLLLRSDMMMKYM	RMAGNGGDAALALLLLDRL
Q96GD3	P0DTC9-438	ALLLL	GKALLLLRSDMMMKYM	ALALLLLDRLNQLES
Q96GD3	P0DTC9-439	ALLLL	GKALLLLRSDMMMKYM	ALLLLDRLNQLESKM
Q96GD3	P0DTC9-453	ALLLL	GKALLLLRSDMMMKYM	GGDAALALLLLDRLN
Q96GD3	P0DTC9-456	ALLLL	GKALLLLRSDMMMKYM	GNGGDAALALLLLDR
Q96GD3	P0DTC9-480	ALLLL	GKALLLLRSDMMMKYM	MAGNGGDAALALLLL

P82914	P0DTC9-55	YYRRA?RR	GLGIEYTFPPLYYRRAHRRFVTKK	YYRRATRRIR
P82914	P0DTC9-65	YYRRA?RR	GLGIEYTFPPLYYRRAHRRFVTKK	DDQIGYYRRATRRIR
P82914	P0DTC9-74	YYRRA?RR	GLGIEYTFPPLYYRRAHRRFVTKK	DQIGYYRRATRRIR
P82914	P0DTC9-97	YYRRA?RR	GLGIEYTFPPLYYRRAHRRFVTKK	IGYYRRATRRIRGGD
P82914	P0DTC9-137	YYRRA	GLGIEYTFPPLYYRRAHRRFVTKK	NSSPDDQIGYYRRAT
P82914	P0DTC9-188	YYRRA?RR	GLGIEYTFPPLYYRRAHRRFVTKK	YYRRATRRIRGGDGK
P82914	P0DTC9-368	YYRRA?RR	GLGIEYTFPPLYYRRAHRRFVTKK	DDQIGYYRRATRRIRGGDG
P82914	P0DTC9-424	YYRRA	GLGIEYTFPPLYYRRAHRRFVTKK	SPDDQIGYYRRA
P82914	P0DTC9-484	YYRRA?RR	GLGIEYTFPPLYYRRAHRRFVTKK	PDDQIGYYRRATRRI
P07355-12	P0DTD1-801	RDLYD	GSVIDYELIDQDARDLYDAGVKRKG	DRDLYDKLQFTSLEI
P07355-12	P0DTD1-928	RDLYD	GSVIDYELIDQDARDLYDAGVKRKG	ILCIMSDRDLYDKLQ
Q6V0I7	P0DTC2-64	?LTGTG??	ITATDSGSPALTGTGTINV	FNFNGLTGTGVLTES
Q6V0I7	P0DTC2-251	?LTGTG??	ITATDSGSPALTGTGTINV	NGLTGTGVLTESNKKFLPFQ
Q6V0I7	P0DTC2-291	?LTGTG??	ITATDSGSPALTGTGTINV	VKNKCVNFNFNGLTGTGVLT
Q6V0I7	P0DTC2-364	LTGTG??	ITATDSGSPALTGTGTINV	LTGTGVLTESNKKFL
Q6V0I7	P0DTC2-482	?LTGTG??	ITATDSGSPALTGTGTINV	GLTGTGVLTESNKKF
Q6V0I7	P0DTC2-803	?LTGTG??	ITATDSGSPALTGTGTINV	FNFNGLTGTGVLTESN
Q6V0I7	P0DTC2-828	?LTGTG??	ITATDSGSPALTGTGTINV	KCVNFNFNGLTGTGVL
Q6V0I7	P0DTC2-847	?LTGTG	ITATDSGSPALTGTGTINV	NKCVNFNFNGLTGTGV
Q6V0I7	P0DTC2-848	?LTGTG??	ITATDSGSPALTGTGTINV	NKCVNFNFNGLTGTGVL
Q6V0I7	P0DTC2-929	?LTGTG??	ITATDSGSPALTGTGTINV	FNFNGLTGTGVLTESNKKFLPFQQFGRDIADTTDAVRDP

Q6V0I7	P0DTC2-936	?LTGTG??	ITATDSGSPALTGTGTINV	PATVCGPKKSTNLVKNKCVNFNFNGLTGTGVLTESNKKF
Q6V0I7	P0DTC2-966	?LTGTG??	ITATDSGSPALTGTGTINV	GLTGTGVLTESNKKFLPFQQF
Q6V0I7	P0DTC1-204	P???GTGTI	ITATDSGSPALTGTGTINV	ARFPKSDGTGTIYTE
Q6V0I7	P0DTC1-271	GTGTI	ITATDSGSPALTGTGTINV	DGTGTIYTELEPPCR
Q6V0I7	P0DTC1-435	P???GTGTI	ITATDSGSPALTGTGTINV	FPKSDGTGTIYTELE
Q6V0I7	P0DTC1-654	GTGTI	ITATDSGSPALTGTGTINV	KSDGTGTIYTELEPP
P11487	P0DTD1-408	??V??GIVAI	ITAVEVGIVAIRGLFSGRYLAMNK	SIVAGGIVAIVVTCL
P11487	P0DTC1-277	??V??GIVAI	ITAVEVGIVAIRGLFSGRYLAMNK	DISASIVAGGIVAIV
P11487	P0DTC1-465	GIVAI	ITAVEVGIVAIRGLFSGRYLAMNK	GGIVAIVVTCLAYYF
Q9Y6P5	P0DTD1-398	SEDAQ	KEILQVGSEDAQMH	SEDAQGMDNL
Q9Y6P5	P0DTC1-114	SEDAQ	KEILQVGSEDAQMH	NSFDVLKSEDAQGMDNL
Q9Y6P5	P0DTC1-304	SEDAQ	KEILQVGSEDAQMH	DVLKSEDAQGMDNLA
Q9Y6P5	P0DTC1-385	SEDAQ	KEILQVGSEDAQMH	ETSNSFDVLKSEDAQ
Q9Y6P5	P0DTC1-717	SEDAQ	KEILQVGSEDAQMH	LKSEDAQGMDNLACE
Q9Y6P5	P0DTC1-994	SEDAQ	KEILQVGSEDAQMH	SEDAQGMDNLACEDL
Q9Y6P5	P0DTC1-1004	SEDAQ	KEILQVGSEDAQMH	SFDVLKSEDAQGMDN
Q9NWB7	P0DTC2-628	KSNLK	KLKLLRYEEEFLRKSNLKAPSRHYF	KSNLKPFER
Q6UX71-2	P0DTC9-34	VTQAF	KPGDQILDWQYGVTQAFPHTEE	VTQAFGRRGPEQTQG
Q6UX71-2	P0DTC9-46	Y?VTQAF	KPGDQILDWQYGVTQAFPHTEE	KAYNVTQAF
Q6UX71-2	P0DTC9-67	Y?VTQAF	KPGDQILDWQYGVTQAFPHTEE	KAYNVTQAFGRRGPE
Q6UX71-2	P0DTC9-131	Y?VTQAF	KPGDQILDWQYGVTQAFPHTEE	KRTATKAYNVTQAFG
Q6UX71-2	P0DTC9-201	Y?VTQAF	KPGDQILDWQYGVTQAFPHTEE	TVTKKSAAEASKKPRQKRTATKAYNVTQAF

Q6UX71-2	P0DTC9-329	Y?VTQAF	KPGDQILDWQYGVTQAFPHTE E	ATKAYNVTQAFGRRG
Q6UX71-2	P0DTC9-336	Y?VTQAF	KPGDQILDWQYGVTQAFPHTE E	QQGQTVTKKSAAEASKKPRQKRTATKAYNVTQAFGRR GP
Q6UX71-2	P0DTC9-389	Y?VTQAF	KPGDQILDWQYGVTQAFPHTE E	KPRQKRTATKAYNVTQAFGRRGPEQTQ
Q6UX71-2	P0DTC9-510	Y?VTQAF	KPGDQILDWQYGVTQAFPHTE E	YNVTQAFGRRGPEQT
Q9NTJ3	P0DTC2-170	VEAEV?	KVEAEVKRLH	VEAEVQIDRLITGR
Q9NTJ3	P0DTC2-413	KVEAE	KVEAEVKRLH	VLNDILSRLDKVEAE
Q8WXH0	P0DTD1-536	DTLKE	LENHVNDIKKPFVIKERDTLKER ER	GNCDTLKEILVTYNC
Q8WXH0	P0DTD1-741	DTLKE	LENHVNDIKKPFVIKERDTLKER ER	ALRHFDEGNCDTLKE
Q8WXH0	P0DTD1-806	DTLKE	LENHVNDIKKPFVIKERDTLKER ER	DTLKEILVTYNCCDD
Q8WXH0	P0DTD1-820	DTLKE	LENHVNDIKKPFVIKERDTLKER ER	EGNCDTLKEILVTYN
Q8WXH0	P0DTD1-855	DTLKE	LENHVNDIKKPFVIKERDTLKER ER	FDEGNCDTLKEILVT
Q8WXH0	P0DTD1-1134	DTLKE	LENHVNDIKKPFVIKERDTLKER ER	RHFDEGNCDTLKEIL
Q9UHN6-2	P0DTD1-1078	???G?YTFEK	LPFGSYTFEKDFSRG	NSKVQIGEYTFEKGD
Q9UHN6-2	P0DTD1-1115	?G?YTFEK	LPFGSYTFEKDFSRG	QIGEYTFEKGDYGDA
Q9UHN6-2	P0DTD1-1310	YTFEK	LPFGSYTFEKDFSRG	YTFEKGDYGDAVVYR
A0A0C4DGB6-27	P0DTD1-22	LPSLA	LPSLAADFVESKD	FLLPSLATV
A0A0C4DGB6-27	P0DTD1-131	LPSLA	LPSLAADFVESKD	FLCLFLLPSLATV
A0A0C4DGB6-27	P0DTD1-493	LPSLA	LPSLAADFVESKD	LFLLPSLATV
A0A0C4DGB6-27	P0DTC1-10	LPSLA	LPSLAADFVESKD	LCLFLLPSLATVAYF
A0A0C4DGB6-27	P0DTC1-728	LPSLA	LPSLAADFVESKD	LLPSLATVAYFNMVY
P48039	P0DTC1-354	VLQVR	LVLQVRQRVKPDRKPKLKP	EKTHVQLSLPVLQVR
P48039	P0DTC1-770	VLQVR	LVLQVRQRVKPDRKPKLKP	LSLPVLQVRDVLVRG
P48039	P0DTC1-1091	VLQVR	LVLQVRQRVKPDRKPKLKP	THVQLSLPVLQVRDV

P48039	P0DTC1-1201	VLQVR	LVLQVRQRVKPDRKPKLKP	VLQVRDVLVRGFGDS
P48039	P0DTC1-1213	VLQVR	LVLQVRQRVKPDRKPKLKP	VQLSLPVLQVRDVLV
A0A0C4DGB6-28	P0DTD1-22	LPSLA	PADLPSLAADFVESKD	FLLPSLATV
A0A0C4DGB6-28	P0DTD1-131	LPSLA	PADLPSLAADFVESKD	FLCLFLLPSLATV
A0A0C4DGB6-28	P0DTD1-493	LPSLA	PADLPSLAADFVESKD	LFLLPSLATV
A0A0C4DGB6-28	P0DTC1-10	LPSLA	PADLPSLAADFVESKD	LCLFLLPSLATVAYF
A0A0C4DGB6-28	P0DTC1-728	LPSLA	PADLPSLAADFVESKD	LLPSLATVAYFNMVY
Q13507	P0DTC2-23	?VVLSF	PQIISEGLYAIAVVLSFSRIAYILP	QPYRVVVLSF
Q13507	P0DTC2-117	VVLSF	PQIISEGLYAIAVVLSFSRIAYILP	VVLSFELLHAPATVC
Q13507	P0DTC2-136	?VVLSF	PQIISEGLYAIAVVLSFSRIAYILP	GYQPYRVVVLSF
Q13507	P0DTC2-233	?VVLSF	PQIISEGLYAIAVVLSFSRIAYILP	GYQPYRVVVLSFELLHAPAT
Q13507	P0DTC2-368	?VVLSF	PQIISEGLYAIAVVLSFSRIAYILP	NGVGYQPYRVVVLSF
Q13507	P0DTC2-380	?VVLSF	PQIISEGLYAIAVVLSFSRIAYILP	QPYRVVVLSFELLHA
Q13507	P0DTC2-540	?VVLSF	PQIISEGLYAIAVVLSFSRIAYILP	RVVVLSFELLHAPAT
Q13507	P0DTC2-574	?VVLSF	PQIISEGLYAIAVVLSFSRIAYILP	YQPYRVVVLSFELLH
Q13507	P0DTC2-971	?VVLSF	PQIISEGLYAIAVVLSFSRIAYILP	NGVGYQPHRVVVLSF
Q13507	P0DTC2-1117	?VVLSF	PQIISEGLYAIAVVLSFSRIAYILP	RVVVLSFELLHAPATVCGP
Q13507	P0DTC2-1202	?VVLSF	PQIISEGLYAIAVVLSFSRIAYILP	GFQPTNGVGYQPYRVVVLSF
Q13507	P0DTC2-1237	?VVLSF	PQIISEGLYAIAVVLSFSRIAYILP	QPYRVVVLSFELLHAPATVC
Q13507	P0DTC2-1268	?VVLSF	PQIISEGLYAIAVVLSFSRIAYILP	VGYQPYRVVVLSFEL
Q9BYE9	P0DTC3-8	?GVALL?V	QLISVIIGLGVALLLVLVIMTMA	IVGVALLAVF
Q9BYE9	P0DTC3-111	?GVALL	QLISVIIGLGVALLLVLVIMTMA	ASLPFGWLIVGVALL
Q9BYE9	P0DTC1-1023	LLVLV??T	QLISVIIGLGVALLLVLVIMTMA	SLLVLVQSTQWSLFF
P18858	P0DTC1-235	?GKAKK	QRSIMSFFHPKKEGKAKKPEKE ASN	CGNFKVTKGKAKKGA
P18858	P0DTC1-375	?GKAKK	QRSIMSFFHPKKEGKAKKPEKE ASN	ESCGNFKVTKGKAKK
Q8NGD1	P0DTC1-110	???DIQLL	QSQDIQLLVFVLVLIFYF	NKGEDIQLLKSAYENF
Q8NGD1	P0DTC1-317	?DIQLL	QSQDIQLLVFVLVLIFYF	EDIQLLKSAYENFNQ
Q8NGD1	P0DTC1-619	???DIQLL	QSQDIQLLVFVLVLIFYF	KGEDIQLLKSAYENF
Q8NGD1	P0DTC1-1207	???DIQLL	QSQDIQLLVFVLVLIFYF	VNKGEDIQLLKSAYE
P62854	P0DTD1-341	??FDAYV	RDISEASVFDAYVLPKLY	KMFDAYVNTF
P62854	P0DTD1-559	???FDAYV	RDISEASVFDAYVLPKLY	AEVAVKMFDAYVNTFS

P62854	P0DTD1-703	???FDAYV	RDISEASVFDAYVLPKLY	VKMFDAYVNTFS
P62854	P0DTC1-135	???FDAYV	RDISEASVFDAYVLPKLY	VKMFDAYVNTFSST
P62854	P0DTC1-293	???FDAYV	RDISEASVFDAYVLPKLY	DSAEVAVKMFDAYVN
P62854	P0DTC1-804	?FDAYV	RDISEASVFDAYVLPKLY	MFDAYVNTFSSTFNV
P62854	P0DTC1-1156	???FDAYV	RDISEASVFDAYVLPKLY	VAVKMFDAYVNTFSS
P62854	P0DTC1-1194	???FDAYV	RDISEASVFDAYVLPKLY	VKMFDAYVNTFSSTF
O95425	P0DTD1-526	LPTGV	RSAEGPGLPTGVERERGSRKPRRY	YMHHMELPTGV
O95425	P0DTC1-503	LPTGV	RSAEGPGLPTGVERERGSRKPRRY	HHMELPTGVHAGTDL
P19875	P0DTC9-16	?ALLLL	RVALLLLLLVAASRR	LALLLLDRL
P19875	P0DTC9-225	ALLLL	RVALLLLLLVAASRR	ALLLLDRLNQLESKMS
P19875	P0DTC9-262	?ALLLL	RVALLLLLLVAASRR	LALLLLDRLNQLESKM
P19875	P0DTC9-363	ALLLL	RVALLLLLLVAASRR	ALLLLDRLNQLESKMSG
P19875	P0DTC9-439	ALLLL	RVALLLLLLVAASRR	ALLLLDRLNQLESKM
P19875	P0DTC3-139	LLVAA	RVALLLLLLVAASRR	LLVAAGLEAPFLYLY
Q8IX21	P0DTC9-29	SSRSS	SRSLSSRSSLSRHHPEESPLGAKFQ	SQASSRSSSR
Q8IX21	P0DTC9-441	SSRSS	SRSLSSRSSLSRHHPEESPLGAKFQ	ASSRSSSRSRNSSRN
Q8IX21	P0DTC9-448	SSRSS	SRSLSSRSSLSRHHPEESPLGAKFQ	EGSRGGSQASSRSSS
Q8IX21	P0DTC9-501	SSRSS	SRSLSSRSSLSRHHPEESPLGAKFQ	SRGGSQASSRSSSRS
Q15397	P0DTC2-137	AQEKN	STKTAQEKNRFHKNSD	HVTYVPAQEKNF
Q15397	P0DTC2-212	AQEKN	STKTAQEKNRFHKNSD	APHGVVFLHVTYVPAQEKNF
Q15397	P0DTC2-288	AQEKN	STKTAQEKNRFHKNSD	TYVPAQEKNFTTAPAICHDG
Q15397	P0DTC2-565	AQEKN	STKTAQEKNRFHKNSD	VTYVPAQEKNFTTAP
Q15397	P0DTC2-735	AQEKN	STKTAQEKNRFHKNSD	TYVPAQEKNFT
Q5JNZ5-2	P0DTD1-341	??FDAYV	SVFDAYVLPKLYVKLHY	KMFDAYVNTF
Q5JNZ5-2	P0DTD1-559	??FDAYV	SVFDAYVLPKLYVKLHY	AEVAVKMFDAYVNTFS
Q5JNZ5-2	P0DTD1-703	??FDAYV	SVFDAYVLPKLYVKLHY	VKMFDAYVNTFS
Q5JNZ5-2	P0DTC2-6	VKLHY	SVFDAYVLPKLYVKLHY	DDSEPVLKGVKLHYT

Q5JNZ5-2	P0DTC2-100	VKLHY	SVFDAYVLPKLYVKLHY	SEPVLKGVKLHYT
Q5JNZ5-2	P0DTC2-226	VKLHY	SVFDAYVLPKLYVKLHY	FDEDDSEPVLKGVKLHYT
Q5JNZ5-2	P0DTC2-412	VKLHY	SVFDAYVLPKLYVKLHY	VLKGVKLHY
Q5JNZ5-2	P0DTC2-772	VKLHY	SVFDAYVLPKLYVKLHY	CCSCGSCCKFDEDDSEPVLKGVKLHYT
Q5JNZ5-2	P0DTC2-779	VKLHY	SVFDAYVLPKLYVKLHY	DEDDSEPVLKGVKLHY
Q5JNZ5-2	P0DTC2-787	VKLHY	SVFDAYVLPKLYVKLHY	DSEPVLKGVKLHYT
Q5JNZ5-2	P0DTC2-792	VKLHY	SVFDAYVLPKLYVKLHY	EDDSEPVLKGVKLHYT
Q5JNZ5-2	P0DTC2-794	VKLHY	SVFDAYVLPKLYVKLHY	EPVLKGVKLHYT
Q5JNZ5-2	P0DTC2-924	VKLHY	SVFDAYVLPKLYVKLHY	CCMTSCCSCLKGCCSCGSCCKFDEDDSEPVLKGVKLHY T
Q5JNZ5-2	P0DTC1-135	??FDAYV	SVFDAYVLPKLYVKLHY	VKMFDAYVNTFSST
Q5JNZ5-2	P0DTC1-293	??FDAYV	SVFDAYVLPKLYVKLHY	DSAEVAVKMFDAYVN
Q5JNZ5-2	P0DTC1-804	?FDAYV	SVFDAYVLPKLYVKLHY	MFDAYVNTFSSTFNV
Q5JNZ5-2	P0DTC1-1156	??FDAYV	SVFDAYVLPKLYVKLHY	VAVKMFDAYVNTFSS
Q5JNZ5-2	P0DTC1-1194	??FDAYV	SVFDAYVLPKLYVKLHY	VKMFDAYVNTFSSTF
Q9HC29	P0DTD1-125	LAKNV	VGAQALALMLAKNV	AELAKNVSLDNVL
Q9HC29	P0DTD1-242	LAKNV	VGAQALALMLAKNV	AELAKNVSL
Q9HC29	P0DTD1-255	LAKNV	VGAQALALMLAKNV	AEAELAKNV
Q9HC29	P0DTC1-129	LAKNV	VGAQALALMLAKNV	TLVATAEAELAKNVSL
Q9HC29	P0DTC1-166	LAKNV	VGAQALALMLAKNV	AELAKNVSLDNVLST
Q9HC29	P0DTC1-212	LAKNV	VGAQALALMLAKNV	ATAEAELAKNVSLDN
Q9HC29	P0DTC1-666	LAKNV	VGAQALALMLAKNV	LAKNVSLDNVLSTFI
Q9HC29	P0DTC1-787	LAKNV	VGAQALALMLAKNV	LVATAEAELAKNVSL
P62937-3	P0DTD1-970	TVFFD	VNPTVFFDIAVD	KPTETICAPLTVFFD
P62937-3	P0DTD1-1098	TVFFD??VD	VNPTVFFDIAVD	PLTVFFDGRVDGQVD
P62937-3	P0DTD1-1197	TVFFD	VNPTVFFDIAVD	TETICAPLTVFFDGR
P62937-3	P0DTD1-1202	TVFFD??VD	VNPTVFFDIAVD	TICAPLTVFFDGRVD
P62937-3	P0DTD1-1234	TVFFD??VD	VNPTVFFDIAVD	TVFFDGRVDGQVDLF
Q96L11	P0DTD1-77	PILLL	VPILLLLQVKP	QLMCQPILLL
Q96L11	P0DTC1-24	PILLL?Q	VPILLLLQVKP	CQPILLLDQALVSDV
Q96L11	P0DTC1-736	PILLL?Q	VPILLLLQVKP	LMCQPILLLDQALVS
Q96L11	P0DTC1-894	PILLL?Q	VPILLLLQVKP	PILLLDQALVSDVGD
Q96L11	P0DTC1-1028	PILLL?Q	VPILLLLQVKP	SQLMCQPILLLDQAL

Q96L11	P0DTC1-1045	PILLL	VPILLLLQVKP	SVYYSQLMCQPILLL
Q96L11	P0DTC1-1291	PILLL?Q	VPILLLLQVKP	YYSQLMCQPILLLDQ
Q9Y657	P0DTC1-12	RGMVL	WRGMVLARAPVM	LNNLNRGMVLGSLAA
Q9Y657	P0DTC1-418	RGMVL	WRGMVLARAPVM	FIKGLNNLNRGMVLG
Q9Y657	P0DTC1-623	RGMVL	WRGMVLARAPVM	KGLNNLNRGMVLGSL
Q9Y657	P0DTC1-860	RGMVL	WRGMVLARAPVM	NLNRGMVLGSLAATV

Supplementary Table S2: Matching 6-mer linear motifs found in the consensus sequences between SARS-CoV-2 epitopes and autoreactive epitopes found in MS, with the 6-mer motif highlighted in their epitope sequences.

MS epitope ID	COVID-19 epitope ID	Consensus sequence	MS epitope sequence	COVID-19 epitope sequence
P10809-2	P0DTD1-697	??EIPKEE	VVTEIPKEEKDPGM	VEQKIAEIPKEE
P10809-2	P0DTC1-165	?EIPKEE??P	VVTEIPKEEKDPGM	AEIPKEEVKPFITES
P10809-2	P0DTC1-352	??EIPKEE	VVTEIPKEEKDPGM	EKQVEQKIAEIPKEE
P10809-2	P0DTC1-372	??EIPKEE??P	VVTEIPKEEKDPGM	EQKIAEIPKEEVKPF
P10809-2	P0DTC1-628	??EIPKEE??P	VVTEIPKEEKDPGM	KIAEIPKEEVKPFIT
P0DP02	P0DTD1-151	YYRARA	AVYYRARARL	YYRARAGEAANF
P0DP02	P0DTC1-190	?YYRARA	AVYYRARARL	ALQDAYYRARAGEAA
P0DP02	P0DTC1-905	?YYRARA	AVYYRARARL	PPALQDAYYRARAGE
P0DP02	P0DTC1-925	?YYRARA	AVYYRARARL	QDAYYRARAGEAANF
P62861-1	P0DTC1-721	LARAGK	KVHGSLARAGKVRGQTPK	LLARAGKASCTLSEQ
P62861-1	P0DTC1-1283	LARAGK	KVHGSLARAGKVRGQTPK	YPLECIKDLLARAGK
P62736-3	P0DTC1-353	EKSYEL	SLEKSYELPDGQVI	EKSYELQTPFEIKLA
P62736-3	P0DTC1-528	EKSYEL	SLEKSYELPDGQVI	IAWYTERSEKSYELQ
P62736-3	P0DTC1-1076	EKSYEL	SLEKSYELPDGQVI	TERSEKSYELQTPFE
P62736-3	P0DTC1-1251	EKSYEL	SLEKSYELPDGQVI	WYTERSEKSYELQTP
P02489	P0DTD1-57	DDFVEI	DDFVEIHGKHNERQD	LLLDDFVEI
P02489	P0DTD1-137	DDFVEI	DDFVEIHGKHNERQD	LLLDDFVEII
P02489	P0DTD1-195	DDFVEI	DDFVEIHGKHNERQD	LDDFVEIIKSQDLSV
P02489	P0DTD1-775	DDFVEI	DDFVEIHGKHNERQD	CSVIDLLLDDFVEII
P02489	P0DTD1-782	DDFVEI	DDFVEIHGKHNERQD	DDFVEIIKSQDLSVV
P02489	P0DTD1-797	DDFVEI	DDFVEIHGKHNERQD	DLLLDDFVEIIKSQD

P02489	P0DTD1-1011	DDFVEI	DDFVEIHGKHNERQD	LLDDFVEIIKSQDLS
P02489	P0DTD1-1255	DDFVEI	DDFVEIHGKHNERQD	VIDLLLDDFVEIIKS
P09972-8	P0DTC5-3	VLAAVY?	VLAAVYKALSDHHVY	FVLAAVYRI
P09972-8	P0DTC5-52	VLAAVY?	VLAAVYKALSDHHVY	TLACFVLAAVYRINW
P09972-8	P0DTC5-129	VLAAVY?	VLAAVYKALSDHHVY	VLAAVYRINWITGGI
P62736-6	P0DTC1-353	EKSYEL	SSSSLEKSYELPDGQVI	EKSYELQTPFEIKLA
P62736-6	P0DTC1-528	EKSYEL	SSSSLEKSYELPDGQVI	IAWYTERSEKSYELQ
P62736-6	P0DTC1-1076	EKSYEL	SSSSLEKSYELPDGQVI	TERSEKSYELQTPFE
P62736-6	P0DTC1-1251	EKSYEL	SSSSLEKSYELPDGQVI	WYTERSEKSYELQTP
Q16695-3	P0DTC8-3	ARKSAP	VARKSAPATGGVKKPHRYRPGTVAL	RVGARKSAPL
Q16695-3	P0DTC8-9	ARKSAP	VARKSAPATGGVKKPHRYRPGTVAL	SKWYIRVGARKSAPL
Q16695-3	P0DTC8-11	ARKSAP	VARKSAPATGGVKKPHRYRPGTVAL	YIRVGARKSAPLIEL
Q16695-3	P0DTC8-12	ARKSAP	VARKSAPATGGVKKPHRYRPGTVAL	ARKSAPLIELCVDEA
Q16695-3	P0DTC8-68	ARKSAP	VARKSAPATGGVKKPHRYRPGTVAL	GARKSAPLIELCVDE
Q16695-3	P0DTC8-82	ARKSAP	VARKSAPATGGVKKPHRYRPGTVAL	RVGARKSAPLIELCV
P25440	P0DTD1-424	KKGAKL	KKGAKLAALQGS	TPSFKKGAKLLHKPI
P25440	P0DTD1-580	KKGAKL	KKGAKLAALQGS	PDLNGDVVAIDYKHYTPSFKKGAKLLHK PIVWHVNNATN
P25440	P0DTC1-518	KKGAKL	KKGAKLAALQGS	HYTPSFKKGAKLLHK
P25440	P0DTC1-1006	KKGAKL	KKGAKLAALQGS	SFKKGAKLLHKPIVW
P57073	P0DTC9-3	APSASA	KSAPSASASPTE	APSASAFFGM
P57073	P0DTC9-4	APSASA	KSAPSASASPTE	AQFAPSASA
P57073	P0DTC9-5	APSASA	KSAPSASASPTE	AQFAPSASAFFGMSR
P57073	P0DTC9-38	APSASA	KSAPSASASPTE	FAPSASAFF
P57073	P0DTC9-73	APSASA	KSAPSASASPTE	WPQIAQFAPSASAFF
P57073	P0DTC9-103	APSASA	KSAPSASASPTE	QFAPSASAFFGMSRI
P57073	P0DTC9-107	APSASA	KSAPSASASPTE	AQFAPSASAF
P57073	P0DTC9-178	APSASA	KSAPSASASPTE	QFAPSASAFF
P57073	P0DTC9-440	APSASA	KSAPSASASPTE	APSASAFFGMSRIGM
P57073	P0DTC9-461	APSASA	KSAPSASASPTE	IAQFAPSASAFFGMS
Q8N729	P0DTC9-16	LALLLL	APASRPRLALLLLLLLLPLP	LALLLLDRL
Q8N729	P0DTC9-42	LALLLL	APASRPRLALLLLLLLLPLP	GDAALALLLLDRLNQL
Q8N729	P0DTC9-93	LALLLL	APASRPRLALLLLLLLLPLP	DAALALLLLDRLNQL

Q8N729	P0DTC9-118	LALLLL	APASRPRLALLLLLLLLPLP	PARMAGNGGDAALALLLLDRLNQLESK MSGKGQQQQGQ
Q8N729	P0DTC9-120	LALLLL	APASRPRLALLLLLLLLPLP	AALALLLLDRLNQLE
Q8N729	P0DTC9-157	LALLLL	APASRPRLALLLLLLLLPLP	AGNGGDAALALLLLD
Q8N729	P0DTC9-216	LALLLL	APASRPRLALLLLLLLLPLP	AALALLLLDRLNQLES
Q8N729	P0DTC9-221	LALLLL	APASRPRLALLLLLLLLPLP	AGNGGDAALALLLLDR
Q8N729	P0DTC9-224	LALLLL	APASRPRLALLLLLLLLPLP	ALALLLLDRLNQLESK
Q8N729	P0DTC9-229	LALLLL	APASRPRLALLLLLLLLPLP	ARMAGNGGDAALALLLLDRLNQLESK MSGKG
Q8N729	P0DTC9-231	LALLLL	APASRPRLALLLLLLLLPLP	DAALALLLLDRLNQLE
Q8N729	P0DTC9-245	LALLLL	APASRPRLALLLLLLLLPLP	GGDAALALLLLDRLNQ
Q8N729	P0DTC9-249	LALLLL	APASRPRLALLLLLLLLPLP	GNGGDAALALLLLDRL
Q8N729	P0DTC9-262	LALLLL	APASRPRLALLLLLLLLPLP	LALLLLDRLNQLESKM
Q8N729	P0DTC9-277	LALLLL	APASRPRLALLLLLLLLPLP	MAGNGGDAALALLLLD
Q8N729	P0DTC9-282	LALLLL	APASRPRLALLLLLLLLPLP	NGGDAALALLLLDRLN
Q8N729	P0DTC9-313	LALLLL	APASRPRLALLLLLLLLPLP	RMAGNGGDAALALLLL
Q8N729	P0DTC9-339	LALLLL	APASRPRLALLLLLLLLPLP	SSRGTSPARMAGNGGDAALALLLLDRL NQLESKMSGKGQ
Q8N729	P0DTC9-359	LALLLL	APASRPRLALLLLLLLLPLP	AALALLLLDRLNQLESKMS
Q8N729	P0DTC9-394	LALLLL	APASRPRLALLLLLLLLPLP	MAGNGGDAALALLLLDR
Q8N729	P0DTC9-417	LALLLL	APASRPRLALLLLLLLLPLP	RMAGNGGDAALALLLLDRL
Q8N729	P0DTC9-438	LALLLL	APASRPRLALLLLLLLLPLP	ALALLLLDRLNQLES
Q8N729	P0DTC9-453	LALLLL	APASRPRLALLLLLLLLPLP	GGDAALALLLLDRLN
Q8N729	P0DTC9-456	LALLLL	APASRPRLALLLLLLLLPLP	GNGGDAALALLLLDR
Q8N729	P0DTC9-480	LALLLL	APASRPRLALLLLLLLLPLP	MAGNGGDAALALLLL
Q9NWB7	P0DTC2-99	RKSNLK	KLKLLRYEEEFLRKSNLKAPSRHYF	RKSNLKPFERDISTE
Q9NWB7	P0DTC2-119	Y?????RKSNLK	KLKLLRYEEEFLRKSNLKAPSRHYF	YLYRLFRKSNLKPFE
Q9NWB7	P0DTC2-159	???RKSNLK	KLKLLRYEEEFLRKSNLKAPSRHYF	RLFRKSNLK
Q9NWB7	P0DTC2-242	?Y?????RKSNLK	KLKLLRYEEEFLRKSNLKAPSRHYF	KVGGNYNYLYRLFRKSNLKP
Q9NWB7	P0DTC2-272	???RKSNLK	KLKLLRYEEEFLRKSNLKAPSRHYF	RLFRKSNLKPFERDISTEIY
Q9NWB7	P0DTC2-330	?RKSNLK	KLKLLRYEEEFLRKSNLKAPSRHYF	FRKSNLKPFERDIST
Q9NWB7	P0DTC2-452	???RKSNLK	KLKLLRYEEEFLRKSNLKAPSRHYF	RLFRKSNLKP
Q9NWB7	P0DTC2-572	?Y?????RKSNLK	KLKLLRYEEEFLRKSNLKAPSRHYF	YNYLYRLFRKSNLKP

Q9NWB7	P0DTC2-576	???RKSNLK	KLKLLRYEEEFLRKSNLKAPSRHYF	YRLFRKSNLKPFERD
Q9NWB7	P0DTC2-713	?Y?????RKSNLK	KLKLLRYEEEFLRKSNLKAPSRHYF	VGGNYNYLYRLFRKSNLKP
Q9NWB7	P0DTC2-725	?Y?????RKSNLK	KLKLLRYEEEFLRKSNLKAPSRHYF	LDSKVGGNYNYLYRLFRKSNLKPF
Q9NWB7	P0DTC2-742	??RKSNLK	KLKLLRYEEEFLRKSNLKAPSRHYF	LFRKSNLKPFERDIS
Q9NWB7	P0DTC2-951	?Y?????RKSNLK	KLKLLRYEEEFLRKSNLKAPSRHYF	GGNYNYLYRLFRKSNLKPFERDI
Q9NWB7	P0DTC2-1025	?RKSNLK	KLKLLRYEEEFLRKSNLKAPSRHYF	FRKSNLKPFERD
Q9NWB7	P0DTC2-1115	???RKSNLK	KLKLLRYEEEFLRKSNLKAPSRHYF	RLFRKSNLKPFE
Q9NWB7	P0DTC2-1196	?RKSNLK	KLKLLRYEEEFLRKSNLKAPSRHYF	FRKSNLKPFERDISTEIYQA
Q9NWB7	P0DTC2-1204	?Y?????RKSNLK	KLKLLRYEEEFLRKSNLKAPSRHYF	GGNYNYLYRLFRKSNLKPFE
Q9NTJ3	P0DTC2-24	KVEAEV	KVEAEVKRLH	RLDKVEAEV
Q9NTJ3	P0DTC2-38	KVEAEV?	KVEAEVKRLH	DILSRLDKVEAEVQI
Q9NTJ3	P0DTC2-292	KVEAEV?	KVEAEVKRLH	VLNDILSRLDKVEAEVQIDR
Q9NTJ3	P0DTC2-362	KVEAEV?	KVEAEVKRLH	LSRLDKVEAEVQIDR
Q9NTJ3	P0DTC2-508	KVEAEV	KVEAEVKRLH	LNDILSRLDKVEAEV
Q9NTJ3	P0DTC2-755	KVEAEV?	KVEAEVKRLH	NTLVKQLSSNFGAISSVLNDILSRLDKVEAEVQIDRLITGRLQSLQTY
Q9NTJ3	P0DTC2-933	KVEAEV?	KVEAEVKRLH	LSRLDKVEAEVQIDRLITGR
Q9NTJ3	P0DTC2-1069	KVEAEV?	KVEAEVKRLH	LSRLDKVEAEVQID
Q9NTJ3	P0DTC2-1243	KVEAEV?	KVEAEVKRLH	RLDKVEAEVQIDRLI
P19875	P0DTC3-125	LLLVAA	RVALLLLLLVAASRR	HLLLVAAGLEAPFLY
P19875	P0DTC3-169	LLLVAA	RVALLLLLLVAASRR	YSHLLLVAAGLEAPF
